# Novel ATR/PARP1 Dual Inhibitors Demonstrate Synergistic Antitumor Efficacy in Triple‐Negative Breast Cancer Models

**DOI:** 10.1002/advs.202501916

**Published:** 2025-06-16

**Authors:** Yuan Gao, Jiawei Zhou, Chen‐Chen Wang, Zong‐Hao Wang, Nian‐Dong Mao, Meng‐Lan He, Peng‐Peng Zhang, Ping Huang, Guo‐Wei Ye, Yu‐Qing Zhang, Feng‐Hui Tang, Hang Zhang, Tian Xie, Xiang‐Yang Ye

**Affiliations:** ^1^ Clinical Research Institute Zhejiang Provincial People's Hospital (Affiliated People's Hospital) Hangzhou Medical College Hangzhou Zhejiang Province 310000 China; ^2^ School of Pharmacy Hangzhou Normal University Hangzhou Zhejiang Province 310000 China; ^3^ Key Laboratory of Elemene Class Anti‐Cancer Chinese Medicines Engineering Laboratory of Development and Application of Traditional Chinese Medicines Collaborative Innovation Center of Traditional Chinese Medicines of Zhejiang Province Hangzhou Normal University Hangzhou Zhejiang Province 310000 China; ^4^ College of Life and Environmental Sciences Hangzhou Normal University Hangzhou Zhejiang Province 310000 China; ^5^ Department of Pharmacy Zhejiang Provincial People's Hospital (Affiliated People’ s Hospital) Hangzhou Medical College Hangzhou Zhejiang Province 310000 China; ^6^ School of Basic Medical Science Hangzhou Normal University Hangzhou Zhejiang Province 310000 China

**Keywords:** Anticancer, ATR, Dual Inhibitor, PARP1, TNBC

## Abstract

Concomitant inhibition of ataxia telangiectasia and Rad3‐related protein (ATR) and poly ADP‐ribose Polymerase (PARP) pathways is a promising strategy in cancer therapy, potentially expanding the clinical utility of ATR inhibitor (ATRi) and PARP inhibitor (PARPi). A novel series of ATR/PARP1 dual inhibitors is developed through the pharmacophore fusion of AZD6738 and Olaparib. Among them, **B8** emerges as the most promising candidate, exhibiting potent ATR (IC_50_: 17.3 nM) and PARP1 (IC_50_: 0.38 nM) inhibition. **B8** effectively reduced cell viability, induced apoptosis, and caused G2/M cell cycle arrest in TNBC cells. Additionally, **B8** significantly impaired TNBC colony formation, migration, and invasion. Mechanistically, **B8** induces DNA damage, evidenced by increased *γ*H2AX levels. In in vivo studies, **B8** suppressed tumor growth more effectively than the combination in MDA‐MB‐468 xenografted mice, with no significant body weight loss. **B8** also enhanced *γ*H2AX expression in tumor tissues. These findings confirm the synergistic effects of ATR/PARP1 co‐inhibition and highlight the potential of this novel inhibitor class for TNBC therapy.

## Graphical abstract

Novel ATR/PARP1 dual inhibitors are developed for the first time. Advanced lead **B8** effectively reduces cell viability in vitro and suppresses tumor growth in vivo, with better potency than ATRi and PARPi alone or in combination. Mechanistically, **B8** induces apoptosis, arrests the cell cycle, and inhibits cancer cells colony formation, migration, and invasion.

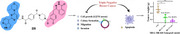

## 1 Introduction

Triple‐negative breast cancer (TNBC), characterized by a lack of the expression of hormone receptors, both estrogen (ER) and progesterone (PR) receptors, and no amplification of human epidermal growth factor receptor 2 (HER2), is the subtype of breast cancer with the poorest prognosis and the highest mortality rate.^[^ ^1^, ^2^, ^3^ ^]^ The lack of effective therapeutic targets in this subtype leads to the high recurrence rates, aggressive tumor behavior, and poor prognosis of TNBC patients.^[^ ^4^, ^5^ ^]^ Currently, the clinical treatment of TNBC is mainly based on surgery, systemic radiotherapy, and chemotherapy. However, a significant proportion of TNBC patients are resistant to radiotherapy, resulting in a lack of effective treatment options available for this patient population.^[^ ^6^, ^7^, ^8^ ^]^ Thus, chemotherapy remains the mainstay of clinical management of TNBC. Despite numerous studies focusing on the development of inhibitors against different targets, such as PARP, ATR, mitogen‐activated protein kinase (MAPK), androgen Receptor (AR), cyclin‐dependent kinases (CDKs), and epidermal growth factor receptor (EGFR) signaling pathway,^[^ ^9^, ^10^, ^11^, ^12^, ^13^, ^14^, ^15^ ^]^ these agents have shown limited efficacy when used alone and are prone to drug resistance. Therefore, there is an urgent need for the development of novel compounds that can inhibit multiple effective targets to enhance the therapeutic efficacy against TNBC in the current research landscape.

There have been several new drugs approved for the treatment of TNBC in recent years. For example, Olaparib (Lynparza) and Talazoparib (Talzenna) are approved for BRCA‐mutated HER2‐negative metastatic breast cancer (including TNBC) with improved progression‐free survival (PFS) in trials (e.g., OlympiAD for olaparib, EMBRACA for talazoparib). However, these therapies are restricted to BRCA‐mutated TNBC (∼10%–15% of cases), with potential resistance mechanisms (e.g., BRCA reversion mutations). Atezolizumab (Tecentriq) is an immune checkpoint inhibitor that blocks PD‐L1 on tumor/immune cells, restoring T‐cell‐mediated antitumor immunity. It was approved for the combination with nab‐paclitaxel for PD‐L1‐positive (≥1% immune cell staining) unresectable/metastatic TNBC (based on IMpassion130 trial). The drawback is that only ∼40% of TNBCs express PD‐L1, and responses vary due to tumor microenvironment heterogeneity. Sacituzumab govitecan (Trodelvy) is approved for metastatic TNBC after ≥2 prior therapies (based on the ASCENT trial). However, toxicity (e.g., neutropenia, diarrhea) exists, and predictive biomarkers are under study. In summary, these therapies represent a shift toward precision medicine in TNBC. However, the resistance is the major issue that needs to be addressed urgently.

**Figure 1:**
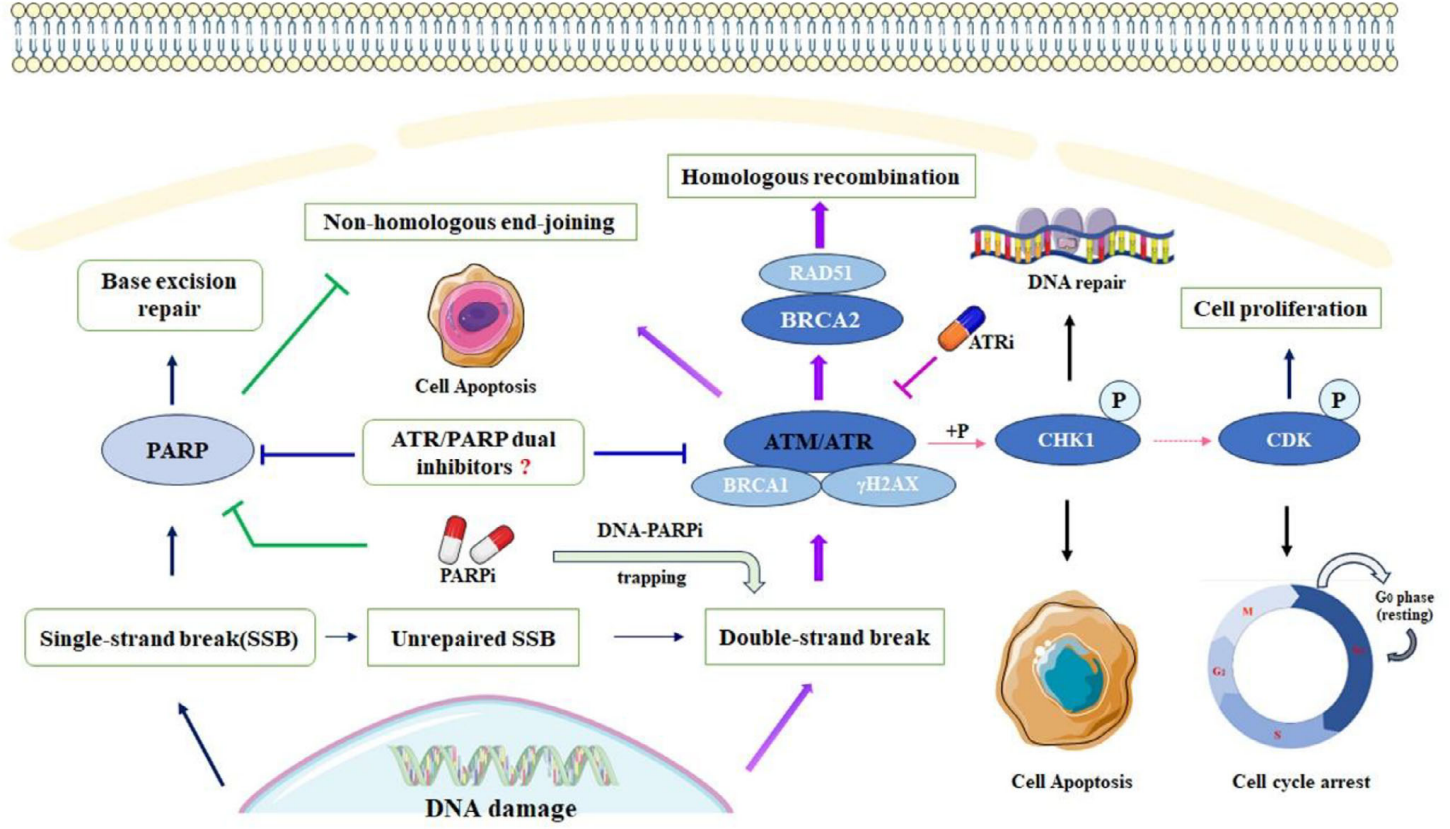
Biological functions of PARP1 and ATR.

**Figure 2:**
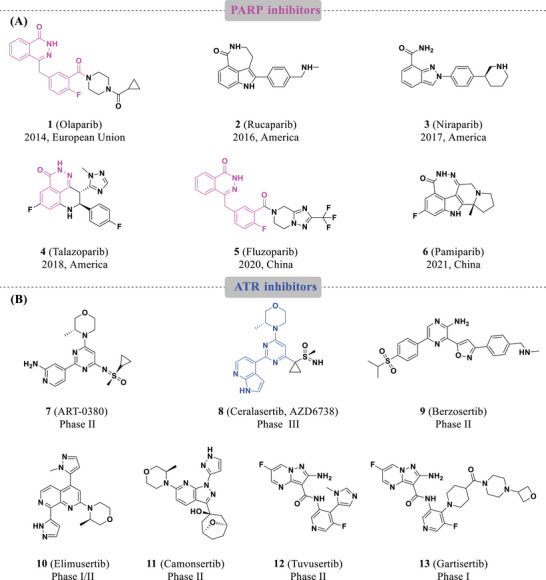
Chemical structures of PARPi and ATRi. A) PARPi approval drugs (**1‐6**); B) representative ATRi clinical candidates (**7‐13**).

PARPi has demonstrated efficacy in the treatment of TNBC and ovarian cancer (OC), particularly in tumors exhibiting homologous recombination deficiency (HRD), including but not limited to those with BRCA1/2 mutations. It should be noted that only a subset of TNBC (approximately 10%∼20%) and OC (approximately 15%∼20%) cases harbor germline or somatic BRCA1/2 mutations.^[^ ^16^, ^17^ ^]^ BRCA mutant cells are deficient in effectively repairing DNA double‐strand breaks (DSBs) through homologous recombination (HR). Therefore, DSBs must be addressed and repaired by alternative repair pathways. Inhibition of PARP enzymes leads to a synthetic lethality effect, resulting in erroneous repair of DNA damage and ultimately causing the death of tumor cells (**Figure** **1**).^[^ ^18^ ^]^ Several PARPi have been approved for clinical use: Olaparib (**1**), Rucaparib (**2**), Niraparib (**3**), Talazoparib (**4**), Fluzoparib (**5**), and Pamiparib (**6**) (**Figure** **2**).^[^ ^19^ ^]^ Olaparib is the first approved small‐molecule PARP1 inhibitor. Despite the clinical success of PARPi, many patients ultimately develop resistance to these agents. Furthermore, the efficacy of PARPi in BRCA wild‐type cells is limited.^[^ ^20^, ^21^ ^]^ As such, developing combination strategies with other agents that disrupt HR repair is of significant importance for enhancing the effectiveness of PARPi in the treatment of TNBC.

Ataxia telangiectasia and Rad3‐related protein (ATR) is a serine/threonine protein kinase that plays a crucial role in regulating the activation of cell‐cycle checkpoints and the DNA damage response (DDR) induced by DNA replication stress. (Figure 1).^[^ ^22^ ^]^ When DNA replication stress occurs in cells, such as stalling of the replication fork progression, ATR is activated by sensing the accumulation of single‐stranded DNA (ssDNA) formed at the stalled replication forks, leading to stabilization of the replication fork and prevention of DNA double‐strand breaks.^[^ ^23^ ^]^ If an ATR function is impaired, it can result in the accumulation of replication stress, ultimately causing genomic instability and cell death. Additionally, ATR activates a series of downstream signaling pathways by phosphorylating CHK1, which induces cell‐cycle arrest, providing time for DNA repair.^[^ ^24^, ^25^ ^]^ Conversely, blockade of the ATR/CHK1 signaling pathway can allow cells with damaged DNA to enter mitosis, resulting in genomic abnormalities and chromosomal aberrations in the daughter cells, ultimately leading to cell death.^[^ ^26^ ^]^ Indeed, ATR inhibition has been shown to exert synthetic lethality in various cancers, including TNBC, by combining with mechanisms associated with deficiencies in DNA repair pathways, such as mutations of BRCA1/2, TP53, and ATM.^[^ ^26^, ^27^, ^28^ ^]^ The key role of ATR positions it as an attractive therapeutic target in cancers with elevated replication strcess or DNA repair deficiency.^[^ ^28^ ^]^ Currently, several ATR inhibitors (ATRi) have been advanced to clinical development (Figure 2B): ART0380 (**7**), Ceralasertib (AZD6738, **8**), Berzosertib (**9**), Elimusertib (**10**), Camonsertib (**11**), Tuvusertib (**12**), and Gartisertib (**13**).^[^ ^19^ ^]^ Increasing evidences from both experimental and clinical settings suggest that ATRi could have potential anti‐cancer effects as monotherapies or in combination with other targeted drugs to overcome drug resistance and promote antitumor immunity.^[^ ^29^, ^30^, ^31^, ^32^, ^33^, ^34^ ^]^ For example, the combination of ATRi VE‐821 and Olaparib was proven to be beneficial for patients with ATM‐deficient tumors.^[^ ^30^ ^]^ It has also been discovered that the combination of AZD6738 and Olaparib could enhance genomic instability and induce cell death in ATM‐deficient cancer cells.^[^ ^31^ ^]^ Additionally, the combination of DS‐8201 (a HER2‐targeted antibody‐drug conjugate agent), Elimusertib, and Olaparib exerted significant synergistic antitumor activity.^[^ ^32^ ^]^ Furthermore, it was reported that AZD6738 is likely to enhance the anti‐cancer effects of PARPi (Olaparib, Talazoparib, or Veliparib) in homologous recombination repair‐deficient cell lines.^[^ ^33^ ^]^ In addition, a growing number of ATRi have been demonstrated to induce synthetic lethality in various cancer models, including breast cancer, ovarian cancer, and pancreatic cancer, particularly when used in combination with PARPi.^[^ ^34^, ^35^, ^36^, ^37^ ^]^ It is now well established that the combination of ATRi and PARPi demonstrates enhanced anti‐tumor efficacy compared to either inhibitor alone. However, there was no report on a single molecule capable of simultaneously targeting both ATR and PARP.

The above backgrounds on the synergistic lethal effects of PARPi and ATRi in TNBC, along with our accumulated experience from multiple dual inhibitor studies,^[^ ^38^, ^39^, ^40^ ^]^ have inspired us to design a series of dual inhibitors capable of simultaneously targeting ATR and PARP1. This series of dual‐target inhibitors may exhibit enhanced anti‐tumor effects by synergistically intervening in both PARP1 and ATR‐associated signaling pathways. To achieve this goal, we employed bioinformatics analysis, molecular docking, computer‐aided design, and a pharmacophore fusion strategy to develop a range of dual inhibitors for ATR and PARP. Following evaluations of the promising compounds for their anti‐TNBC activities and pharmacological mechanisms in both in vitro and in vivo studies. Herein, we report for the first time about a novel ATR/PARP1 dual inhibitor with promising antitumor effects in *BRCA^WT^* TNBC.

## 2 Results and Discussion

### 2.1 Bioinformatics Analysis

**Figure 3:**
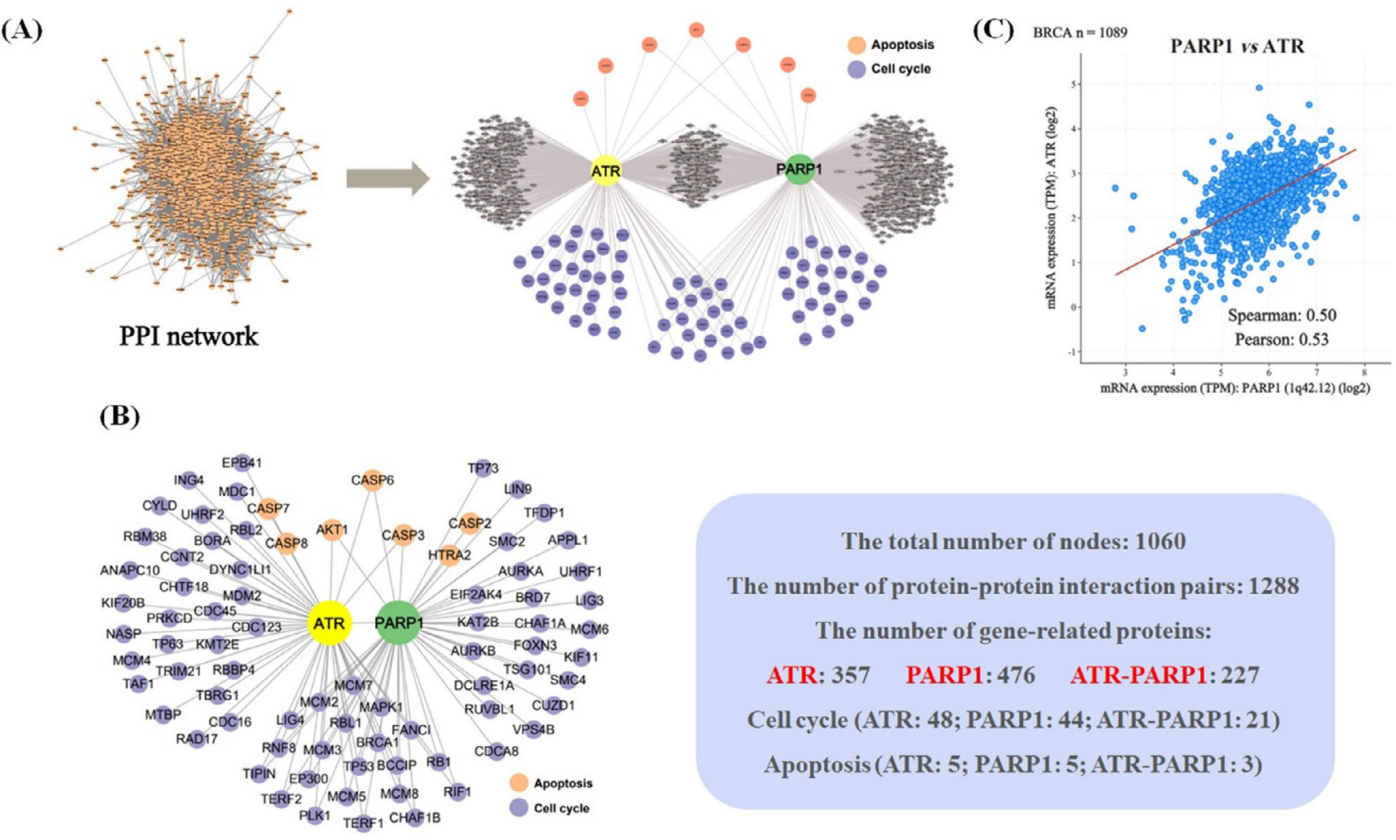
Bioinformatics analysis of the relationship between ATR and PARP1. A) The workflow of bioinformatics analyses of ATR‐PARP1 and cluster analysis network diagram of ATR and PARP1 interaction proteins related to cell cycle and apoptosis; B) Predicted ATR‐ and PARP1‐related proteins involved in the regulation of cell cycle and apoptosis, respectively; C) Correlation between the mRNA expression of ATR and PARP1 in breast cancer samples (*n* = 1089).

To confirm the correlation between ATR and PARP in their respective biological functions, first, a protein–protein interaction (PPI) network was established using “The Cancer Genome Atlas (TCGA)” database. From this comprehensive network (see Supporting Information‐1 (SI‐1) for details PPI network analysis), proteins associated with ATR and PARP1 were identified (**Figure** **3**A). Specifically, 357 proteins that potentially interact with ATR and 476 proteins that potentially interact with PARP1 were identified (Figure 3A). These proteins were categorized into two hubs: the apoptotic hub and the cell cycle hub. The overlapping proteins relevant to both ATR and PARP1 were subsequently identified and assigned to these two hubs (Figure 3B). Further analysis revealed a total of 24 proteins that are associated with both ATR and PARP1, including three proteins, AKT1, CASP3, and CASP6, from the apoptosis hub, as well as 21 proteins from the cell cycle hub (Figure 3B).

Subsequently, we used an online cancer genomic website to analyze the relevance between ATR and PARP1.^[^ ^41^ ^]^ The analysis revealed a positive correlation in the mRNA expression levels of these two proteins in breast cancer (Figure 3C). There is no literature report of such a correlation in TNBC. The positive correlation of these two proteins in other cancer types such as cholangiocarcinoma, esophageal cancer, head and neck squamous cell carcinoma (HNSCC), hepatocellular carcinoma (HCC), and lung squamous cell carcinoma (See Figure S1, Supporting Information). This finding convinces that ATR and PARP1 play interconnected roles in cell cycle regulation and apoptosis in breast cancer, highlighting their significant interplay within the pathology of such disease. The above results provide theoretical support for the design of ATR/PARP1 dual inhibitors.

### 2.2 ATRi and PARPi synergistically inhibit the proliferation of TNBC Cells

To explore the synthetic lethality induced by the combination of ATRi and PARPi, AZD6738 and Olaparib were chosen for study for anti‐proliferative effects against three TNBC cell lines, namely, MDA‐MB‐231, MDA‐MB‐468, and MDA‐MB‐436 alone or in combination. The combination of AZD6738 and Olaparib synergistically inhibits the proliferation of three TNBC cells compared to monotherapy (**Table** **1**). The results further justified the conceptual idea that ATR/PARP1 dual inhibitors could have potential beneficial effects for combating TNBC.

**Table 1:** Inhibitory activities of Olaparib, AZD6738, and the combination of AZD6738 and Olaparib on MDA‐MB‐231, MDA‐MB‐468, and MDA‐MB‐436.

Compd	IC_50_ (µM)^a)^		
	MDA‐MB‐231	MDA‐MB‐468	MDA‐MB‐436
**1** (Olaparib)	21.87 ± 2.36	10.54 ± 0.59	4.53 ± 0.21
**8** (AZD6738)	12.57 ± 1.43	18.58 ± 1.75	3.84 ± 0.52
**1** + **8** (1:1)	3.26 ± 1.42	1.77 ± 0.37	0.89 ± 0.05

^a)^Assays were performed in replicate (n = 3); IC_50_ values are shown as mean ± SD.

### 2.3 Design of Novel ATR/PARP1 Dual Inhibitors

**Figure 4:**
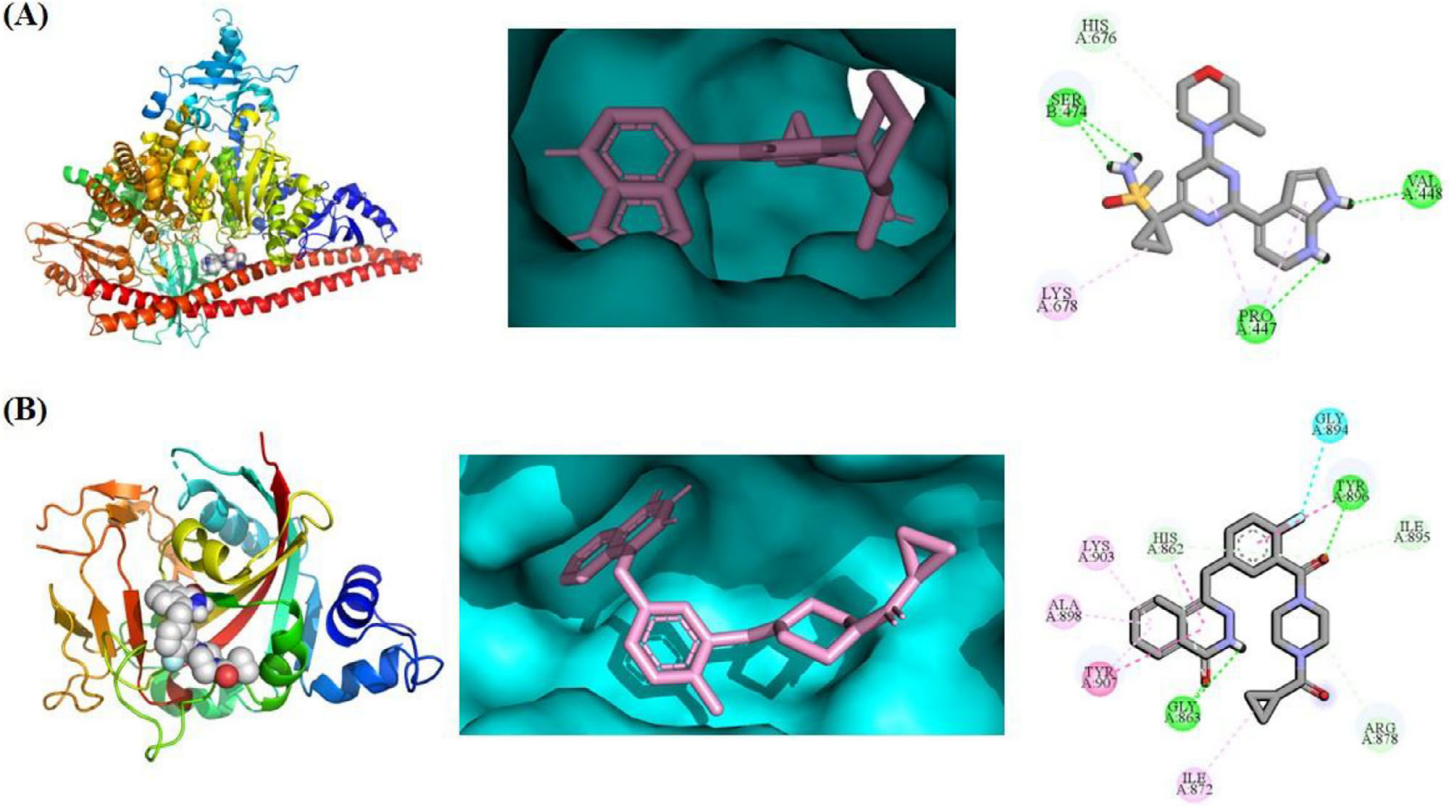
A) Binding mode of AZD6738 docked with rationally designed PI3Kα mutant that mimics ATR (PDB code: 5UL1). B) Co‐crystal structure of Olaparib in complex with PARP1 (PDB code: 5DS3). Green dashed lines indicate hydrogen bond interactions. The carbons of small molecules are in black. The oxygen atoms of small molecules are in red. The nitrogen atoms are in purple. The sulphur atom is in yellow, while the fluorine atom is in pale blue.

**Figure 5:**
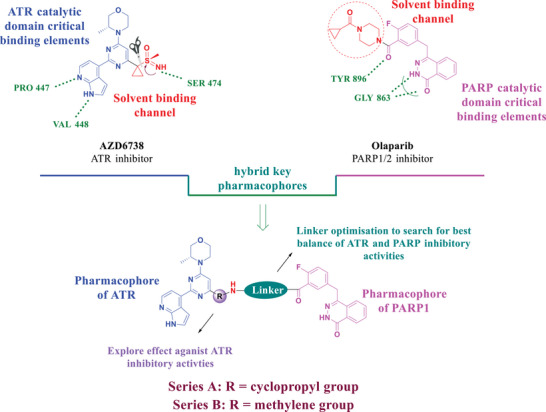
Design strategy of ATR/PARP1 dual inhibitors based on the pharmacophores of AZD6738 and Olaparib.

To optimize the design of dual inhibitors targeting ATR and PARP1, we began by analyzing the available co‐crystal structures of small‐molecule inhibitors in complex with both proteins. The results of this analysis informed us of the key pharmacophores that should be preserved in the development of novel dual inhibitors. Sim et al rationally constructed a PI3Ka mutant as a mimetic ATR protein, then successfully obtained several co‐crystal structures of small molecule ATR inhibitors in complex with PI3Kα mutant protein.^[^ ^41^ ^]^ AZD6738 was then docked with such mimetic ATR protein using one of the reported structures (PDB code: 5UL1^[^ ^42^ ^]^) for analysis (**Figure** **4A**). 1*H*‐pyrrolo[*2,3‐b*]pyridine moiety of AZD6738 binds to the mimetic ATR protein and, respectively, forms two key hydrogen bonds with VAL448 and PRO447, while the N atom of the sulfanone moiety forms another key hydrogen bond with SER474. The cyclopropyl (the red region of AZD6738 in **Figure** **5**) appears to be in a place with extra space where bigger groups or side chains could be tolerated without losing affinity with ATR. This site was then identified as the attachment point for designing dual inhibitors (Figures 4A and 5).

Of six approved PARP1 inhibitors, three contain phthalazin‐1(2*H*)‐one motif as the key pharmacophore (i.e., Olaparib, Fluzoparib, and Talazoparib). We decided to use the first PARPi approval drug, Olaparib, and its key pharmacophore phthalazin‐1(2*H*)‐one, as our design starting point. The co‐crystal structure of Olaparib in complex with PARP1 protein (PDB code: 5DS3)^[^ ^43^ ^]^ was then analyzed (Figure 4B). The carbonyl group and one of the nitrogen in phthalazin‐1(2*H*)‐one offer as both hydrogen donor and acceptor to GLY863 through bidentate chelation. The carbonyl group attached to the phenyl ring has hydrogen bond with TYR896. These key interactions should be considered while designing ATR /PARP1 dual inhibitors.

There are several excellent articles published in recent years, disclosing dual inhibitors containing PARP1.^[^ ^44^, ^45^, ^46^ ^]^ Based on the above analysis and what we have accumulated in the dual inhibitor drug discovery field, we designed dual inhibitors by connecting the blue portion of AZD6738 to the purple portion of Olaparib via a linker (Figure 5). In order to simplify the synthesis, the iminosulfanone functional group was replaced with an amino functional group, while the cyclopropyl group was preserved or replaced with a methylene group (R group in Figure 5).

### 2.4 Chemistry

**Scheme 1:**
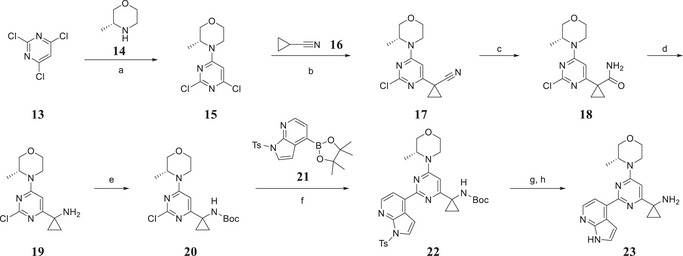
Synthesis of key intermediate **23**. *^a^* Reagents and conditions: a) TEA, EtOH, rt, 16 h, 66.2%; b) NaHMDS, Toluene, 0 °C ∼ rt, 2.5 h, 56.4%; c) KOH, H_2_O_2_ (30%), MeOH/DCM (1:1), 40 °C, overnight, 56.3%; d) NaOH, NaClO·5 H_2_O, H_2_O, rt, 16 h, 74.6%; e) Boc_2_O, DMAP, TEA, DCM, rt, overnight, 55%; f) K_2_CO_3_, Pd(dppf)Cl_2_, 1,4‐Dioxane/H_2_O, 100 °C, 4 h, 57.3%; g) HCl in 1,4‐Dioxane (4 M), MeOH, rt, 2 h, 93%; (h) aqueous NaOH, THF, 1,4‐Dioxane, 75 °C, overnight, 51.9%.

**Scheme 2:**
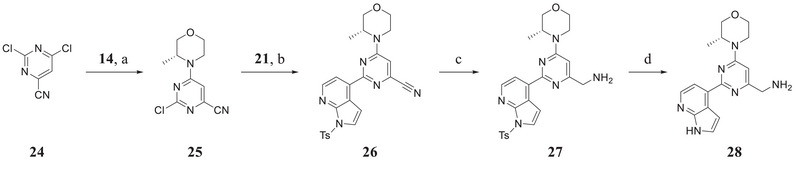
Synthesis of key intermediate **28**. Reagents and conditions: a) TEA, DCM, rt, 2 h, 79.7%; b) K_2_CO_3_, Pd(dppf)Cl_2_, 1,4‐Dioxane/H_2_O, 100 °C, 4 h, 87.1%; c) H_2_, Raney Nickel, NH_3_/MeOH, rt, 16 h, 39.6%; d) aqueous NaOH, THF, 1,4‐Dioxane, 75 °C, overnight, 73.4%.

Initially, the chiral iminosulfanone of AZD6738 was replaced with an amino group in a dual inhibitor design, ending up with Series A (R is a cyclopropyl group) (**Table** **2**). Further simplifying the synthesis of target molecules by replacing the cyclopropyl group with a methylene group provided Series B (R is a methylene group). Such a design avoided the chiral iminosulfanone moiety synthesis and greatly facilitated the quick access of the target molecules. **Scheme** **1** describes the synthesis of key intermediate **23**. The commercially available starting material **13** reacted with **14** in the presence of triethylamine (TEA) to yield intermediate **15**.^[^ ^47^ ^]^ The α‐position of cyclopropane carbonitrile **16** was deprotonated in the presence of NaHMDS, and the resulting anion reacted with **15** to yield intermediate **17**. The cyanide was transformed to amide **18**, followed by Hofmann rearrangement to afford cycloproyl amine derivative **19**. The amino group of **19** was protected with Boc to afford **20**, which further reacted with **21** under standard Suzuki coupling reaction conditions to afford **22**. The deprotection of the Boc group and the tosyl group (Ts‐) affords key intermediate **23** (Scheme 1). It should be noted that it took several steps to install the cyclopropyl amino moiety in intermediate **23**. In order to simplify the synthesis, we also designed intermediate **28**, in which the cyclopropyl group was changed to a methylene group. Thus, **28** was synthesized from **14**, **21,** and **24** in four steps: SN2 displacement, Suzuki reaction, cyanide reduction, and Ts‐deprotection (**Scheme** **2**).

**Figure 6:**
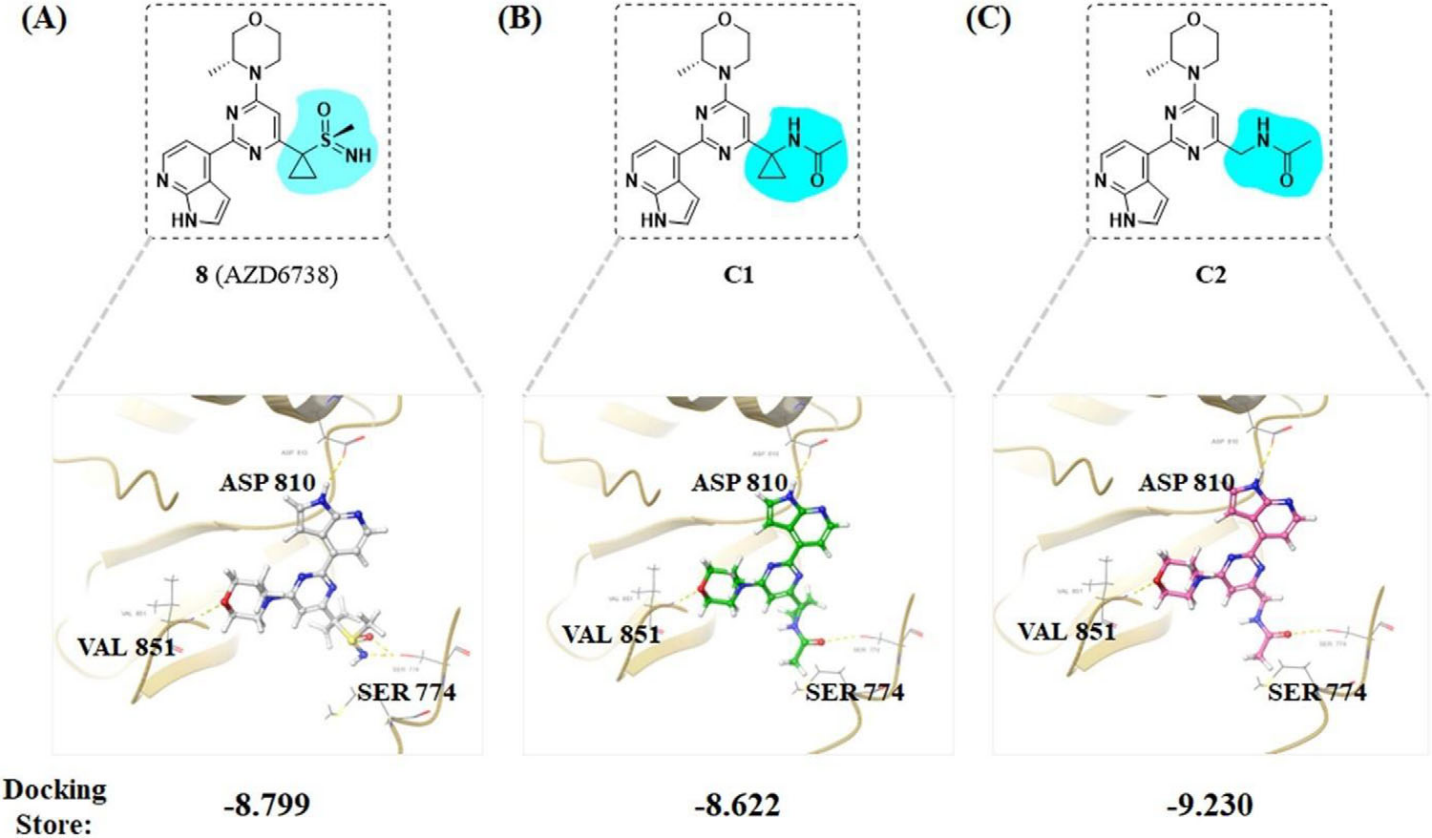
Head‐to‐head docking experiment of **8** (AZD6738) A), **C1** B), and **C2** C) with the designed PI3Kα mutant that mimics ATR (PDB: 5UL1) using Schrodinger 2024. Yellow dashed lines indicate hydrogen bond interactions.

**Scheme 3:**
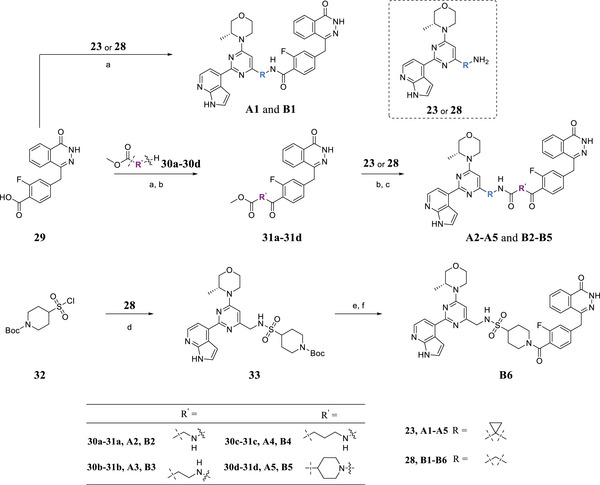
Synthesis of compounds **A1–A5** and **B1–B6**. Reagents and conditions: a) DIPEA, EDCI, HOBT, DMF, rt, 4 h, 64% ∼ 89%; b) NaOH, H_2_O, 40 °C, 2 h, 67% ∼ 86%; c) DIPEA, EDCI, HOBT, DMF, rt, 6 h, 61% ∼ 78%; d) Et_3_N, DCM, 0 °C ∼ rt; 3 h, 73%; e) HCl in 1,4‐Dioxane (4 M), MeOH, rt, 3 h, 88%; f) **29**, DIPEA, EDCI, HOBT, DMF, rt, 6 h, 70%.

**Scheme** **3** details the synthesis of compounds **A1–A5** and **B1–B6**. Simply amide formation, sulfonamide formation, ester, or Boc deprotection reactions were used to facilitate the transformation. For example, commercially available starting material **29** reacted with **23** (or **28**) to afford **A1** and **B1**, respectively. On the other hand, the amino ester intermediates bearing different R’ groups (i.e., **30a**–**30d**, selected from straight alkyl chain or with a piperidinyl group incorporated) were either commercially available or could be synthesized straightforwardly. They reacted with **29** to yield intermediates **31a**–**31d,** respectively. The methyl ester was deprotected, and the resulting acid reacted with **23** or **28** to afford **A2**–**A5** or **B2**–**B5** in good yield. For the synthesis of **B6**, commercially available **32** reacted with **28** to form sulfonamide **33**. After Boc deprotection and amide coupling reaction with **29**, **B6** was obtained in good yield.

**Scheme 4:**
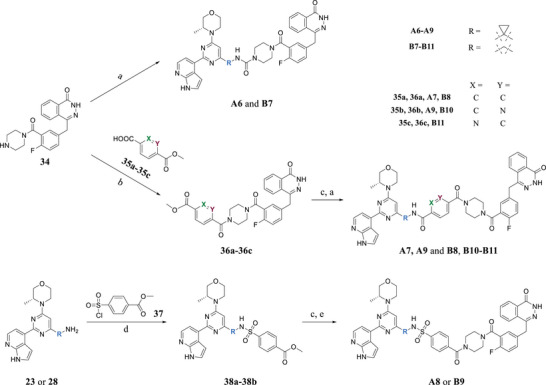
Synthesis of compounds **A6–A9** and **B7–B11**. Reagents and conditions: a) **23** or **28**, DIPEA, CDI, rt, 5 h, 85% ∼ 93%; b) DIPEA, EDCI, HOBt, DCM, rt, 6 h, 71% ∼ 84%; c) NaOH, H_2_O, MeOH, rt, 2 h, 87% ∼ 91%; d) Et_3_N, DCM, 0 °C ∼ rt; 3 h, 53% ∼ 65%; e) **34**, DIPEA, EDCI, HOBt, DCM, rt, 6 h, 71%.

Compounds **A6–A8** and **B7–B11** are analogs bearing piperazine moiety in the linker (i.e., **A6** and **B7**) or bearing both a piperazine and aromatic ring in the linker (i.e., **A7**–**A9**, **B8**–**B11**). With commercially available compounds **34, 35a–35c**, and **37** in hand, the synthesis of these target molecules is straightforward, involving 1,1′‐carbonyldiimidazole (CDI)‐mediated urea formation, amide formation reaction, sulfonamide formation reaction, and ester deprotection (**Scheme** **4**).

**Scheme 5:**
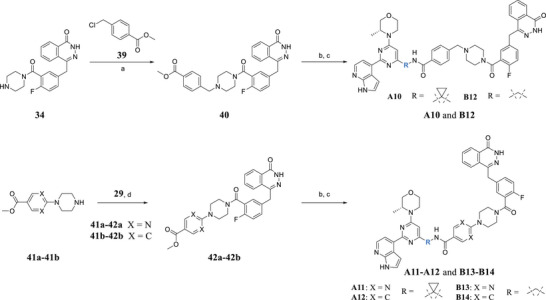
Synthesis of compounds **A10–A12** and **B12–B14**. Reagents and conditions: a) DIPEA, DMF, 60 °C, 6 h, 74%; b) NaOH, H_2_O, MeOH, rt, 2 h, 78% ∼ 85%; c) **23** or **28**, DIPEA, EDCI, HOBt, DMF, rt, 6 h, 63% ∼ 77%; d) DIPEA, EDCI, HOBt, DCM, rt, 6 h, 81% ∼ 88%.

**Scheme** **5** shows the synthesis of compounds **A10–A12** and **B12–B14**. Intermediate **34** was alkylated with methyl 4‐(chloromethyl)benzoate (**39**) to afford intermediate **40**. After ester deprotection and amide formation with **23** (or **28**), **A10** and **B12** were obtained, respectively. Similarly, commercially available starting material **29** reacted with **41a–‐41b** to produce intermediates **42a–42b,** respectively. After ester deprotection and amide formation with **23** (or **28**), **A11–A12** and **B13–B14** were obtained, respectively.

### 2.5 Inhibitory Activities of Compounds Against ATR and PARP1

The first dual inhibitor compound **A1** showed moderate inhibitory activity against ATR but much weaker activity against PARP1 (IC_50_: 215 and 1149 nM, respectively, **Table** **2**). We reasoned that the pharmacophore of PARP1 might be too close to the bulky group from the ATR pharmacophore. Thus, various straight alkyl linkers were designed (i.e., **A2–A4**). Compound **A2** has the best activity against both ATR and PARP1 compared to its counterparts **A3** and **A4**. It appears that the longer the linker, the better the inhibitory activity against PARP1 (**A4** *vs* **A3** *vs* **A2** *vs* **A1**). This provided the guidance for designing new dual inhibitors. Various linkers incorporating piperazinyl, piperdinyl, and/or phenyl/pyridinyl groups were examined. They all gave potent inhibitory activity against PARP1 (i.e., **A5**–**A12**, IC_50_ ranges from 0.56 to 19.3 nM *vs* 1.3 nM of Olaparib), but moderate to weak activity against ATR (IC_50_ ranges from 32 to 1322 nM *vs* 7.3 nM of AZD6738). Sulfonyl group in the linker appears to reduce the ATR activity (IC_50_: 43 vs 1322 nM for **A** **7** vs **A** **8**). Incorporation of nitrogen into the phenyl ring in the linker does not affect the activity (**A9** *vs* **A7**). Changing the carbonyl group in the linker to a methylene group resulted in the 4‐fold loss of ATR activity and 34‐fold loss of PARP1 activity (**A7** *vs* **A10**). Furthermore, removal of the methylene group between the phenyl ring and the piperazine ring gave similar ATR activity, but with an 8‐fold increase of PARP1 activity (**A11** *vs* **A10**). When changing the phenyl ring of **A11** to a pyrimidinyl, both ATR and PARP1 activity increased (**A12** *vs* **A11**).

**Table 2:** The structures and corresponding IC_50_ values of compounds **A1‐A12** against ATR and PARP1^a)^

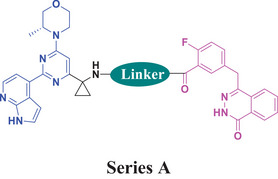			
Compd	Linker	IC_50_ (nM)	
		ATR	PARP1
**A1**	—	215 ± 22	1149 ± 43
**A2**	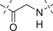	118 ± 14	12 ± 2.8
**A3**	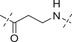	> 500	31.8 ± 2.4 % @ 20 nM^b)^
**A4**	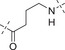	> 500	3.4
**A5**	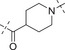	143 ± 11	1.08 ± 0.74
**A6**	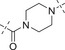	> 500	0.98 ± 0.88
**A7**	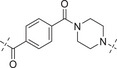	43 ± 7	0.56 ± 0.21
**A8**	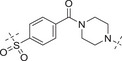	1322 ± 189	0.75 ± 0.27
**A9**	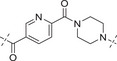	32 ± 9.3	0.60 ± 0.06
**A10**	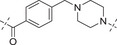	185 ± 16.3	19.3 ± 2.9
**A11**	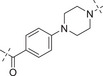	292 ± 13.9	2.3 ± 0.5
**A12**	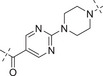	105 ± 7.2	1.7 ± 0.2
**1** (Olaparib)	—	–	1.3
**8** (AZD6738)	—	7.3	—

^a)^ Assays were performed in replicate (n = 3), IC_50_ values are shown as mean ± SD.

^b)^ Inhibitory rate at certain concentration.

**Table** **3** lists the enzymatic activities of both ATR and PARP1 for Series B compounds. In general, Series B compounds follow a similar structure‐activity relationship (SAR) trend as Series A compounds. It's interesting to find that the first compound we prepared in this series (i.e., **B1**) has better ATR activity (5‐fold increase *vs* **A1**) and better PARP1 activity (2.7‐fold increase *vs* **A1**). Head‐to‐head comparison of Series B compounds with Series A further confirmed that the cyclopropyl group in Series A does not provide additional benefit in terms of biological activity. For example, **B2**–**B5** have better ATR activity than **A2**–**A5**. Other compounds in Series B have the same trend.

**Table 3:** The structures and corresponding IC_50_ values of compounds **B1‐B14** toward the ATR and PARP1^a)^

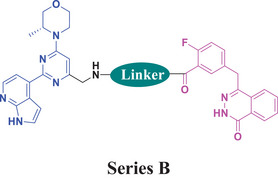			
Compd	Linker	IC_50_ (nM)	
		ATR	PARP1
**B1**	—	39.5 ± 3.7	420 ± 31
**B2**	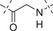	168 ± 7.8	16 ± 2.4
**B3**	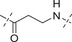	347 ± 15.7	9.5 ± 1.8
**B4**	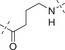	111 ± 8.1	6.2 ± 0.9
**B5**	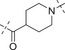	36.7 ± 4.6	1.2 ± 0.3
**B6**	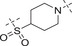	112 ± 5.3	0.46 ± 0.10
**B7**	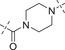	78.8 ± 6.2	0.76 ± 0.09
**B8**	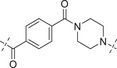	17.3 ± 2.5	0.38 ± 0.08
**B9**	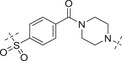	205 ± 13.1	0.64 ± 0.21
**B10**	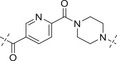	19 ± 1.2	0.61 ± 0.15
**B11**	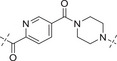	31.1 ± 4.7	1.0 ± 0.13
**B12**	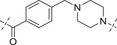	37 ± 4.2	2.0 ± 0.3
**B13**	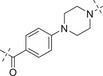	22 ± 1.7	1.3 ± 0.6
**B14**	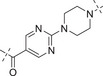	23 ± 2.1	1.2 ± 0.4
**1** (Olaparib)	—	–	1.3
**8** (AZD6738)	—	7.3	—

^a)^ Assays were performed in replicate (n = 3), IC_50_ values are shown as mean ± SD.

The fact that Series B has better ATR activity than Series A could be explained by the molecular docking experiment (Figure 6). Two truncated versions of Series A and B (i.e., **C1** and **C2**) were docked head‐to‐head with **8** to designed PI3Kα mutant protein that mimics ATR (PDB: 5UL1) using Schrodinger 2024. Key residues VAL851 and ASP810 were fixed in place without alteration. The docking score suggested that **C2** appeared to be more favorable than **C1**, presumably due to the cyclopropyl group in Series A pushing away the carbonyl group orientation, resulting in less favorable conformation with attaching a bigger side chain to the acetyl group.

Subsequently, the anti‐proliferative activity of compounds from Series A and Series B was evaluated in a cellular test using the CTG assay. Three representative TNBC cells, MDA‐MB‐231, MDA‐MB‐468, and MDA‐MB‐436, were chosen for this assay. The IC_50_ values of the different compounds against these cell lines are shown in **Table** **4**. AZD6738, Olaparib, as well as their combination (AZD6738 + Olaparib), were tested head‐to‐head with **A1**–**A12** and **B1**–**B14** against three TNBC cancer cells for proliferation. Included in the testing are two key intermediates **23** and **28**, which were used to prepare **A1**–**A12** and **B1**–**B14**. As expected, AZD6738 and Olaparib alone have moderate activity against MDA‐MB‐436 (IC_50_: 3.8 and 4.5 µM, respectively), but weak activity against MDA‐MB‐231 and MDA‐MB‐468 (IC_50_ ranging from 10.5 to 21.8 µM). Intermediates **23** and **28** are weaker than AZD6738 against TNBC cells proliferation. The combination of AZD6738 and Olaparib (1:1 ratio) substantially increases the inhibitory activity against three TNBC cells proliferation (IC_50_ ranging from 0.89 to 3.26 µM). Except **A2**, **A3**, **A10**, and **B3**, all dual inhibitors have improved anti‐proliferative activity than AZD6738 or Olaparib alone in at least one cell line. Compound **B8** stands out as the most promising dual inhibitor with excellent anti‐proliferative activity across all three TNBC cell lines (IC_50_ ranging from 0.009 to 1.89 µM). Such activities represent a 1.7‐fold increase in MDA‐MB‐231, a 5.5‐fold increase in MDA‐MB‐468, and a 100‐fold increase in MDA‐MB‐436. Compound **A7** is also interesting. It is very potent against MDA‐MB‐468 and MDA‐MB‐436, but less potent against MDA‐MB‐231 when compared with **B8**. Generally speaking, dual inhibitors with better PARP1 inhibitory activity tend to have better cellular activity. For example, **A2** and **A5** have similar ATR inhibitory activity (IC_50_: 118 and 143 nm, respectively) but different PARP1 inhibitory activity (**A5** is 10‐fold more potent than **A2**). This reflects the cellular activity where **A5** is substantially more potent than **A2**. In MDA‐MB‐231 cells, **A5** is 7.7‐fold more potent than **A2**, while in MDA‐MB‐468, **A5** is ca. 100‐fold more potent than **A2**. Other examples are **B8** and **B11**. These two compounds have similar ATR inhibitory activity, but a 3.4‐fold difference in PARP1 activity. Such enzymatic activities reflect into cellular activity differences in which **B8** is 3‐fold more potent than **B13** in MDA‐MB‐468 anti‐proliferative activity, and more than 26‐fold more potent than **B13** in MDA‐MB‐231 anti‐proliferative activity. It should be noticed that the discrepancy between enzymatic activities and cellular activities always exists. This is because other factors including solubility and permeability will affect the inhibitor entering the cells and taking into effects.

**Table 4:** Inhibitory activities of all target compounds against TNBC cells MDA‐MB‐231, MDA‐MB‐468, and MDA‐MB‐436^a)^

Compound	IC_50_ (µM)		
	MDA‐MB‐231	MDA‐MB‐468	MDA‐MB‐436
**A1**	5.26 ± 1.14	8.54 ± 1.07	2.92 ± 2.05
**A2**	28.39 ± 1.65	> 50	—
**A3**	> 50	27.33 ± 2.34	—
**A4**	> 50	3.08 ± 0.08	—
**A5**	3.63 ± 1.41	0.57 ± 0.08	0.57 ± 0.04
**A6**	4.16 ± 1.09	1.15 ± 0.01	0.59 ± 0.01
**A7**	13.38 ± 3.44	0.23 ± 0.07	0.015 ± 0.001
**A8**	> 50	0.62 ± 0.06	—
**A9**	> 50	0.75 ± 0.16	—
**A10**	> 50	> 50	—
**A11**	> 50	0.57 ± 0.02	—
**A12**	> 50	0.24 ± 0.01	—
**B1**	3.05 ± 0.76	7.88 ± 1.14	0.91 ± 0.05
**B2**	10.58 ± 0.01	8.41 ± 0.61	40.32%@5.0 µM^b)^
**B3**	14.27 ± 1.40	17.74 ± 0.53	34.90%@5.0 µM^b)^
**B4**	14.32 ± 3.91	6.83 ± 0.10	46.99%@5.0 µM^b)^
**B5**	4.48 ± 1.08	1.46 ± 0.10	1.19 ± 0.23
**B6**	3.96 ± 2.05	0.70 ± 0.07	0.78 ± 0.08
**B7**	1.60 ± 0.09	0.96 ± 0.04	1.29 ± 0.56
**B8**	1.89 ± 0.13	0.32 ± 0.01	0.009 ± 0.002
**B9**	> 50	6.88 ± 0.63	—
**B10**	> 50	1.70 ± 0.35	—
**B11**	> 50	0.48 ± 0.03	—
**B12**	> 50	2.83 ± 0.06	—
**B13**	> 50	0.99 ± 0.06	—
**B14**	> 50	0.09 ± 0.01	—
**23**	40.66 ± 1.36	> 50	20.46%@5.0 µM^b)^
**28**	> 50	40.77 ± 4.45	21.44%@5.0 µM^b)^
**1** (Olaparib)	21.87 ± 2.36	10.54 ± 0.59	4.53 ± 0.21
**8** (AZD6738)	12.57 ± 1.43	18.58 ± 1.75	3.84 ± 0.52
**1** + **8** (1:1)	3.26 ± 1.42	1.77 ± 0.37	0.89 ± 0.05

^a)^ Assays were performed in replicate (n = 3), IC_50_ values are shown as mean ± SD;

^b)^ Inhibitory rate at certain concentration.

To test whether **B8** has specificity against TNBC cells, we initiated the anti‐proliferative assay against other breast cancer cell lines such as MCF‐7 (luminal) and MDA‐MB‐453 (HER2+) (**Table** **5**). The data suggest that **B8** does not have specificity against TNBC. In addition, we also test **B8** in normal breast cell lines such as MCF‐10A. The results reveal that **B8** has minimal inhibitory activity against normal cells, similar to the effects of **1** and **8**.

**Table 5:** Inhibitory activities of representative compound **B8** against MCF‐7, MDA‐MB‐453 and MCF‐10A cells^a)^

Compound	IC_50_ (µM)		
	MCF‐7	MAD‐MB‐453	MCF‐10A
**1** (Olaparib)	> 30	> 30	14.52% @30 µM^b)^
**8** (AZD6738)	2.01 ± 0.43	1.46 ± 0.17	25.89% @30 µM^b)^
**1** + **8** (1:1)	1.64 ± 0.35	1.27 ± 0.28	3.83
**B8**	0.97 ± 0.09	1.08 ± 0.03	17.03% @30 µM^b)^

^a)^ Assays were performed in replicate (n = 3), IC_50_ values are shown as mean ± SD.

^b)^ Inhibitory rate at certain concentration.

Due to their promising activities enzymatically and cellularly, compounds **A7** and **B8** were selected for further mechanistic studies.

### 2.6 SAR Summary

**Figure 7:**
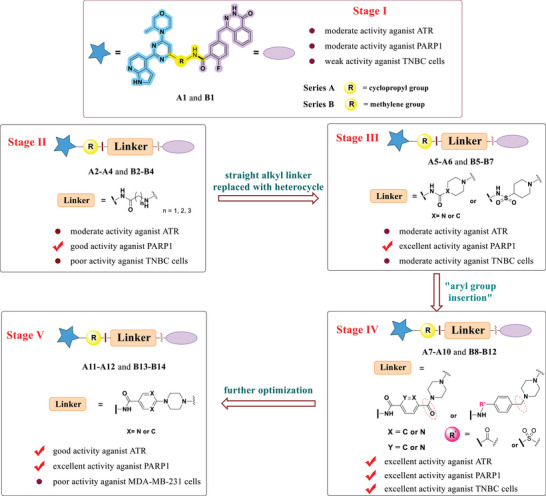
Diagram summary of structural optimization of ATR/PARP1 dual inhibitors. The processes were divided into Stages I–V.

The process of the design and structural optimization of dual inhibitors can be described as five stages (**Figure** **7**). In the initial stage (Stage I), **A1** and **B1** were designed and synthesized from key intermediates **23**, **28**, and **29**. This series of compounds exhibited moderate activity against both ATR and PARP1, as well as modest to weak anti‐proliferative activities against three different TNBC cell lines. In Stage II, a straight alkyl chain was incorporated into the linker. Although this modification enhanced the anti‐PARP1 activity to varying extents, but the effects on anti‐ATR activity were inconsistent. Notably, the cellular activities remained suboptimal for most compounds (**A2**–**A4**, **B2**–**B4**). Therefore, in Stage III, the piperazine ring or piperidine ring was used to replace the alkyl chain, ending up with compounds **A5**–**A6**, and **B5**–**B7**. Such changes significantly improved anti‐PARP1 activities, but affected the anti‐ATR activity of different compounds to different extents. Concurrently, the improvement of the anti‐TNBC proliferation activities of different compounds was also achieved. Stage IV involved the introduction of an additional aromatic ring (phenyl, pyridyl, or pyrimidinyl) into the linker at the left end, which connects to the R group, ending up with compounds **A7**–**A10** and **B8**–**B12**. Generally, the compounds in this stage have achieved potent activity against both ATR and PARP1 (with some exceptions, such as sulfonamide analog **A8**). Importantly, although not all compounds exhibited superior performance, most of them achieved excellent anti‐proliferative activities against at least one TNBC cell line. At this stage, compounds **A7** and **B8** stand out as promising leads for further evaluation. Finally, in Stage V, modification was made by removing the carbonyl group or the methylene group between piperazinyl (or piperidinyl) and aromatic ring, ending up with compounds **A11**–**A12** and **B13**–**B14** (Stage V). Such a change did not prove to be beneficial to anti‐proliferative activity against MDA‐MB‐231, despite several compounds retaining potent anti‐PARP1 inhibitory activity and reasonably good anti‐ATR activity. In summary, leads **A7** and **B8** were identified through a process of rational design and SAR optimization.

### 2.7 Pharmacology

#### 2.7.1 **B8** Induces G2/M Cell Cycle Arrest in TNBC Cells

**Figure 8:**
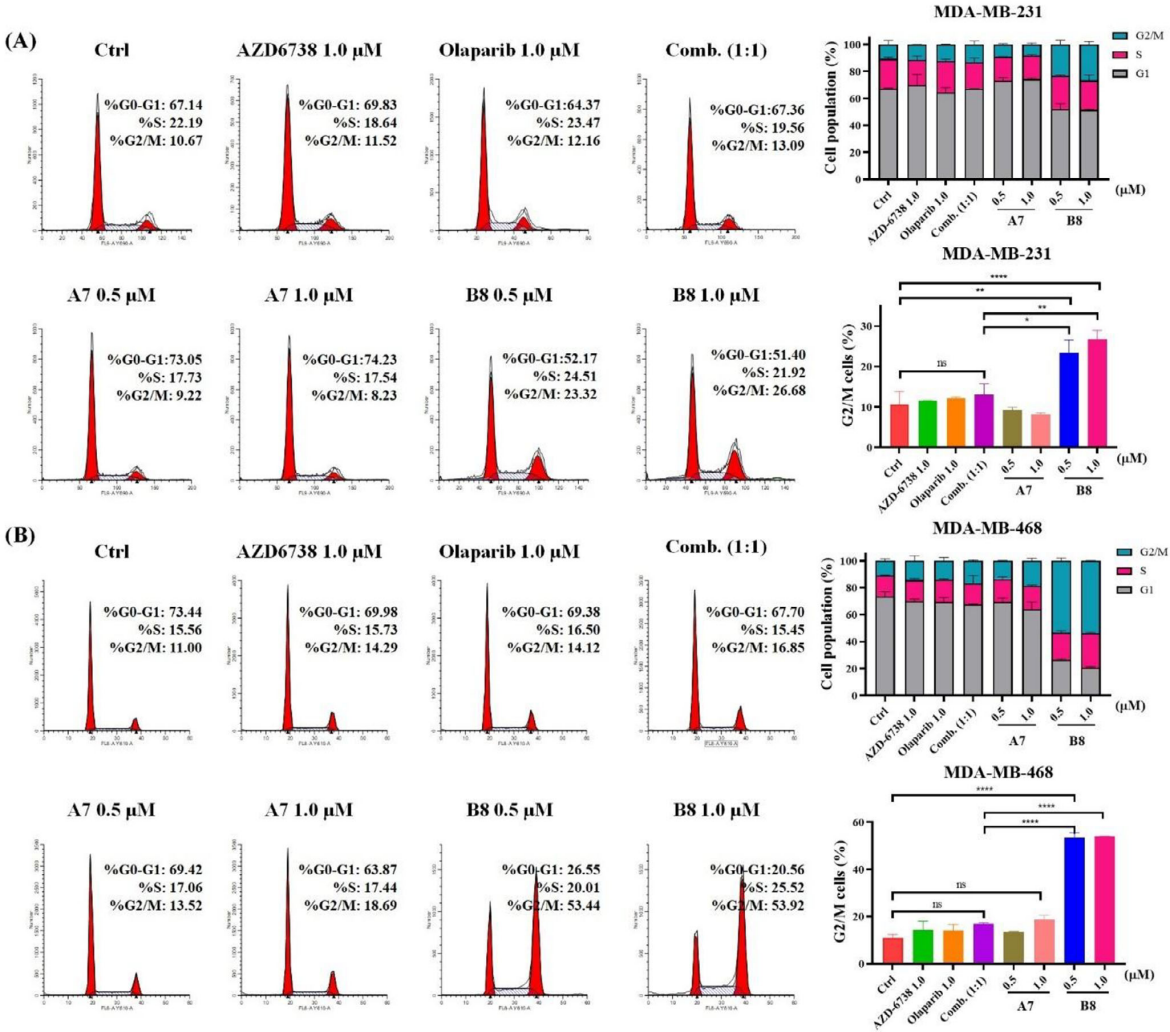
Cell cycle profile and distribution of MDA‐MB‐231 cells a) and MDA‐MB‐468 cells b) under 48 h treatment with different concentrations of compounds as indicated. Quantitative data of cell cycle distribution were calculated as the mean ± SD of three sets of experiments (n = 3). *p < 0.05, **p < 0.01, ***p < 0.001, ****p < 0.0001, ns = no significance.

Based on the results of enzyme activity and cell activity tests, representative molecules **A7** and **B8** were selected for subsequent biological characterization. Both the individual effect of Olaparib and the combination of Olaparib with AZD6738 or other ATRi lead to G2/M phase arrest in tumor cells.^[^ ^31^, ^48^ ^]^ Therefore, we investigated the effects of compounds **A7** and **B8** on the cell cycle of TNBC cells. MDA‐MB‐231 and MDA‐MB‐468 cells were treated with control compounds, including DMSO (negative control), AZD6738 (1.0 µM), Olaparib (1.0 µM), and the combination of AZD6738 and Olaparib (1.0 µM each). Additionally, MDA‐MB‐231 and MDA‐MB‐468 cells were treated with two different concentrations of **A7** and **B8** (0.5 and 1.0 µM). 48 h after treatment, flow cytometry was employed to analyze the cell cycle distribution in both TNBC cell lines (**Figure** **8**A,B). The results indicated that treatment of the two TNBC cell lines with either single inhibitor alone, AZD6738 or Olaparib, did not significantly change the cell cycle distribution. Meanwhile, the combined application of AZD6738 and Olaparib cannot lead to an increase in the proportion of cells in the G2/M phase. Regarding our novel synthesized compounds **A7** and **B8**, **A7** did not demonstrate any significant effects on the cell cycle. In contrast, both 0.5 and 1.0 µM concentrations of **B8** markedly increased the proportion of G2/M phase cells, exhibiting a significantly stronger effect than the combination of AZD6738 and Olaparib. These results demonstrated that **B8** significantly induces G2/M cell cycle arrest in TNBC cells, while **A7** does not exhibit similar activity. Their abilities in regulating the TNBC cell cycle are significantly different.

Moreover, compound **B8** markedly increased the proportion of G2/M phase cells at both concentrations (0.5 and 1.0 µM), with a significantly stronger effect than the combination of AZD6738 and Olaparib. All these pointed to the fact that **B8** has stronger abilities in regulating the TNBC cell cycle than **A7**.

Compounds **A7** and **B8** are highly similar in structure, with a subtle difference in the substitutes (cyclopropyl *vs* methylene) attaching to the C6 position of the pyrimidine group. Such a subtle difference transforms into the different synthetic scheme for the intermediates for making these two compounds. In fact, intermediate **23** (used for synthesis of **A7**) requires an additional three steps of synthesis compared to intermediate **28** (used for **B8**).

#### 2.7.2 **B8** Induces TNBC Cell Apoptosis

**Figure 9:**
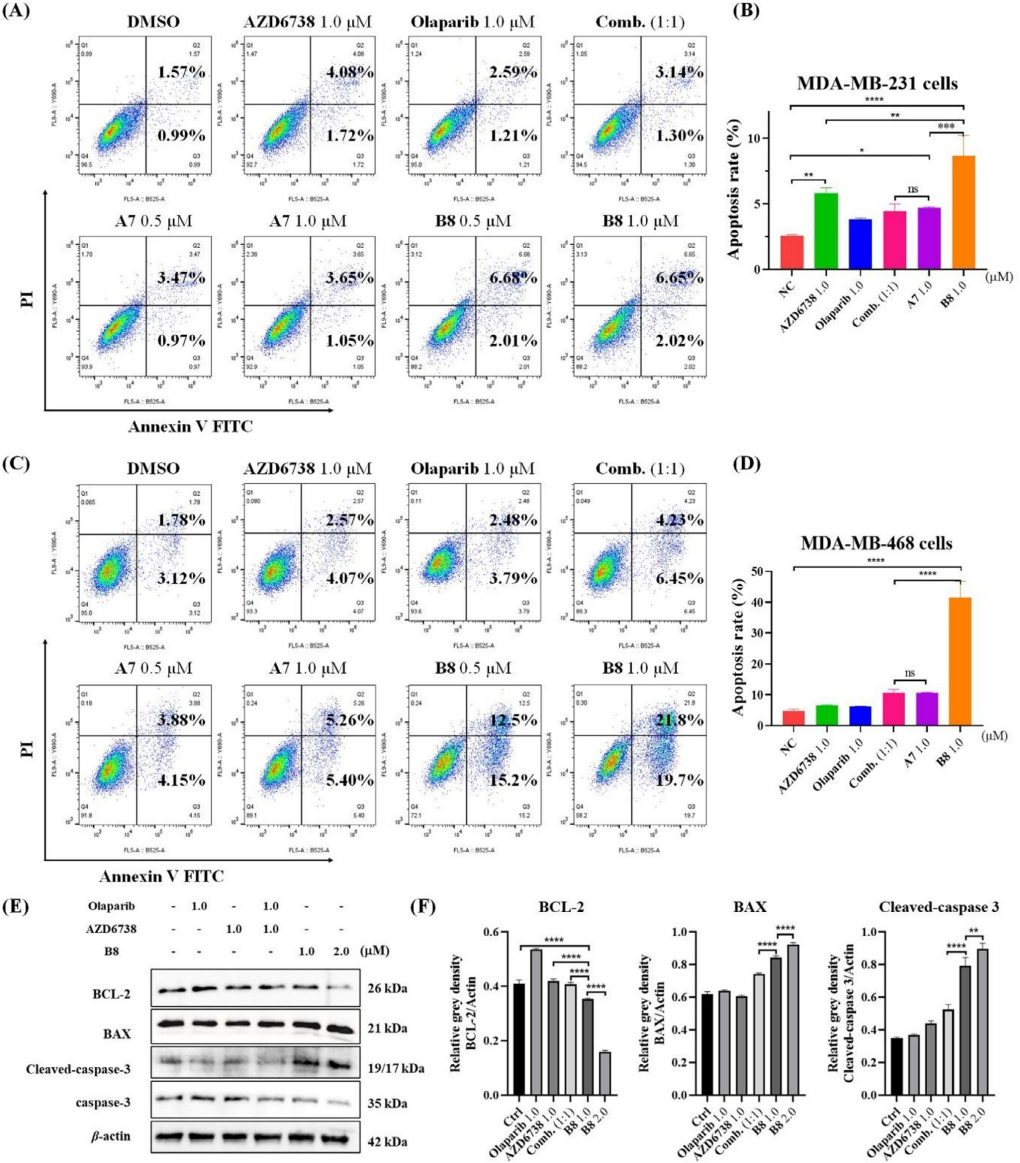
A,B) Annexin V‐FITC/PI dual staining assay to determine the apoptosis of MDA‐MB‐231 cells and MDA‐MB‐468 cells after 72 h of various treatments as indicated. C,D) Quantitative data of flow cytometry were calculated as the mean ± SD of three sets of experiments (*n* = 3). E) Western blotting of BCL‐2, BAX, Cleaved‐caspase‐3, and caspase‐3 in MDA‐MB‐468 cells exposed to different compounds for 48 h. F) Relative densitometric values of BCL‐2, BAX, Cleaved‐caspase‐3, and caspase‐3. Quantitative data were calculated as the mean ± SD of three sets of experiments (*n* = 3). Scale bar = 100 µm. *p < 0.05, **p < 0.01, ***p < 0.001, ****p < 0.0001.

Subsequently, Annexin‐V/PI staining assay was performed to determine the effects of compounds **A7** and **B8** on cell apoptosis in MDA‐MB‐231 and MDA‐MB‐468 cells. As shown in **Figure** **9**A–D, the results indicated that treatment with either AZD6738, Olaparib, or the combination of both compounds effectively induces apoptosis in the TNBC cells. Our novel synthesized compounds, **A7** and **B8**, also induce apoptosis in MDA‐MB‐231 and MDA‐MB‐468 cells. However, the ability of **A7** to induce apoptosis does not show a significant enhancement compared to the combination treatment of AZD6738 and Olaparib across both cell lines. In contrast, **B8** demonstrates a markedly stronger capacity to induce apoptosis than the combination of AZD6738 and Olaparib. It not only significantly decreased the protein expression of BCL‐2 (an anti‐apoptosis protein), but also induced more BAX and cleaved‐caspase‐3 protein expression compared to the combination of AZD6738 and Olaparib (Figure 9E). The observed enhanced apoptosis induced by **B8** suggests that simultaneous inhibition of these two pathways might lead to more effective therapeutic outcomes.

#### 2.7.3 **B8** Inhibits TNBC Cells Colony Formation, Migration, and Invasion

**Figure 10:**
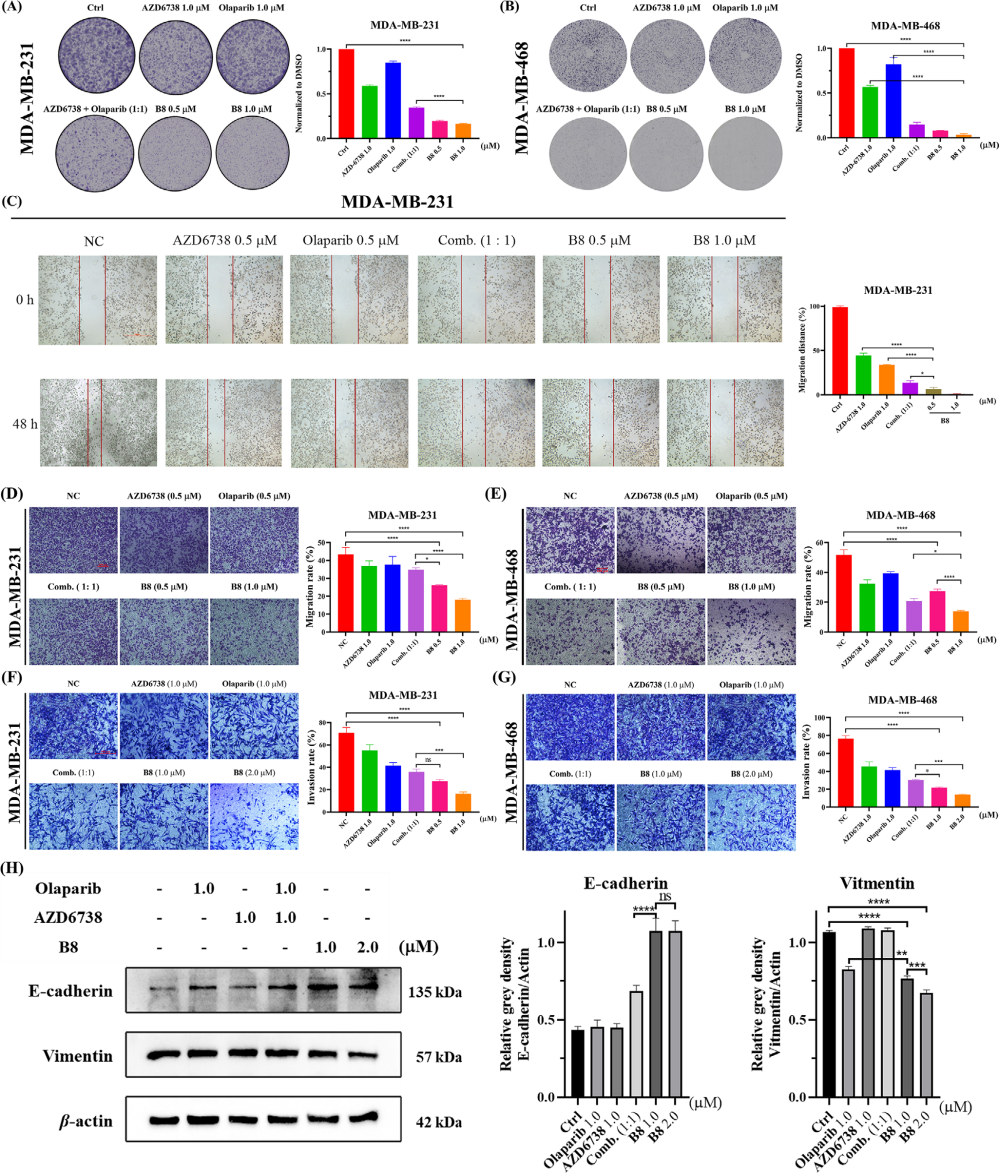
**B8** exerts anti‐tumor activity on TNBC cells in vitro. A,B) Colony formation capacity of MDA‐MB‐231 and MDA‐MB‐468 cells after treatment with compounds for 10 days. C) Representative images of wound‐healing assay and the percentage of 48 h wound‐healed distance in MDA‐MB‐231 cells. Scale bar = 500 µm. D,E) Representative images of the transwell migration assay and the percentage of 24 h areas of migrated cells per field of cells in MDA‐MB‐231 cells and MDA‐MB‐468 cells. Scale bar = 100 µm. F,G) Representative images of the transwell invasion assay and the percentage of 24 h areas of invasion cells per field of cells in MDA‐MB‐231 cells and MDA‐MB‐468 cells. H) Western blotting and relative densitometric values of Vimentin and E‐cadherin in MDA‐MB‐468 cells exposed to different compounds as indicated for 48 h. Quantitative data were calculated as the mean ± SD of three sets of experiments (*n* = 3). Scale bar = 100 µm. *p < 0.05, **p < 0.01, ***p < 0.001, ****p < 0.0001, ns: no significance.

Given that **B8** demonstrated significantly superior regulation of G2/M cell cycle arrest and apoptosis compared to the combination of Olaparib and AZD6738, it was chosen for further investigation. The colony formation assay is a conventional method for determining the effect of compounds on the proliferative capacity of cells. We first evaluated the effect of **B8** on the clonogenicity of MDA‐MB‐231 and MDA‐MB‐468 cells using a colony formation assay. The results indicated that, at a concentration of 1.0 µM, **B8** exhibited a significantly greater inhibitory effect on the colony formation rate in both MDA‐MB‐231 (**Figure** **10**A) and MDA‐MB‐468 (Figure 10B) cells compared to either treatment of AZD6738 or Olaparib administered alone, or the combination treatment of AZD6738 and Olaparib. Moreover, we detected the anti‐proliferation effect of **B8** on MDA‐MB‐231 and MDA‐MB‐468 cells with 24, 48, and 72 h treatment, **B8** exerted anti‐proliferation activity at different time points (24, 48, 72 h) (Figure S2). Subsequently, wound‐healing and transwell assays were performed to determine the effects of **B8** on migration (Figure 10C–E) and invasion (Figure 10F–G) of TNBC cells. **B8** demonstrated superior inhibitory effect on the migratory capacity of MDA‐MB‐231 cells compared to AZD6738 and Olaparib alone or their combination. Moreover, it also exhibited superior inhibitory effects on the invasive capacity of both MDA‐MB‐231 and MDA‐MB‐468 cells compared to AZD6738 and Olaparib alone or their combination (Figure 10F,G). Furthermore, the levels of EMT‐related protein, E‐cadherin and Vimentin were determined by using Western blot analysis (Figure 10H). Compared to the combination group, MDA‐MB‐468 cells in the **B8** treatment group exhibited a significant increase in the expression of the epithelial cell marker E‐cadherin and a decrease in the expression of the mesenchymal cell marker Vimentin. Taken together, these results indicated that **B8** interfered the EMT (Epithelial‐mesenchymal transition) of TNBC cells. Compared to the combination of Olaparib and AZD6738, **B8** demonstrated a stronger anti‐cancer activity.

#### 2.7.4 **B8** Induced DNA Damage in TNBC Cells

**Figure 11:**
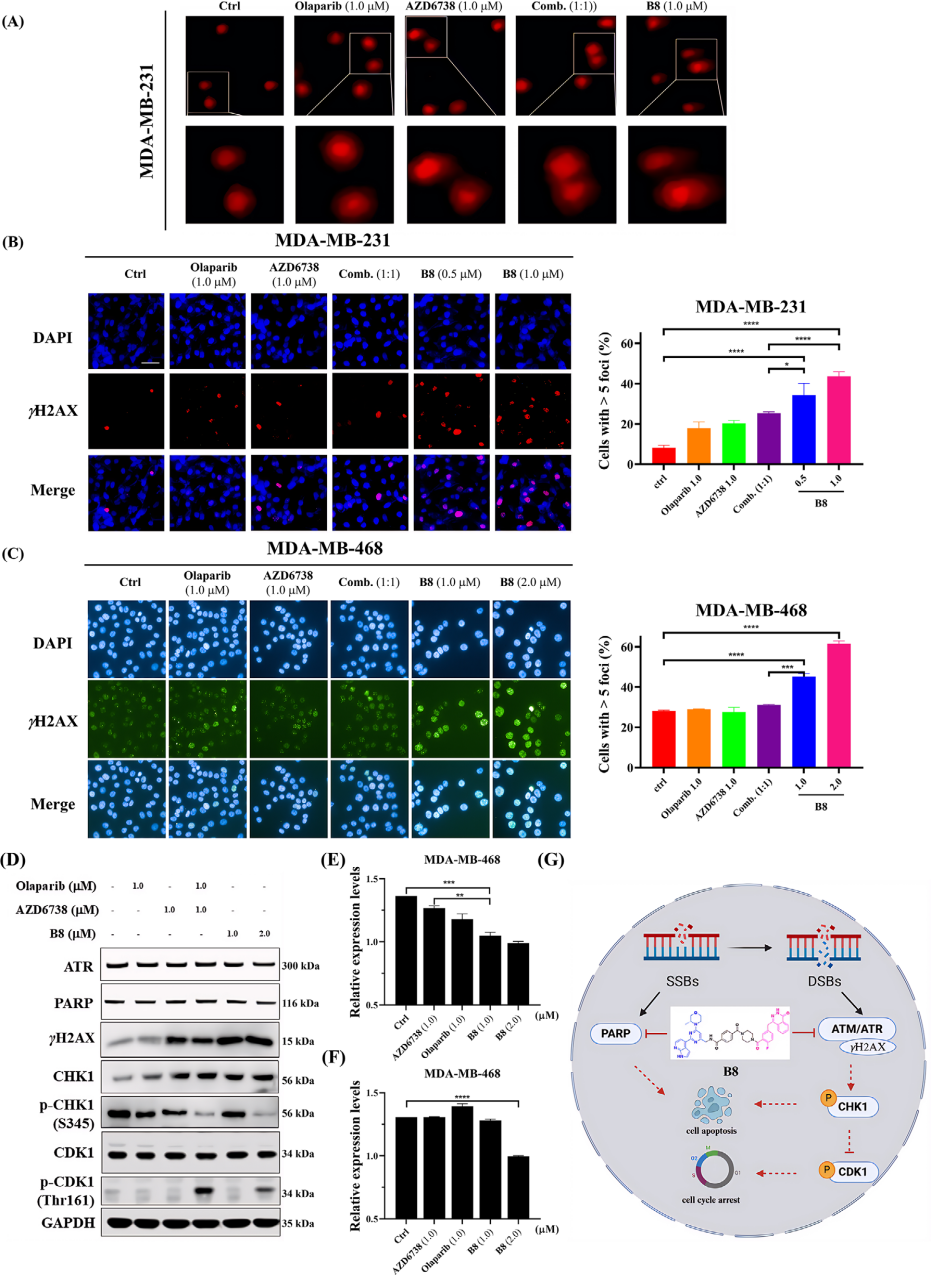
**B8** can inhibit DSB repair. A) Representative images of the comet assay for the measurement of DNA damage in MDA‐MB‐231 cells under treatment with the indicated compounds for 24 h. Scale bar = 50 µm. B,C) Representative images of immunofluorescence staining of *γ*H2AX foci in MDA‐MB‐231 B) and MDA‐MB‐468 C) cells treated with compounds as indicated for 48 h. Cell nuclei were stained with 4′,6‐diamidino‐2‐phenylindole (DAPI). D) Western blotting of ATR, PARP1, *γ*H2AX, p‐CHK1/CHK1, and p‐CDK1/CDK1 in MDA‐MB‐468 cells exposed to different compounds as indicated for 48 h. E,F) Statistical analysis of ATR E) and PARP1 F) protein levels. G) Mechanism of action of **B8** in the treatment of TNBC. The data are shown as the mean ± SD of three independent experiments. Scale bar = 50 µm. *p < 0.05, **p < 0.01, ***p < 0.001, ****p < 0.0001.

Next, we aimed to elucidate the mechanism by which **B8** induces cell death in TNBC cells. Since both PARPi and ATRi exert anti‐tumor activities by disrupting the normal DNA damage repair process, we investigated the DNA damage induction effect of **B8** in TNBC cells by using a comet assay to determine DNA damage in MDA‐MB‐231 cells following treatment with **B8** and the control agents. As shown in **Figure** **11**A, compound **B8** induced significant DNA damage in MDA‐MB‐231 cells. Notably, compared to AZD6738, Olaparib, and their combination treatment, **B8** resulted in a markedly increased tail intensity, indicating a greater accumulation of DNA breaks. This finding suggests that the inhibitory effect of **B8** on TNBC may be mediated through the induction of DNA damage. To support the results of the comet assay, immunofluorescence staining analysis of the nuclear foci of *γ*H2AX protein, a biological marker for DSBs, was conducted. As shown in Figure 11B,C, compared to Olaparib, AZD6738, and their combination, compound **B8** significantly increased the formation of *γ*H2AX foci in MDA‐MB‐231 and MDA‐MB‐468 cells, which indicated **B8** impaired DSBs repair efficiency in TNBC cells.

We further examined the impact of **B8** on the DDR downstream signaling pathway after the inhibition of ATR and PARP1. MDA‐MB‐468 cells were treated with varying concentrations of **B8** (1.0 and 2.0 µM) and control agents for 48 h. Western blot analysis was conducted to evaluate the protein levels of ATR, PARP1. As shown in Figure 11D, treatment with **B8** led to a reduction in the protein levels of both ATR and PARP1 compared to either Olaparib or AZD6738 alone treatment or in combination. Furthermore, the increase in the DSB marker γH2AX in **B8**‐treated cells was significantly increased than under the combination treatment of Olaparib and AZD6738. We also assessed the phosphorylation levels of CHK1, a key downstream molecule of ATR within the DDR signaling pathway, as well as the phosphorylation of the cell cycle regulatory protein CDK1. The results demonstrated that **B8** significantly decreased CHK1 Ser345 phosphorylation and enhanced CDK1 Tyr15 phosphorylation. Given that CHK1 activation can inhibit CDC25A and exert an inhibitory effect on CDK1 phosphorylation,^[^ ^49^ ^]^ our findings imply that **B8** suppresses CHK1 phosphorylation through ATR inhibition, thereby increasing CDK1 phosphorylation levels and consequently regulating the cell cycle. Taken together, our findings indicate that **B8** exhibits greater biological efficacy against DDR compared to the combination treatment of Olaparib and AZD6738, thereby elucidating the anti‐TNBC pharmacological mechanisms of **B8** (Figure 11G).

### 2.8 Kinase Selectivity Profile of **B8**

Phosphatidylinositol 3‐kinase‐related kinases (PIKKs) are a family of Ser/Thr‐protein kinases with sequence similarity to phosphatidylinositol‐3 kinases (PI3Ks).^[^ ^50^ ^]^ In order to test the ATR selectivity of compound **B8** over other PIKK family members, four representative PIKK family members were chosen: DNA‐PK, PI3Kα, ATM, and mTOR (**Table** **6**). Compound **8** (AZD6738) exhibits properties as an ATR‐selective kinase inhibitor with a selectivity exceeding 300 times over DNA‐PK, PI3Kα, ATM, and mTOR. Similarly, **B8** demonstrated comparable ATR selectivity profiles to those of AZD6738, although its ATR activity was slightly less potent than AZD6738.

**Table 6:** ATR selectivity profiles of **B8** ^a)^

Compd	IC_50_ (nM)				
	ATR	DNA‐PK	PI3Kα	ATM	mTOR
**B8**	17.3 ± 2.5	> 5000	> 2500	> 5000	10.3% @2500 nM^b)^
**8** (AZD6738)	7.59 ± 0.34	2310 ± 72.4	> 2500	> 2500	1341.9 ± 13.5

^a)^ Assays were performed in replicate (n = 3), IC_50_ values are shown as mean ± SD.

^b)^ Inhibitory rate at certain concentration.

To determine whether compound **B8** has potential off‐target effects, we tested compound **B8** in several representative PARP families. Olaparib was used as the reference for a head‐to‐head comparison. Compound **B8** exhibited stronger PARP1 inhibitory activity than Olaparib (IC_50_: 0.38 vs. 1.0 nm, **Table** **7**), but weaker PARP2 inhibitory activity (IC_50_: 5.1 vs. 0.6 nm). Its PARP1 selectivities over PARP2 and PARP7 are indeed better than Olaparib (13.4‐fold vs. 0.6‐fold; 75‐fold vs. 26.5‐fold). In addition, **B8** also showed moderate inhibitory activity against PARP7 (28.5 nm), while its inhibitory activity against PARP5A and PARP5B was relatively weak. The results show that compound **B8** has a strong kinase selectivity effect on both ATR and PARP1.

**Table 7:** PARP1 selectivity profiles of **B8** and Olaparib^a)^

Compd	IC_50_ (nM)				
	PARP1	PARP2 (selectivity over PARP1, fold)^b)^	PARP5A (selectivity over PARP1, fold)^b)^	PARP5B (selectivity over PARP1, fold)^b)^	PARP7 (selectivity over PARP1, fold)^b)^
**B8**	0.38 ± 0.08	5.1 (13.4)	116 (305)	240 (631.6)	28.5 ± 1.3 (75)
**1** (Olaparib)	1.00 ± 0.30	0.60 ± 0.12 (0.6)	585.00 ± 66.5 (585)	740.5 ± 108.19 (740.5)	26.50 ± 1.50 (26.5)

^a)^ Assays were performed in replicate (n = 3), IC_50_ values are shown as mean ± SD.

^b)^ Parentheses indicate the fold of PARP1 selectivity over other PARP members.

### 2.9 Molecular Docking of **B8** with PI3Kα Mutant and PARP1

**Figure 12:**
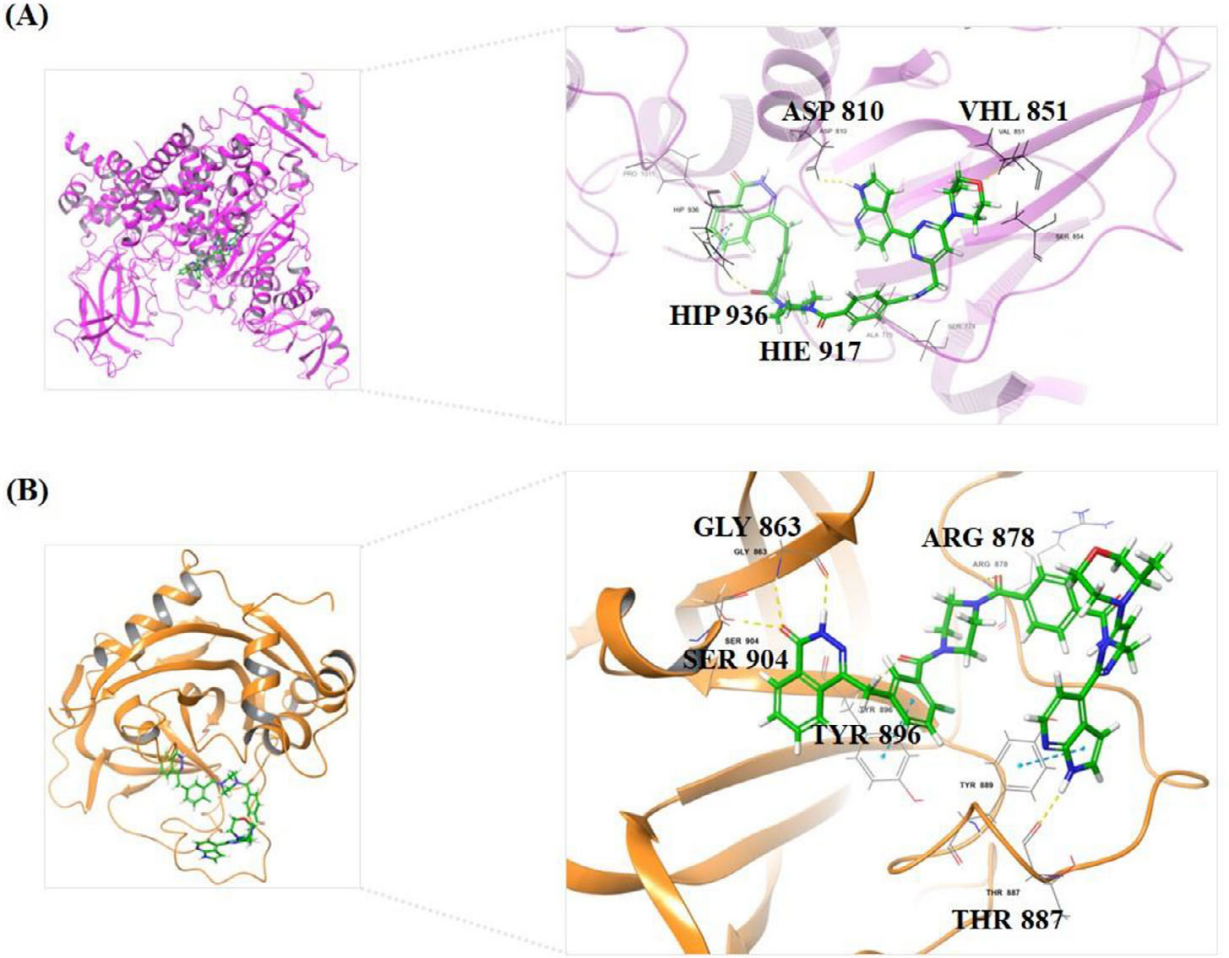
Molecular docking and binding modes of compound **B8** with ATR and PARP1, respectively. A) Compound **B8** docking using PI3Kα mutant (a mimetic ATR protein, PDB ID: 5UL1). The close view of the key residues from the protein with **B8** was presented in the right diagram. B) compound **B8** docking with PARP1 co‐crystal structures (PDB ID: 5DS3). The close view of the key residues from the protein with **B8** was presented in the right diagram. Only the surrounding amino acid residues with close proximity to **B8** are shown for clarity. See Supporting Information for the 2D view of the interactions.

To gain a deeper insight into the binding modes of compound **B8** with ATR and PARP1, we performed molecular docking studies by docking small molecules with their corresponding proteins, specifically the co‐crystal structure with PI3Kα mutant (a mimetic protein for ATR: PDB ID: 5UL1) and PARP1 (a PARP1 protein complexed with Olaparib, PDB ID: 5DS3), (**Figure** **12**). Compound **B8** binds with mimetic ATR protein and PARP1 protein, mainly through formed hydrogen bond interactions with amino acid residues on the surface of the proteins. When compound **B8** was docked with PI3Kα mutant (Figure 12A), ATR pharmacophore from **B8** has several key hydrogen bond interactions with the protein: i) morpholine oxygen interacts with VAL851; ii) NH from 1*H*‐pyrrolo[2,3‐*b*]pyridine interacts with ASP810; iii) two carbonyl groups attaching to piperazine interact with HIE917 (HIE means Histidine with hydrogen on the epsilon nitrogen) and HIP936 (HIP means Histidine with hydrogens on both nitrogens; this is positively charged), respectively. It's interesting that the linker and the phthalazin‐1(2*H*)‐one (PARP pharmacophore) also can contribute to the interactions with protein residues. On the other hand, **B8** has several key interactions with PARP1 protein (Figure 12B): i) phthalazin‐1(2*H*)‐one interacts with SER904, GLY863, and TYR907; ii) two carbonyl groups attaching to piperazine ring interact with ARG878 and TYR896 respectively; iii) 1*H*‐pyrrolo[2,3‐*b*]pyridine motif interacts with THR887 and TYR889, respectively. It's also important to note that the linker and 4‐(2‐(1*H*‐pyrrolo[2,3‐*b*]pyridin‐4‐yl)pyrimidin‐4‐yl)morpholine moiety (ATR pharmacophore) also contributes to the interactions with PARP1 protein residues. These additional interactions promote tighter binding of **B8** to both ATR and PARP1 proteins, favor lower energy conformations, thus improving the in vitro activity (ATR and PARP1 inhibitory activity, IC_50_: 17.3, and 0.38 nM, respectively). In fact, **B8** is more potent than Olaparib in terms of PARP1 activity.

### 2.10 In vivo Studies of Compound **B8** in Xenografted Mice Model Experiments and Preliminary Safety Evaluation of **B8**

**Figure 13:**
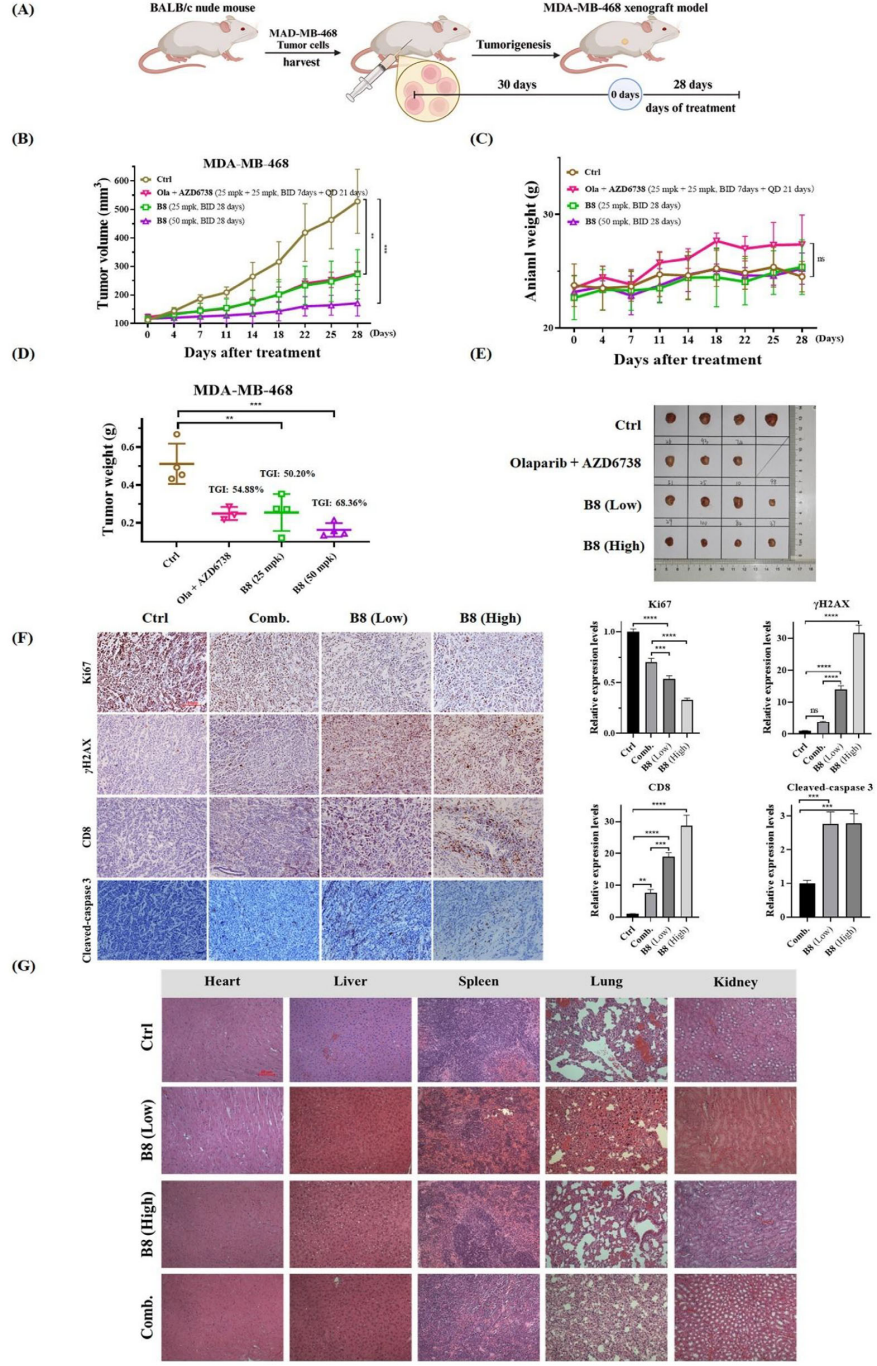
Compound **B8** exerts anti‐tumor efficacy in the MDA‐MB‐468 xenografted mice model. A) Schematic procedure for the in vivo animal experiment. B) Tumor volumes of each group of mice were measured at the indicated time after treatments. C) Animal weights of each group of mice were measured at the indicated time after treatments. D) Tumor weights in each group mice following treatment with different compounds for 28 days. E) Representative image of tumors. F) IHC staining of Ki67, *γ*H2AX, CD8, and cleaved‐caspase 3 levels in MDA‐MB‐468 xenograft tumors (scale bar = 100 µm). G) H&E staining of heart, liver, kidney, lung, and spleen tissues of each group xenograft mice (scale bar = 50 µm). *p < 0.05, **p < 0.01, ***p < 0.001, ****p < 0.0001, ns: no significance.

Encouraged by the excellent potency in vitro, **B8** was further progressed into in vivo antitumor activity studies. First, MDA‐MB‐468 cells (1 × 10^7^) were implanted in the right flanks subcutaneously in female nude mice (**Figure** **13**A). When the implanted tumor reached a volume of 110 mm^3^, the animals were randomly divided into four groups: vehicle, combination of Olaparib and AZD6738, **B8** low dose group (25 mg/kg), and **B8** high dose group (50 mg/kg). Each group contained 4 animals. Compound **B8** was intraperitoneally (I.P.) administered at doses of 25 and 50 mg/kg once daily. The combination of AZD6738 and Olaparib, was intraperitoneally administered at a dose of 25 mg/kg once daily. Compound **B8** can significantly suppress the growth of MDA‐MB‐468 xenografted mice (Figure 13B–E). The tumor growth inhibition rates (TGI) of the two **B8** administration groups were 50.20% and 68.36%, which were higher than that in the combination of AZD6738 and Olaparib group (54.88%). Tumor weight and volume in the **B8**‐treated mice were significantly lower than control groups. In addition, administration of **B8** had no significant effect on body weight (Figure 13C). The expression level of Ki67, a nuclear antigen widely expressed in proliferating cells, is strongly correlated with tumor proliferation and growth. Immunohistochemical analysis showed that administration of **B8** decreased Ki67 expression and increased *γ*H2AX and cleaved‐caspase 3 expression (Figure 13F). In addition, **B8** also increased the levels of CD8 expression in tumor tissues of mice treated with **B8** (Figure 13F). This observation suggests that **B8** may have the potential to enhance anti‐tumor immunity. Taken together, these results indicated that **B8** exerts promising antitumor efficacy in vivo.

Since the administration of **B8** at 50 mg/kg did not result in any significant changes in the body weight of nude mice (Figure 13C), we conducted H&E staining analysis to assess whether **B8** had potential toxicity in various organs of the mice. The results indicate that **B8** did not cause significant changes in organ structure or cellular morphology of heart, liver, spleen, lungs, and kidneys after 28 days of continuous administration (Figure 13G). All of these results collectively indicate that **B8** possesses a favorable safety profile in vivo. On contrast, the combination group (Olaparib + AZD6738), one animal died on Day 7, which indicated potential toxicity.

## 3 Conclusion 

TNBC, defined by the absence of ER, PR, and HER2 amplification, represents the most aggressive breast cancer subtype with limited treatment options and poor prognosis. Given its heavy reliance on DNA damage repair (DDR) pathways, particularly ATR and PARP1, dual inhibition of these targets presents a promising therapeutic strategy. Preclinical studies have demonstrated that combining ATRi and PARPi enhances cytotoxicity not only in HR‐deficient tumors but also in HR‐proficient cancers with inherent replication stress, such as oncogene‐driven malignancies.^[^ ^37^, ^51^, ^52^, ^53^ ^]^ This synergy arises from simultaneously disrupting single‐strand break (SSB) repair (via PARP inhibition) and replication stress management (via ATR inhibition), creating a broader therapeutic window. Several clinical trials evaluating ATRi/PARPi combinations (e.g., AZD6738 + olaparib) have shown promising activity in ovarian and breast cancers, reinforcing the translational potential of this approach.^[^ ^54^, ^55^, ^56^ ^]^

In this study, we adopted an innovative strategy by developing a single‐molecule dual inhibitor of ATR and PARP1, circumventing the pharmacokinetic and toxicity challenges associated with combination therapy. Through a rational pharmacophore‐merging approach, we designed and optimized a novel series of ATR/PARP1 dual inhibitors, with **B8** emerging as the lead candidate. **B8** demonstrated potent enzymatic inhibition of both ATR and PARP1, along with superior anti‐proliferative effects against BRCA‐proficient TNBC cells compared to individual inhibitors or their combination. Mechanistically, **B8** disrupted the ATR/ATM‐CHK1‐CDK1 signaling axis, leading to DNA damage accumulation, G2/M cell cycle arrest, and apoptosis. Importantly, in an MDA‐MB‐468 xenograft model, **B8** exhibited significant tumor growth inhibition without detectable organ toxicity, suggesting a favorable therapeutic window.

Despite these encouraging findings, challenges remain. Although **B8** avoids the overlapping myelosuppression commonly observed with sequential ATRi/PARPi administration (e.g., hematological toxicities in AZD6738 + Olaparib trials), its poor oral bioavailability necessitates I.P. delivery in preclinical models, limiting clinical translatability. Future efforts should focus on structural optimization to improve pharmacokinetic properties while maintaining dual‐target potency. Additionally, patient stratification based on biomarkers (e.g., ATM loss, BRCAness signatures) will be crucial to identify responsive TNBC subsets, as unselected populations may not derive clinical benefit.

In conclusion, our work highlights the therapeutic potential of ATR/PARP1 dual inhibitors, particularly for TNBC, and provides a foundation for further development of this novel drug class. By integrating dual DDR blockade into a single molecule, **B8** offers a promising alternative to conventional combination therapies, with the potential to enhance efficacy while minimizing toxicity. Future studies should explore broader applications in other DDR‐dependent cancers and optimize drug‐like properties to advance this strategy toward clinical evaluation.

## 4 Experimental Section

### General Methods of Chemistry

All of the chemical materials were purchased from commercial suppliers. The melting points of the compounds were determined using Büchi B‐540 capillary melting point instrument. NMR spectra were recorded on a Bruker instrument at 500 MHz for ^1^H NMR and 126 MHz for ^13^C NMR, using CDCl_3_ or DMSO‐*d*_6_ as the deuterated solvent. Chemical shifts (*δ*) were reported in parts per million (ppm) relative to residual solvent as an internal reference. HPLC purity of all final compounds was recorded in a Shimadzu LC‐2030 Plus Liquid Chromatograph with the following parameters: Column: Phenomenex Luna 5 µm C18(2) 100 Å 250 × 4.6 mm; mobile phase A: H_2_O, mobile phase B: MeOH; Flow rate 0.5 mL min^−1^; Detector: 254 nm; Injection volume: 10 µL; method: 70% B for 0.5 min, 70% B to 100% B in 5.5 min gradient, 100% B for another 10 min. stop at 16.01 min. Low‐resolution mass spectra were recorded using Agilent 1260 Infinity II/1625. High‐resolution mass spectra (HRMS) were measured on an Agilent 6530 Q‐TOF instrument.

Due to the space limit, the synthesis and characterization of all intermediates are presented in the Supporting Information.

### Synthesis of Compound **A1**

(*R*)‐2‐fluoro‐*N*‐(1‐(6‐(3‐methylmorpholino)‐2‐(1*H*‐pyrrolo[2,3‐*b*]pyridin‐4‐yl)pyrimidin‐4‐yl)cyclopropyl)‐5‐((4‐oxo‐3,4‐dihydrophthalazin‐1‐yl)methyl)benzamide (**A1**). To a solution of intermediate **23** (50 mg, 0.14 mmol) in DMF (3.0 mL) were added **29** (50.71 mg, 0.17 mmol), DIPEA (54.18 mg, 0.42 mmol), HOBT (24.32 mg, 0.18 mmol), and EDCI (69.01 mg, 0.36 mmol). The resulting solution was stirred at room temperature for 3 h. The reaction was monitored by TLC. Upon completion, the mixture was quenched with cool water (30 mL) at room temperature and extracted with EtOAc (30 mL × 3). The combined organic layers were washed with water and brine, and dried over Na_2_SO_4_. The drying agent was filtered off. The filtrate was concentrated under reduced pressure, and the residue was purified via flash column chromatography (CH_2_Cl_2_/MeOH 97:3, *v/v*) to give compound **A1** as a white solid (yield 78%). m.p. 231.2–233.4 °C. Purity: 98.98% by analytical HPLC. ^1^H NMR (500 MHz, DMSO‐*d*_*6*_) *δ* 12.60 (s, 1H), 11.75 (s, 1H), 8.89 (td, *J* = 5.9, 2.8 Hz, 1H), 8.38–8.22 (m, 2H), 8.04–7.94 (m, 2H), 7.84 (dtd, *J* = 20.7, 7.3, 1.4 Hz, 2H), 7.69 (dd, *J* = 6.9, 2.4 Hz, 1H), 7.58–7.48 (m, 2H), 7.37–7.23 (m, 2H), 6.70 (s, 1H), 4.53 (dd, *J* = 5.9, 2.5 Hz, 3H), 4.35 (s, 2H), 4.11 (d, *J* = 13.3 Hz, 1H), 4.00 (dd, *J* = 11.4, 3.8 Hz, 1H), 3.79 (d, *J* = 11.4 Hz, 1H), 3.66 (dd, *J* = 11.5, 3.2 Hz, 1H), 3.51 (td, *J* = 11.8, 3.1 Hz, 1H), 3.25 (td, *J* = 12.8, 3.9 Hz, 1H), 1.24 (d, *J* = 6.8 Hz, 3H). ^13^C NMR (126 MHz, DMSO‐*d*_*6*_) *δ* 169.37, 165.11, 162.86, 162.48, 159.88, 159.58, 157.60, 150.26, 145.35, 142.88, 137.81, 134.59 (d, *J* = 3.4 Hz), 133.95, 132.86 (d, *J* = 8.4 Hz), 132.02, 130.62 (d, *J* = 2.4 Hz), 129.57, 128.40, 127.44, 126.58, 125.94, 124.98, 124.86, 117.72, 116.76, 116.58, 114.28, 101.43, 98.43, 70.73, 66.50, 60.23, 46.66, 39.04, 37.16 (d, *J* = 4.7 Hz), 18.93 (d, *J* = 7.6 Hz), 13.74. HR‐MS (ESI) Calcd. for C_35_H_31_FN_8_O_3_ [M + H] ^+^: *m/z* 631.2576, Found 631.2573.

### General Synthetic Procedure for Compounds **A2**–**A5**

To a solution of intermediates **31** (0.5 mmol) in MeOH (8 mL) was added aqueous NaOH solution (1.5 mmol NaOH in 2 mL of H_2_O). The mixture was stirred at room temperature for 2 h. The reaction was monitored by TLC. Upon completion, the mixture was adjusted to pH 2 ∼ 3 using aqueous HCl solution (1 N). The mixture was diluted with cool water (50 mL) and extracted with EtOAc (50 mL × 2). The combined organic layers were washed with water, brine, and dried over Na_2_SO_4_. The drying agent was filtered off. The filtrate was concentrated under reduced pressure to afford the corresponding acids as a white solid, which was used directly in the next stage without further purification.

To a solution of above acids (0.45 mmol, crude material from previous step) and DIPEA (1.35 mmol) in DMF (5.0 mL) were added **23** (0.54 mmol), HOBT (0.59 mmol), and EDCI (1.17 mmol). The resulting solution was stirred at room temperature for 3 h. The reaction was monitored by TLC. Upon completion, the mixture was quenched with cool water (50 mL) and extracted with EtOAc (50 mL × 3). The combined organic layers were washed with water and brine, and dried over Na_2_SO_4_. The drying agent was filtered off. The filtrate was concentrated under reduced pressure, and the residue was purified via flash column chromatography (CH_2_Cl_2_/MeOH 93:7, *v/v*) to give the corresponding target compounds **A2–A5** as a white solid.

*(R)*‐*2*‐*fluoro*‐*N*‐*(2*‐*((1*‐*(6*‐*(3*‐*methylmorpholino)*‐*2*‐*(1H*‐*pyrrolo* *[2,3*‐*b]pyridin*‐*4*‐*yl)pyrimi* *din*‐*4*‐*yl)cyclopropyl)amino)*‐*2*‐*oxoethyl)*‐*5*‐*((4*‐*oxo*‐*3,4*‐*dihydrophthalazin*‐*1*‐*yl)methyl)benza* *mide* *(* ***A2*** *)*. White solid, yield 71%. m.p. 249.3‐252.1 °C. Purity: 96.09% by analytical HPLC.^1^H NMR (500 MHz, *DMSO* *‐* *d*_*6*_) *δ* 12.58 (s, 1H), 11.76 (s, 1H), 8.69 (s, 1H), 8.34 – 8.21 (m, 3H), 7.96 (d, *J* = 8.0 Hz, 1H), 7.85 (dtd, *J* = 30.4, 7.3, 1.3 Hz, 2H), 7.71 (dd, *J* = 7.0, 2.4 Hz, 1H), 7.63 (d, *J* = 5.0 Hz, 1H), 7.57 – 7.45 (m, 2H), 7.24 (dd, *J* = 10.8, 8.4 Hz, 1H), 7.08 (s, 1H), 7.01 (dd, *J* = 3.4, 1.9 Hz, 1H), 4.52 (s, 1H), 4.34 (s, 2H), 4.16 (d, *J* = 13.3 Hz, 1H), 4.01 (d, *J* = 5.5 Hz, 2H), 3.92 (dd, *J* = 11.5, 3.8 Hz, 1H), 3.71 (d, *J* = 11.4 Hz, 1H), 3.59 (dd, *J* = 11.5, 3.1 Hz, 1H), 3.44 (td, *J* = 11.8, 3.1 Hz, 1H), 3.14 (td, *J* = 12.8, 3.7 Hz, 1H), 1.66 – 1.50 (m, 2H), 1.19 (d, *J* = 6.5 Hz, 5H). ^13^C NMR (126 MHz, DMSO‐*d*_*6*_) *δ* 169.92, 169.47, 163.60, 162.81, 162.51, 159.84 (d, *J* = 4.4 Hz), 157.85, 150.29, 145.38, 142.90, 137.71, 134.93 (d, *J* = 3.5 Hz), 133.99, 133.52 (d, *J* = 8.2 Hz), 132.02, 131.03, 129.54, 128.37, 127.52, 126.55, 125.97, 123.12, 123.01, 117.75, 116.88, 116.70, 114.28, 101.45, 98.28, 70.80, 66.60, 46.68, 43.05, 39.12, 36.99, 36.70, 18.50, 13.70. HR‐MS (ESI) Calcd. for C_37_H_34_FN_9_O_4_ [M + Na] ^+^: *m/z* 710.2610, Found 710.2618.

*(R)*‐*2*‐*fluoro*‐*N*‐*(3*‐*((1*‐*(6*‐*(3*‐*methylmorpholino)*‐*2*‐*(1H*‐*pyrrolo* *[2,3*‐*b]pyridin*‐*4*‐*yl)pyrimi* *din*‐*4*‐*yl)cyclopropyl)amino)*‐*3*‐*oxopropyl)*‐*5*‐*((4*‐*oxo*‐*3,4*‐*dihydrophthalazin*‐*1*‐*yl)methyl)benza* *mide* *(* ***A3*** *)*. White solid, yield 61%. m.p. 229.7–233.4 °C. Purity: 96.18% by analytical HPLC. ^1^H NMR (500 MHz, DMSO‐*d*_*6*_) δ 12.59 (s, 1H), 11.76 (s, 1H), 8.60 (s, 1H), 8.34–8.23 (m, 3H), 7.99–7.79 (m, 3H), 7.65–7.53 (m, 3H), 7.45 (ddd, *J* = 8.5, 4.8, 2.4 Hz, 1H), 7.19 (dd, *J* = 10.4, 8.5 Hz, 1H), 7.08 (s, 1H), 7.04 (dd, *J* = 3.5, 2.0 Hz, 1H), 4.57–4.46 (m, 1H), 4.33 (s, 2H), 4.14 (d, *J* = 13.4 Hz, 1H), 3.95 (dd, *J* = 11.3, 3.7 Hz, 1H), 3.74 (d, *J* = 11.3 Hz, 1H), 3.60 (dd, *J* = 11.7, 3.1 Hz, 1H), 3.55–3.41 (m, 3H), 3.15 (td, *J* = 13.0, 4.0 Hz, 1H), 2.47 (t, *J* = 7.4 Hz, 2H), 1.62–1.54 (m, 1H), 1.49 (ddd, *J* = 15.1, 7.6, 5.1 Hz, 1H), 1.20 (d, *J* = 6.7 Hz, 3H), 1.14 (t, *J* = 4.3 Hz, 2H). ^13^C NMR (126 MHz, DMSO‐d_6_) *δ* 171.93, 169.69, 163.71, 162.79, 162.55, 159.88, 159.45, 157.48, 150.29, 145.39, 142.86, 137.74, 134.85 (d, *J* = 3.2 Hz), 134.00, 133.08 (d, *J* = 7.4 Hz), 132.03, 130.68 (d, *J* = 2.4 Hz), 129.55, 128.38, 127.46, 126.55, 125.98, 124.04, 123.92, 117.79, 116.75, 116.57, 114.18, 101.61, 98.25, 70.78, 66.56, 46.66 (d, *J* = 3.4 Hz), 39.11, 36.96, 36.57, 36.47, 35.58, 18.72, 13.70. HR‐MS (ESI) Calcd. for C_38_H_36_FN_9_O_4_ [M + Na]^+^: *m/z* 724.2766, Found 724.2746.

*(R)*‐*2*‐*fluoro*‐*N*‐*(4‐((1*‐*(6‐(3‐methylmorpholino)‐2‐(1H‐pyrrolo[2,3‐b]pyridin‐4‐yl)pyrimidin‐4‐yl)cyclopropyl)amino)*‐*4‐oxobutyl)‐5‐((4‐oxo‐3,4‐dihydrophthalazin‐1‐yl)methyl)benzamide* *(* ***A4*** *)*. White solid, yield 73%. m.p. 241.6‐242.7 °C. Purity: 97.41% by analytical HPLC. ^1^H NMR (500 MHz, DMSO‐*d*_*6*_) *δ* 12.59 (s, 1H), 11.77 (s, 1H), 8.52 (s, 1H), 8.39 – 8.20 (m, 3H), 7.97 (d, *J* = 8.0 Hz, 1H), 7.92 – 7.79 (m, 2H), 7.64–7.53 (m, 3H), 7.47 – 7.41 (m, 1H), 7.20 (dd, *J* = 10.2, 8.5 Hz, 1H), 7.07 (s, 1H), 7.01 (dd, *J* = 3.4, 2.0 Hz, 1H), 4.56 – 4.46 (m, 1H), 4.32 (s, 2H), 4.14 (d, *J* = 13.4 Hz, 1H), 3.94 (dd, *J* = 11.4, 3.8 Hz, 1H), 3.72 (d, *J* = 11.4 Hz, 1H), 3.59 (dd, *J* = 11.5, 3.2 Hz, 1H), 3.45 (td, *J* = 11.7, 3.0 Hz, 1H), 3.25 (q, *J* = 6.7 Hz, 2H), 3.20 – 3.11 (m, 1H), 2.23 (t, *J* = 7.6 Hz, 2H), 1.78 (p, *J* = 7.4 Hz, 2H), 1.54 (ddt, *J* = 37.1, 9.8, 2.9 Hz, 2H), 1.19 (d, *J* = 6.7 Hz, 3H). ^13^C NMR (126 MHz, DMSO‐*d*_*6*_) *δ* 173.24, 169.86, 163.96, 162.76, 162.52, 159.88, 159.31, 157.34, 150.29, 145.41, 142.86, 137.77, 134.78 (d, *J* = 3.3 Hz), 133.99, 132.78 (d, *J* = 7.9 Hz), 132.02, 130.53 (d, *J* = 2.7 Hz), 129.54, 128.38, 127.44, 126.54, 125.99, 124.67, 124.55, 117.76, 116.72, 116.54, 114.22, 101.54, 98.25, 70.75, 66.55, 46.65, 39.09, 36.96, 36.60, 33.57, 25.67, 18.79 (d, *J* = 3.8 Hz), 13.71. HR‐MS (ESI) Calcd. for C_39_H_38_FN_9_O_4_ [M + H]^+^: *m/z* 716.3104, Found 716.3095.

*(R)‐1‐(2‐fluoro‐5‐((4‐oxo‐3,4‐dihydrophthalazin‐1‐yl)methyl)benzoyl)‐N‐(1‐(6‐(3‐methylmorpholino)‐2‐(1H‐pyrrolo[2,3‐b]pyridin‐4‐yl)pyrimidin‐4‐yl)cyclopropyll)piperidine‐4‐carboxamid* *e* *(* ***A5*** *)*. White solid, yield 65%. m.p. 238.7–239.6 °C. Purity: 99.05% by analytical HPLC. ^1^H NMR (500 MHz, DMSO‐*d*_*6*_) *δ* 12.59 (s, 1H), 11.78 (s, 1H), 8.53 (s, 1H), 8.27 (dd, *J* = 18.2, 6.4 Hz, 2H), 7.97 (d, *J* = 8.0 Hz, 1H), 7.83 (dd, *J* = 29.6, 5.9 Hz, 2H), 7.60 (d, *J* = 5.0 Hz, 1H), 7.54 (t, *J* = 3.0 Hz, 1H), 7.37 (d, *J* = 29.9 Hz, 2H), 7.22 (t, *J* = 9.0 Hz, 1H), 7.07 (s, 1H), 7.00 (s, 1H), 4.60 – 4.38 (m, 2H), 4.33 (s, 2H), 4.21 – 4.08 (m, 1H), 3.94 (dt, *J* = 10.7, 2.8 Hz, 1H), 3.73 (d, *J* = 11.4 Hz, 1H), 3.60 (dd, *J* = 11.4, 3.2 Hz, 1H), 3.46 (tt, *J* = 11.9, 2.7 Hz, 1H), 3.37 (d, *J* = 14.8 Hz, 1H), 3.22 – 3.11 (m, 1H), 3.03 (s, 1H), 2.85 (t, *J* = 12.2 Hz, 1H), 1.87 (d, *J* = 12.9 Hz, 1H), 1.74 – 1.42 (m, 5H), 1.23 – 1.18 (m, 3H), 1.16 – 1.09 (m, 2H). ^13^C NMR (126 MHz, DMSO‐*d*_*6*_) *δ* 174.86, 169.82, 164.18, 162.74, 162.52, 159.85, 150.28, 145.41, 142.85, 137.84, 135.31 (d, *J* = 2.9 Hz), 133.93, 132.01, 131.83 (d, *J* = 7.6 Hz), 129.54, 129.08 (d, *J* = 3.4 Hz), 128.38, 127.42, 126.55, 125.95, 124.79, 124.64, 117.72, 116.39, 116.23, 114.24, 101.55, 98.33, 70.75, 66.54, 60.23, 46.64, 41.95, 41.20, 39.09, 36.91, 36.49, 28.60 (d, *J* = 6.9 Hz), 18.96, 13.77. HR‐MS (ESI) Calcd. for C_41_H_40_FN_9_O_4_ [M + H]^+^: *m/z* 742.3260, Found 742.3254.

### Synthesis of Compound **A6**

*(R)‐4‐(2‐fluoro‐5‐((4‐oxo‐3,4‐dihydrophthalazin‐1‐yl)methyl)benzoyl)‐N‐(1‐(6‐(3‐methylmorpholino)‐2‐(1H‐pyrrolo[2,3‐b]pyridin‐4‐yl)pyrimidin‐4‐yl)cyclopropyll)piperazine‐1‐carboxamide* *(* ***A6*** *)*. To a solution of intermediate **23** (100 mg, 0.29 mmol) in DMF (5 mL) were added CDI (51.9 mg, 0.32 mmol) and DIPEA (112.2 mg, 0.87 mmol). The resulting solution was stirred at room temperature for 1 h. Then, commercially available starting material **34** (128.2 mg, 0.35 mmol) was added to the above reaction system. The resulting solution was stirred at room temperature for 4 h. The reaction was monitored by TLC. Upon completion, the mixture was quenched with cool water (50 mL) at room temperature and extracted with EtOAc (50 mL × 3). The combined organic layers were washed with water, brine, and dried over Na_2_SO_4_. The drying agent was filtered off. The filtrate was concentrated under reduced pressure, and the residue was purified via flash column chromatography (CH_2_Cl_2_/MeOH 47:3, *v/v*) to give compound **A6** as a white solid, yield of 56%. m.p. 250.6–254.7 °C. Purity: 97.07% by analytical HPLC. ^1^H NMR (500 MHz, DMSO‐*d*_*6*_) *δ* 12.60 (s, 1H), 11.77 (s, 1H), 8.32–8.25 (m, 2H), 7.97 (d, *J* = 7.7 Hz, 1H), 7.89 (td, *J* = 8.2, 7.8, 1.5 Hz, 1H), 7.85 – 7.80 (m, 1H), 7.62 (d, *J* = 5.0 Hz, 1H), 7.54 – 7.49 (m, 1H), 7.46 – 7.37 (m, 3H), 7.25 (t, *J* = 9.0 Hz, 1H), 7.08 (s, 1H), 7.04 (dd, *J* = 3.4, 2.0 Hz, 1H), 4.53 (d, *J* = 4.5 Hz, 1H), 4.34 (s, 2H), 4.13 (d, *J* = 13.4 Hz, 1H), 3.95 (dd, *J* = 11.3, 3.7 Hz, 1H), 3.75 (d, *J* = 11.3 Hz, 1H), 3.69 – 3.40 (m, 7H), 3.24 – 3.12 (m, 3H), 1.62 – 1.55 (m, 1H), 1.52 – 1.46 (m, 1H), 1.20 (d, *J* = 6.7 Hz, 3H), 1.18 – 1.13 (m, 2H). ^13^C NMR (126 MHz, DMSO‐*d*_*6*_) *δ* 170.85, 164.54, 162.75, 162.49, 159.87, 158.40, 150.33, 145.36, 142.86, 137.88, 135.34 (d, *J* = 2.6 Hz), 133.96, 132.05, 129.50 (d, *J* = 16.4 Hz), 128.38, 127.26, 126.56, 125.93, 124.28, 124.14, 117.79, 116.50, 116.33, 114.21, 101.74, 98.07, 70.80, 66.55, 46.94, 46.63, 44.10, 43.62, 41.98, 37.82, 36.92, 19.04 (d, *J* = 11.8 Hz), 13.74. HR‐MS (ESI) Calcd. for C_40_H_39_FN_10_O_4_ [M + H]^+^: m/z 743.3213, Found 743.3205.

### Synthesis of Compound **A7**

Compound **A7** was synthesized according to the procedure described for compounds **A2‐A5**. *(R)* *‐4‐(4‐(2‐fluoro‐5‐((4‐oxo‐3,4‐dihydrophthalazin‐1‐yl)methyl)benzoyl)piperazine‐1‐carbonyl)‐N‐(1‐(6‐(3‐methylmorpholino)‐2‐(1H‐pyrrolo[2,3‐b]pyridin‐4‐yl)pyrimidin‐4‐yl)cyclopropyl)benzamide* *(* ***A7*** *)*. White solid, yield 72%. m.p. 265.8–266.2 °C. Purity: 97.76% by analytical HPLC. ^1^H NMR (500 MHz, DMSO‐*d*_*6*_) *δ* 12.60 (s, 1H), 11.71 (s, 1H), 9.23 (s, 1H), 8.27 (d, *J* = 5.0 Hz, 2H), 8.11 – 7.68 (m, 5H), 7.68 – 7.18 (m, 7H), 7.11 (s, 1H), 6.99 (dd, *J* = 3.4, 2.0 Hz, 1H), 4.56 – 4.43 (m, 1H), 4.34 (s, 2H), 4.10 (d, *J* = 13.0 Hz, 1H), 3.91 (d, *J* = 10.9 Hz, 1H), 3.63 (dd, *J* = 52.0, 11.2 Hz, 6H), 3.43 (dd, *J* = 24.8, 12.9 Hz, 2H), 3.27 – 3.05 (m, 3H), 1.76 – 1.67 (m, 1H), 1.66 – 1.56 (m, 1H), 1.38 – 1.26 (m, 2H), 1.16 (t, *J* = 6.3 Hz, 3H). ^13^C NMR (126 MHz, DMSO‐*d*_*6*_) *δ* 172.97, 169.65, 167.42, 164.59, 162.86, 162.56 (d, *J* = 8.6 Hz), 162.42, 159.89, 150.27 (d, *J* = 2.3 Hz), 142.93, 142.84, 137.81, 137.72, 135.32 (d, *J* = 2.3 Hz), 133.93, 133.02, 129.56, 128.38, 128.08, 127.67, 127.32, 126.56, 125.93, 117.76, 117.69, 114.36, 114.16, 101.24, 98.04, 70.76 (d, *J* = 4.0 Hz), 66.59, 66.52, 46.70, 39.33, 39.12, 36.92, 19.50, 19.39, 13.71. HR‐MS (ESI) Calcd. for C_47_H_43_FN_10_O_5_ [M + H]^+^: m/z 847.3475, Found 847.3471.

### Synthesis of Compound **A8**

To a solution of intermediates **38a** (0.5 mmol) in MeOH (8 mL) was added a solution of NaOH (1.5 mmol) in H_2_O (2 mL). The mixture was stirred at room temperature for 2 h. The reaction was monitored by TLC. Upon completion, the mixture was diluted with cool water (50 mL), and the pH was adjusted to 2 ∼ 3 using aqueous HCl solution (1 N). The mixture was extracted with EtOAc (50 mL × 2). The combined organic layers were washed with water and brine and dried over Na_2_SO_4_. The drying agent was filtered off. The filtrate was concentrated under reduced pressure to afford the corresponding acids as a white solid, which was used directly in the next stage without further purification.

To a solution of corresponding acids (0.28 mmol, crude material from the previous step) and DIPEA (1.40 mmol) in DMF (7 mL) were added **34** (0.31 mmol), HOBT (0.36 mmol), and EDCI (0.73 mmol). The resulting solution was stirred at room temperature for 3 h. The reaction was monitored by TLC. Upon completion, the mixture was quenched with cool water (70 mL) at room temperature and extracted with EtOAc (70 mL × 3). The combined organic layers were washed with water and brine and dried over Na_2_SO_4_. The drying agent was filtered off. The filtrate was concentrated under reduced pressure, and the residue was purified via flash column chromatography (CH_2_Cl_2_/MeOH 93:7, v/v) to give compound **A8** as a white solid, yield 63%. *(R)‐4‐(4‐(2‐fluoro‐5‐((4‐oxo‐3,4‐dihydrophthalazin‐1‐yl)methyl)benzoyl)piperazine‐1‐carbony l)‐N‐(1‐(6‐(3‐methylmorpholino)‐2‐(1H‐pyrrolo[2,3‐b]pyridin‐4‐yl)pyrimidin‐4‐yl)cyclopropyl)benzenesulfonamide (* ***A8***). m.p. 245.7‐246.3 °C. Purity: 98.92% by analytical HPLC. ^1^H NMR (500 MHz, DMSO‐*d*_6_) *δ* 12.60 (s, 1H), 11.80 (s, 1H), 8.71 (s, 1H), 8.41 – 8.12 (m, 2H), 8.06 – 7.68 (m, 5H), 7.66 – 7.14 (m, 7H), 7.07 – 6.72 (m, 2H), 4.33 (s, 4H), 3.94 (d, *J* = 11.0 Hz, 1H), 3.82 – 3.39 (m, 7H), 3.29 – 2.87 (m, 5H), 1.61 – 1.41 (m, 3H), 1.37 – 1.28 (m, 1H), 1.17 (d, *J* = 6.8 Hz, 3H). ^13^C NMR (126 MHz, DMSO‐*d*_*6*_) *δ* 168.34, 164.51, 162.35, 161.81, 159.88, 150.18, 145.30, 142.86, 138.95, 137.50, 135.32 (d, *J* = 3.6 Hz), 133.96, 132.02, 129.56, 129.40 (d, *J* = 4.8 Hz), 128.38, 127.70, 127.18, 126.56, 125.93, 117.53, 116.51, 116.34, 114.33, 101.06, 98.15, 70.69, 66.63, 46.66, 39.20, 36.91, 17.49, 17.11, 13.86. HR‐MS (ESI) Calcd. for C_46_H_43_FN_10_O_6_S [M + H] ^+^: *m/z* 883.3145, Found 883.3133.

### General Synthetic Procedure for Compounds **A9**–**A12**

Compounds **A9–A12** were synthesized according to the procedure described for compounds **A2–A5**.

*(R)‐6‐(4‐(2‐fluoro‐5‐((4‐oxo‐3,4‐dihydrophthalazin‐1‐yl)methyl)benzoyl)piperazine‐1‐carbonyl)‐N‐(1‐(6‐(3‐methylmorpholino)‐2‐(1H‐pyrrolo[2,3‐b]pyridin‐4‐yl)pyrimidin‐4‐yl)cyclo propyl)nicotinamide* *(* ***A9*** *)*. White solid, yield 70%. m.p. 257.2–259.1 °C. Purity: 98.50% by analytical HPLC. ^1^H NMR (500 MHz, DMSO‐*d*_*6*_) *δ* 12.60 (d, *J* = 17.2 Hz, 1H), 11.72 (d, *J* = 13.2 Hz, 1H), 9.45 (s, 1H), 9.08 (dd, *J* = 23.5, 2.2 Hz, 1H), 8.40 (dt, *J* = 8.2, 2.6 Hz, 1H), 8.32 – 8.19 (m, 2H), 8.05 – 7.70 (m, 4H), 7.60 (dd, *J* = 5.1, 2.0 Hz, 1H), 7.53 – 7.18 (m, 4H), 7.12 (d, *J* = 2.9 Hz, 1H), 6.98 (dt, *J* = 5.7, 2.7 Hz, 1H), 4.48 (s, 1H), 4.34 (d, *J* = 21.2 Hz, 2H), 4.18 – 4.05 (m, 1H), 3.99 – 3.86 (m, 1H), 3.82 – 3.40 (m, 8H), 3.25 –3.09 (m, 2H), 1.73 (dt, *J* = 6.8, 4.8 Hz, 1H), 1.64 (dt, *J* = 9.9, 4.7 Hz, 1H), 1.39 – 1.28 (m, 2H), 1.16 (t, *J* = 7.1 Hz, 3H). HR‐MS (ESI) Calcd. for C_46_H_42_FN_11_O_5_ [M + H] ^+^: *m/z* 848.3427, Found 848.3408.

*(R)‐4‐((4‐(2‐fluoro‐5‐((4‐oxo‐3,4‐dihydrophthalazin‐1‐yl)methyl)benzoyl)piperazin‐1‐yl) methyl)‐N‐(1‐(6‐(3‐methylmorpholino)‐2‐(1H‐pyrrolo[2,3‐b]pyridin‐4‐yl)pyrimidin‐4‐yl)cyclopropyl)benzamide* *(* ***A10*** *)*. White solid, yield 69%. m.p. 247.2 – 248.1 °C. Purity: 97.75% by analytical HPLC. ^1^H NMR (500 MHz, DMSO‐*d*_*6*_) *δ* 12.60 (s, 1H), 11.70 (s, 1H), 9.08 (s, 1H), 8.33 – 8.21 (m, 2H), 8.01 – 7.75 (m, 5H), 7.60 (d, *J* = 5.1 Hz, 1H), 7.47 – 7.37 (m, 3H), 7.32 (t, *J* = 3.1 Hz, 2H), 7.22 (t, *J* = 9.0 Hz, 1H), 7.10 (s, 1H), 7.00 (dd, *J* = 3.4, 2.0 Hz, 1H), 4.47 (d, *J* = 8.1 Hz, 1H), 4.33 (s, 2H), 4.09 (d, *J* = 13.2 Hz, 1H), 3.90 (dd, *J* = 11.5, 3.7 Hz, 1H), 3.75 – 3.50 (m, 6H), 3.44 (td, *J* = 11.6, 3.0 Hz, 1H), 3.25 – 3.07 (m, 3H), 2.41 (s, 2H), 2.28 (s, 2H), 1.70 (q, *J* = 5.5, 3.9 Hz, 1H), 1.65 – 1.57 (m, 1H), 1.31 (tdd, *J* = 9.6, 8.4, 6.9, 3.1 Hz, 2H), 1.14 (d, *J* = 6.7 Hz, 3H). ^13^C NMR (126 MHz, DMSO‐*d*_*6*_) *δ* 169.80, 167.92, 164.24, 162.84, 162.58, 159.88, 157.80, 155.85, 150.26, 145.37, 142.82, 137.73, 135.30 (d, *J* = 3.2 Hz), 133.95, 132.03, 129.55, 129.24, 129.04, 128.38, 127.93, 127.14, 126.56, 125.95, 117.77, 116.49, 116.32, 114.15, 101.89, 98.29, 70.75, 66.51, 61.81, 53.15, 52.59, 46.62, 39.10 (d, *J* = 2.1 Hz), 37.25, 36.88, 18.87 (d, *J* = 7.2 Hz), 13.70. HR‐MS (ESI) Calcd. for C_47_H_45_FN_10_O_4_ [M + H] ^+^: *m/z* 833.3682, Found 833.3690.

*(R)‐4‐(4‐(2‐fluoro‐5‐((4‐oxo‐3,4‐dihydrophthalazin‐1‐yl)methyl)benzoyl)piperazin‐1‐yl)‐N‐(1‐(6‐(3‐methylmorpholino)‐2‐(1H‐pyrrolo[2,3‐b]pyridin‐4‐yl)pyrimidin‐4‐yl)cyclopropyl)benzamide* *(* ***A11*** *)*. White solid, yield 79%. m.p. 243.3–244.6 °C. Purity: 97.49% by analytical HPLC. ^1^H NMR (500 MHz, DMSO‐*d*_*6*_) *δ* 12.60 (s, 1H), 11.69 (s, 1H), 8.87 (s, 1H), 8.37 – 8.18 (m, 2H), 7.98 (d, *J* = 8.0 Hz, 1H), 7.95 – 7.80 (m, 4H), 7.60 (d, *J* = 5.0 Hz, 1H), 7.50 – 7.37 (m, 2H), 7.34 (t, *J* = 3.0 Hz, 1H), 7.26 (t, *J* = 9.0 Hz, 1H), 7.09 (s, 1H), 7.05 – 6.94 (m, 3H), 4.47 (dd, *J* = 12.9, 5.1 Hz, 1H), 4.35 (s, 2H), 4.09 (d, *J* = 13.2 Hz, 1H), 3.90 (dd, *J* = 11.7, 3.6 Hz, 1H), 3.78 (s, 2H), 3.68 (d, *J* = 11.4 Hz, 1H), 3.57 (dd, *J* = 11.6, 3.2 Hz, 1H), 3.48 – 3.34 (m, 4H), 3.26 – 3.07 (m, 3H), 1.74 – 1.64 (m, 1H), 1.59 (td, *J* = 6.7, 6.3, 3.8 Hz, 1H), 1.28 (pd, *J* = 9.4, 2.8 Hz, 2H), 1.14 (d, *J* = 6.7 Hz, 3H). ^13^C NMR (126 MHz, DMSO‐*d*_*6*_) *δ* 170.09, 167.69, 164.37, 162.81, 162.56, 159.89, 157.86, 155.93, 152.74, 150.26, 145.37, 142.81, 137.75, 135.33 (d, *J* = 2.6 Hz), 134.00, 132.22 (d, *J* = 7.4 Hz), 132.06, 129.58, 129.45 (d, *J* = 3.2 Hz), 129.23, 128.39, 127.16, 126.56, 125.95, 125.59, 124.21, 124.06, 117.77, 116.51, 116.34, 114.47, 114.13, 101.94, 98.20, 70.76, 66.52, 48.07, 47.75, 46.61, 41.56, 39.09, 37.21, 36.91, 18.93, 13.68. HR‐MS (ESI) Calcd. for C_46_H_43_FN_10_O_4_ [M + H]^+^: *m/z* 843.3138, Found 843.3123.

*(R)‐2‐(4‐(2‐fluoro‐5‐((4‐oxo‐3,4‐dihydrophthalazin‐1‐yl)methyl)benzoyl)piperazin‐1‐yl)‐N‐(1‐(6‐(3‐methylmorpholino)‐2‐(1H‐pyrrolo[2,3‐b]pyridin‐4‐yl)pyrimidin‐4‐yl)cyclopropyl)pyrimidine‐5‐carboxamide* *(* ***A12*** *)*. White solid, yield 81%. m.p. 261.2–261.9 °C. Purity: 99.15% by analytical HPLC. ^1^H NMR (500 MHz, DMSO‐*d*_*6*_) *δ* 12.61 (s, 1H), 11.71 (s, 1H), 9.07 (s, 1H), 8.88 (s, 2H), 8.36 – 8.21 (m, 2H), 7.99 (d, *J* = 8.0 Hz, 1H), 7.91 (td, *J* = 8.2, 7.8, 1.5 Hz, 1H), 7.84 (td, *J* = 7.5, 1.2 Hz, 1H), 7.60 (d, *J* = 5.1 Hz, 1H), 7.51 – 7.39 (m, 2H), 7.38 – 7.33 (m, 1H), 7.26 (t, *J* = 9.0 Hz, 1H), 7.11 (s, 1H), 6.99 (dd, *J* = 3.5, 2.0 Hz, 1H), 4.47 (d, *J* = 8.0 Hz, 1H), 4.35 (s, 2H), 4.17 – 4.05 (m, 1H), 3.92 (d, *J* = 13.5 Hz, 3H), 3.86 – 3.66 (m, 5H), 3.59 (dd, *J* = 11.5, 3.1 Hz, 1H), 3.45 (td, *J* = 11.7, 3.0 Hz, 1H), 3.31 (t, *J* = 5.3 Hz, 2H), 3.19 – 3.10 (m, 1H), 1.75 – 1.65 (m, 1H), 1.65 – 1.57 (m, 1H), 1.34 – 1.25 (m, 2H), 1.15 (d, *J* = 6.7 Hz, 3H). ^13^C NMR (126 MHz, DMSO‐*d*_*6*_) *δ* 169.67, 165.28, 164.61, 162.82, 162.59, 161.90, 159.89, 158.22, 157.89, 155.94, 150.26, 145.35, 142.88, 137.74, 135.31 (d, *J* = 3.4 Hz), 133.99, 132.26 (d, *J* = 8.3 Hz), 132.06, 129.59, 129.52 (d, *J* = 3.0 Hz), 128.38, 127.13, 126.57, 125.94, 124.21, 124.07, 117.75, 117.58, 116.53, 116.37, 114.16, 101.74, 98.35, 70.75, 66.51, 46.65, 44.13, 43.63, 41.70, 39.12 (d, *J* = 3.2 Hz), 37.04, 36.92, 18.74, 13.69. HR‐MS (ESI) Calcd. for C_44_H_41_FN_12_O_4_ [M + H] ^+^: *m/z* 843.3250, Found 843.3255.

### Synthesis of Compound **B1**

To a solution of intermediate **28** (50 mg, 0.15 mmol) in DMF (3 mL) were added **29** (59.65 mg, 0.20 mmol), DIPEA (59.60 mg, 0.46 mmol), HOBT (27.02 mg, 0.20 mmol), and EDCI (76.68 mg, 0.40 mmol). The resulting solution was stirred at room temperature for 3 h. The reaction was monitored by TLC. Upon completion, the mixture was quenched with cool water (30 mL) at room temperature and extracted with EtOAc (30 mL × 3). The combined organic layers were washed with water and brine and dried over Na_2_SO_4_. The drying agent was filtered off. The filtrate was concentrated under reduced pressure, and the residue was purified via flash column chromatography (CH_2_Cl_2_/MeOH 19:1, *v/v*) to give compound **B1** as a white solid, yield 74%. *(R)‐2‐fluoro‐N‐((6‐(3‐methylmorpholino)‐2‐(1H‐pyrrolo[2,3‐b]pyridin‐4‐yl)pyrimidin‐4‐yl)methyl)‐5‐((4‐oxo‐3,4‐dihydrophthalazin‐1‐yl)methyl)benzamide (* ***B1***). m.p. 223.5–225.5 °C. Purity: 95.51% by analytical HPLC. ^1^H NMR (500 MHz, DMSO‐*d*_*6*_) *δ* 12.60 (s, 1H), 11.75 (s, 1H), 8.89 (td, *J* = 5.9, 2.8 Hz, 1H), 8.38 – 8.22 (m, 2H), 8.04 – 7.94 (m, 2H), 7.84 (dtd, *J* = 20.7, 7.3, 1.4 Hz, 2H), 7.69 (dd, *J* = 6.9, 2.4 Hz, 1H), 7.58 – 7.48 (m, 2H), 7.37 – 7.23 (m, 2H), 6.70 (s, 1H), 4.53 (dd, *J* = 5.9, 2.5 Hz, 3H), 4.35 (s, 2H), 4.11 (d, *J* = 13.3 Hz, 1H), 4.00 (dd, *J* = 11.4, 3.8 Hz, 1H), 3.79 (d, *J* = 11.4 Hz, 1H), 3.66 (dd, *J* = 11.5, 3.2 Hz, 1H), 3.51 (td, *J* = 11.8, 3.1 Hz, 1H), 3.25 (td, *J* = 12.8, 3.9 Hz, 1H), 1.24 (d, *J* = 6.8 Hz, 3H). ^13^C NMR (126 MHz, DMSO‐*d*_*6*_) *δ* 166.36, 164.31, 163.30, 162.52, 159.88, 159.62, 157.64, 150.67, 145.39, 142.66, 137.21, 135.00 (d, *J* = 3.0 Hz), 133.98, 133.27 (d, *J* = 8.6 Hz), 132.03, 130.71 (d, *J* = 2.9 Hz), 129.55, 128.39, 127.54, 126.56, 125.97, 124.02, 123.90, 118.30, 116.89, 116.70, 115.14, 102.47, 98.43, 70.68, 66.49, 60.23, 47.03, 44.89, 36.97, 13.66. HR‐MS (ESI) Calcd. for C_33_H_29_FN_8_O_3_ [M + H] ^+^: *m/z* 605.2419, Found 605.2418.

### General Synthetic Procedure for Compounds **B2**–**B5**

Compounds **B2–B5** were synthesized according to the procedure described for compounds **A2–A5**.

*(R)‐2‐fluoro‐N‐(2‐(((6‐(3‐methylmorpholino)‐2‐(1H‐pyrrolo[2,3‐b]pyridin‐4‐yl)pyrimidin‐4‐yl)methyl)amino)‐2‐oxoethyl)‐5‐((4‐oxo‐3,4‐dihydrophthalazin‐1‐yl)methyl)benzamide* *(* ***B2*** *)*. White solid, yield 74%. m.p. 252.4‐253.2 °C. Purity: 98.0% by analytical HPLC. ^1^H NMR (500 MHz, DMSO‐*d*_*6*_) *δ* 12.59 (s, 1H), 11.75 (s, 1H), 8.60 (p, *J* = 6.0, 5.6 Hz, 2H), 8.37 – 8.21 (m, 2H), 8.03 – 7.94 (m, 2H), 7.84 (dtd, *J* = 28.0, 7.3, 1.3 Hz, 2H), 7.73 (dd, *J* = 7.1, 2.4 Hz, 1H), 7.62 – 7.48 (m, 2H), 7.33 – 7.22 (m, 2H), 6.70 (s, 1H), 4.60 (s, 1H), 4.42 – 4.31 (m, 4H), 4.22 (d, *J* = 12.8 Hz, 1H), 3.96 (dd, *J* = 13.9, 4.6 Hz, 3H), 3.73 (d, *J* = 11.3 Hz, 1H), 3.61 (dd, *J* = 11.5, 3.1 Hz, 1H), 3.46 (td, *J* = 11.8, 3.0 Hz, 1H), 3.23 (td, *J* = 12.9, 3.9 Hz, 1H), 1.22 (d, *J* = 6.7 Hz, 3H). ^13^C NMR (126 MHz, DMSO‐*d*_*6*_) *δ* 169.61, 166.82, 164.30, 163.15, 162.63, 159.97, 159.87, 157.99, 150.64, 145.33, 142.66, 137.26, 134.85 (d, *J* = 2.9 Hz), 133.96, 133.72 (d, *J* = 8.9 Hz), 132.02, 131.04 (d, *J* = 2.0 Hz), 129.55, 128.37, 127.56, 126.56, 125.93, 122.97, 122.86, 118.28, 116.94, 116.76, 115.17, 102.42, 98.19, 70.69, 66.54, 60.24, 46.89, 44.29, 43.83, 37.04, 21.23, 14.55, 13.78. HR‐MS (ESI) Calcd. for C_35_H_32_FN_9_O_4_ [M + H] ^+^: *m/z* 662.2634, Found 662.2637.

*(R)‐2‐fluoro‐N‐(3‐(((6‐(3‐methylmorpholino)‐2‐(1H‐pyrrolo[2,3‐b]pyridin‐4‐yl)pyrimidin‐4‐yl)methyl)amino)‐3‐oxopropyl)‐5‐((4‐oxo‐3,4‐dihydrophthalazin‐1‐yl)methyl)benzamide* *(* ***B3*** *)*. White solid, yield 51%. m.p. 258.7–260.1 °C. Purity: 96.97% by analytical HPLC. ^1^H NMR (500 MHz, DMSO‐*d*_*6*_) *δ* 12.60 (s, 1H), 11.74 (s, 1H), 8.52 (t, *J* = 6.0 Hz, 1H), 8.32 (dd, *J* = 11.4, 4.2 Hz, 2H), 8.26 (dd, *J* = 7.9, 1.4 Hz, 1H), 8.03 – 7.92 (m, 2H), 7.91 – 7.78 (m, 2H), 7.63 – 7.53 (m, 2H), 7.46 (ddd, *J* = 8.5, 4.8, 2.4 Hz, 1H), 7.27 (dd, *J* = 3.4, 2.0 Hz, 1H), 7.20 (dd, *J* = 10.5, 8.5 Hz, 1H), 6.63 (s, 1H), 4.53 (s, 1H), 4.41 – 4.28 (m, 4H), 4.17 – 4.06 (m, 1H), 3.96 (dd, *J* = 11.5, 3.7 Hz, 1H), 3.76 (d, *J* = 11.4 Hz, 1H), 3.63 (dd, *J* = 11.5, 3.2 Hz, 1H), 3.58 – 3.43 (m, 3H), 3.27 – 3.16 (m, 1H), 2.53 (t, *J* = 7.3 Hz, 2H), 1.22 (d, *J* = 6.8 Hz, 3H). ^13^C NMR (126 MHz, DMSO‐*d*_*6*_) *δ* 171.29, 166.77, 163.83, 163.23, 162.53, 159.87, 159.45, 157.48, 150.65, 145.38, 142.65, 137.24, 134.85 (d, *J* = 3.2 Hz), 133.99, 133.08 (d, *J* = 8.3 Hz), 132.02, 130.65 (d, *J* = 2.5 Hz), 129.53, 128.37, 127.55, 126.54, 125.96, 124.07, 123.96, 118.30, 116.76, 116.57, 115.15, 102.48, 98.52, 70.69, 66.50, 60.23, 46.91, 44.34, 39.36, 36.96, 36.50, 35.54, 21.23, 14.55, 13.67. HR‐MS (ESI) Calcd. for C_36_H_34_FN_9_O_4_ [M + H] ^+^: *m/z* 676.2791, Found 676.2794.

*(R)‐2‐fluoro‐N‐(4‐(((6‐(3‐methylmorpholino)‐2‐(1H‐pyrrolo[2,3‐b]pyridin‐4‐yl)pyrimidin‐4‐yl)methyl)amino)‐4‐oxobutyl)‐5‐((4‐oxo‐3,4‐dihydrophthalazin‐1‐yl)methyl)benzamide* *(* ***B4*** *)*. White solid, yield 67%. m.p. 221.7–222.4 °C. Purity: 95.23% by analytical HPLC. ^1^H NMR (500 MHz, DMSO‐*d*_*6*_) *δ* 12.59 (s, 1H), 11.75 (s, 1H), 8.44 (t, *J* = 6.0 Hz, 1H), 8.38 – 8.24 (m, 3H), 8.03 – 7.93 (m, 2H), 7.85 (dtd, *J* = 29.8, 7.3, 1.4 Hz, 2H), 7.62 – 7.54 (m, 2H), 7.44 (ddd, *J* = 8.4, 4.9, 2.4 Hz, 1H), 7.32 – 7.17 (m, 2H), 6.63 (s, 1H), 4.52 (s, 1H), 4.40 – 4.27 (m, 4H), 4.12 (dd, *J* = 17.5, 9.1 Hz, 1H), 3.97 (dd, *J* = 11.4, 3.8 Hz, 1H), 3.76 (d, *J* = 11.4 Hz, 1H), 3.64 (dd, *J* = 11.5, 3.1 Hz, 1H), 3.49 (td, *J* = 11.8, 3.1 Hz, 1H), 3.32 – 3.19 (m, 3H), 2.30 (t, *J* = 7.4 Hz, 2H), 1.81 (p, *J* = 7.3 Hz, 2H), 1.23 (d, *J* = 6.8 Hz, 3H). ^13^C NMR (126 MHz, DMSO‐*d*_*6*_) *δ* 172.67, 167.03, 164.05, 163.23, 162.53, 159.88, 159.30, 157.33, 150.65, 145.40, 142.66, 137.25, 134.80 (d, *J* = 3.4 Hz), 133.99, 132.85, 132.77, 132.02, 130.49 (d, *J* = 2.9 Hz), 129.54, 128.38, 127.54, 126.54, 125.97, 124.68, 124.56, 118.30, 116.71, 116.53, 115.16, 102.49, 98.36, 70.69, 66.52, 60.23, 46.95, 44.36, 36.95, 33.29, 25.75, 13.67. HR‐MS (ESI) Calcd. for C_37_H_36_FN_9_O_4_ [M + H]^+^: *m/z* 690.2947, Found 690.2949.

*(R)‐1‐(2‐fluoro‐5‐((4‐oxo‐3,4‐dihydrophthalazin‐1‐yl)methyl)benzoyl)‐N‐((6‐(3‐methylmorpholino)‐2‐(1H‐pyrrolo[2,3‐b]pyridin‐4‐yl)pyrimidin‐4‐yl)methyl)piperidine‐4‐carboxamide* *(* ***B5*** *)*. White solid, yield 62%. m.p. 239.6–240.4 °C. Purity: 97.71% by analytical HPLC. ^1^H NMR (500 MHz, DMSO‐*d*_*6*_) *δ* 12.59 (s, 1H), 11.75 (s, 1H), 8.47 (t, *J* = 6.0 Hz, 1H), 8.31 (d, *J* = 4.9 Hz, 1H), 8.26 (d, *J* = 7.7 Hz, 1H), 8.01 – 7.94 (m, 2H), 7.84 (dt, *J* = 29.9, 6.7 Hz, 2H), 7.59 – 7.54 (m, 1H), 7.48 – 7.18 (m, 4H), 6.58 (d, *J* = 2.5 Hz, 1H), 4.49 (d, *J* = 12.3 Hz, 2H), 4.33 (d, *J* = 5.7 Hz, 4H), 4.11 (dd, *J* = 11.8, 7.3 Hz, 1H), 4.02 – 3.98 (m, 1H), 3.80 (d, *J* = 11.4 Hz, 1H), 3.66 (dd, *J* = 11.4, 3.1 Hz, 1H), 3.51 (td, *J* = 11.8, 3.1 Hz, 1H), 3.39 (d, *J* = 13.6 Hz, 1H), 3.25 (td, *J* = 12.9, 4.0 Hz, 1H), 3.06 (s, 1H), 2.94 – 2.82 (m, 1H), 2.62 – 2.54 (m, 1H), 1.90 (d, *J* = 12.9 Hz, 1H), 1.76 – 1.46 (m, 3H), 1.24 (dd, *J* = 6.7, 2.4 Hz, 3H). ^13^C NMR (126 MHz, DMSO‐*d*_*6*_) *δ* 174.49, 166.92, 164.20, 163.26, 162.49, 159.85, 150.67, 145.40, 142.65, 137.24, 135.31 (d, *J* = 3.0 Hz), 133.92, 132.01, 131.86 (d, *J* = 9.0 Hz), 129.54, 129.11 (d, *J* = 4.4 Hz), 128.38, 127.52, 126.55, 125.94, 124.74, 124.59, 118.30, 116.41, 116.24, 115.14, 102.52, 98.39, 70.70, 66.52, 46.98, 44.23, 42.02, 41.19, 36.89, 29.19, 28.63, 13.65. HR‐MS (ESI) Calcd. for C_39_H_38_FN_9_O_4_ [M + H]^+^: *m/z* 716.3104, Found 716.3103.

### Synthetic Procedure for Compound **B6**

To a solution of intermediate **33** (0.55 mmol) in CH_2_Cl_2_ (4 mL) at room temperature, was added trifluoroacetic acid (1.5 mL). The mixture was stirred at room temperature for 0.5 h. The reaction was monitored by TLC. Upon completion, the solvent was removed under reduced pressure to yield the corresponding amine as a colorless oil, which was used directly in the next step reaction.

To a solution of corresponding amine (0.33 mmol, crude material from previous step) and DIPEA (1 mL) in DMF (7.0 mL) were added **29** (0.43 mmol), HOBT (0.43 mmol), and EDCI (0.86 mmol). The resulting solution was stirred at room temperature for 3 h. The reaction was monitored by TLC. Upon completion, the mixture was quenched with cool water (70 mL) and extracted with EtOAc (70 mL × 3). The combined organic layers were washed with water and brine and dried over Na_2_SO_4_. The drying agent was filtered off. The filtrate was concentrated under reduced pressure, and the residue was purified via flash column chromatography (CH_2_Cl_2_/MeOH 47:3, *v/v*) to give compound **B6** as a white solid, yield 83%. *(R)‐1‐(2‐fluoro‐5‐((4‐oxo‐3,4‐dihydrophthalazin‐1‐yl)methyl)benzoyl)‐N‐((6‐(3‐methylmorpholino)‐2‐(1H‐pyrrolo[2,3‐b]pyridin‐4‐yl)pyrimidin‐4‐yl)methyl)piperidine‐4‐sulfonamide (* ***B6***). m.p. 250.5–251.6 °C. Purity: 97.37% by analytical HPLC. ^1^H NMR (500 MHz, DMSO‐*d*_*6*_) *δ* 12.58 (s, 1H), 11.77 (s, 1H), 8.32 (d, *J* = 5.0 Hz, 1H), 8.26 (dd, *J* = 7.9, 1.6 Hz, 1H), 8.02 – 7.92 (m, 2H), 7.91 – 7.78 (m, 3H), 7.57 (t, *J* = 2.9 Hz, 1H), 7.45 – 7.25 (m, 3H), 7.20 (t, *J* = 9.0 Hz, 1H), 6.81 (s, 1H), 4.53 (d, *J* = 12.5 Hz, 2H), 4.38 – 4.22 (m, 4H), 4.12 (d, *J* = 28.5 Hz, 1H), 4.07 – 4.00 (m, 1H), 3.81 (d, *J* = 11.5 Hz, 1H), 3.67 (dd, *J* = 11.8, 3.2 Hz, 1H), 3.58 – 3.36 (m, 3H), 3.29 (td, *J* = 12.8, 3.9 Hz, 1H), 2.95 (d, *J* = 13.2 Hz, 1H), 2.72 – 2.61 (m, 1H), 2.16 (d, *J* = 12.6 Hz, 1H), 1.96 (d, *J* = 5.2 Hz, 1H), 1.63 – 1.41 (m, 2H), 1.27 (d, *J* = 6.7 Hz, 3H). ^13^C NMR (126 MHz, DMSO‐*d*_*6*_) *δ* 166.15, 164.28, 163.37, 162.59, 159.85, 150.65, 145.37, 142.67, 137.12, 135.33 (d, *J* = 2.6 Hz), 133.93, 132.03, 129.54, 129.23 (d, *J* = 3.3 Hz), 128.36, 127.65, 126.55, 125.91, 124.34, 124.20, 118.24, 116.41, 116.24, 115.19, 102.41, 99.06, 70.68, 66.50, 57.88, 48.06, 47.10, 36.86, 26.65, 26.07, 13.78. HR‐MS (ESI) Calcd. for C_38_H_38_FN_9_O_5_S [M + H]^+^: *m/z* 752.2773, Found 752.2770.

### General Synthetic Procedure for Compounds **B7**–**B14**

Compounds **B7–B14** were synthesized according to the procedure described for compounds **A2–A5**.

*(R)‐4‐(2‐fluoro‐5‐((4‐oxo‐3,4‐dihydrophthalazin‐1‐yl)methyl)benzoyl)‐N‐((6‐(3‐methylmorpholino)‐2‐(1H‐pyrrolo[2,3‐b]pyridin‐4‐yl)pyrimidin‐4‐yl)methyl)piperazine‐1‐carboxamide* *(* ***B7*** *)*. White solid, yield 73%. m.p. 239.5–241.7 °C. Purity: 96.27% by analytical HPLC. ^1^H NMR (500 MHz, DMSO‐*d*_*6*_) *δ* 12.60 (s, 1H), 11.74 (s, 1H), 8.31 (d, *J* = 4.9 Hz, 1H), 8.27 (dd, *J* = 7.8, 1.4 Hz, 1H), 8.01 – 7.95 (m, 2H), 7.93 – 7.79 (m, 2H), 7.58 – 7.53 (m, 1H), 7.48 – 7.35 (m, 2H), 7.33 – 7.20 (m, 3H), 6.62 (s, 1H), 4.52 (s, 1H), 4.40 – 4.26 (m, 4H), 4.11 (d, *J* = 13.1 Hz, 1H), 4.01 (dd, *J* = 11.3, 3.8 Hz, 1H), 3.80 (d, *J* = 11.4 Hz, 1H), 3.73 – 3.60 (m, 3H), 3.58 – 3.45 (m, 3H), 3.35 (d,*J* = 3.2 Hz, 2H), 3.23 (dq, *J* = 24.4, 5.5, 4.7 Hz, 3H), 1.25 (d, *J* = 6.8 Hz, 3H). ^13^C NMR (126 MHz, DMSO‐*d*_*6*_) *δ* 168.15, 164.55, 163.11, 162.46, 159.88, 157.82, 150.67, 145.36, 142.66, 137.37, 135.32 (d, *J* = 3.2 Hz), 133.97, 132.19 (d, *J* = 7.2 Hz), 132.05, 129.56, 129.44 (d, *J* = 3.5 Hz), 128.38, 127.44, 126.56, 125.93, 124.24, 124.10, 118.33, 116.51, 116.33, 115.12, 102.57, 98.38, 70.75, 66.54, 46.92, 45.81, 44.16, 43.75, 41.88, 36.91, 13.63. HR‐MS (ESI) Calcd. for C_38_H_37_FN_10_O_4_ [M + H]^+^: *m/z* 717.3056, Found 717.3050.

*(R)‐4‐(4‐(2‐fluoro‐5‐((4‐oxo‐3,4‐dihydrophthalazin‐1‐yl)methyl)benzoyl)piperazine‐1‐carbonyl)‐N‐((6‐(3‐methylmorpholino)‐2‐(1H‐pyrrolo[2,3‐b]pyridin‐4‐yl)pyrimidin‐4‐yl)methyl) benzamide* *(* ***B8*** *)*. White solid, yield 74%. m.p. 254.6‐258.1 °C. Purity: 98.27% by analytical HPLC. ^1^H NMR (500 MHz, DMSO‐*d*_*6*_) *δ* 12.60 (s, 1H), 11.73 (s, 1H), 9.21 (t, *J* = 5.9 Hz, 1H), 8.36 – 8.17 (m, 2H), 8.11 – 7.67 (m, 6H), 7.64 – 7.12 (m, 7H), 6.71 (s, 1H), 4.57 (d, *J* = 6.0 Hz, 3H), 4.34 (s, 2H), 4.05 (dd, *J* = 62.0, 12.1 Hz, 2H), 3.85 – 3.39 (m, 8H), 3.25 (td, *J* = 12.8, 4.0 Hz, 4H), 1.25 (d, *J* = 6.7 Hz, 3H). ^13^C NMR (126 MHz, DMSO‐*d*_*6*_) *δ* 169.08, 166.77, 166.49, 164.57, 163.28, 162.50, 159.88, 157.85, 155.90, 150.66, 145.30, 142.67, 138.72, 137.25, 135.69, 135.32 (d, *J* = 2.8 Hz), 133.93, 132.27, 131.99, 129.56, 128.38, 128.04, 127.54, 127.46, 126.56, 125.93, 118.31, 116.53, 116.36, 115.11, 102.57, 98.73, 70.72, 66.52, 46.93, 45.02, 36.92, 13.69. HR‐MS (ESI) Calcd. for C_45_H_41_FN_10_O_5_ [M + Na] ^+^: *m/z* 843.3138, Found 843.3123.

*(R)‐4‐(4‐(2‐fluoro‐5‐((4‐oxo‐3,4‐dihydrophthalazin‐1‐yl)methyl)benzoyl)piperazine‐1‐carbonyl)‐N‐((6‐(3‐methylmorpholino)‐2‐(1H‐pyrrolo[2,3‐b]pyridin‐4‐yl)pyrimidin‐4‐yl)methyl) benzenesulfonamide* *(* ***B9*** *)*. White solid, yield 61%. m.p. 242.6‐243.7 °C. Purity: 98.26% by analytical HPLC. ^1^H NMR (500 MHz, DMSO‐*d*_*6*_) *δ* 12.60 (s, 1H), 11.73 (s, 1H), 8.48 (t, *J* = 6.3 Hz, 1H), 8.28 (d, *J* = 22.7 Hz, 2H), 7.89 (t, *J* = 29.7 Hz, 6H), 7.66 – 7.14 (m, 7H), 6.65 (s, 1H), 4.34 (s, 3H), 4.08 (dd, *J* = 83.2, 8.7 Hz, 4H), 3.86 – 3.44 (m, 7H), 3.14 (d, *J* = 88.7 Hz, 5H), 1.23 (d, *J* = 5.6 Hz, 3H). ^13^C NMR (126 MHz, DMSO‐*d*_*6*_) *δ* 168.30, 164.84, 164.52, 163.24, 162.79, 162.33, 159.88, 150.60, 145.30, 142.59, 142.12, 139.68, 136.98, 135.34 (d, *J* = 3.6 Hz), 133.96, 132.35, 132.02, 129.56, 128.39, 128.14, 127.60, 127.27, 126.56, 125.93, 118.18, 116.51, 116.34, 115.18, 102.38, 99.26, 70.64, 66.47, 48.08, 47.01, 36.91, 13.78. HR‐MS (ESI) Calcd. for C_44_H_41_FN_10_O_6_S [M + H] ^+^: *m/z* 857.2988, Found 857.2977.

*(R)‐6‐(4‐(2‐fluoro‐5‐((4‐oxo‐3,4‐dihydrophthalazin‐1‐yl)methyl)benzoyl)piperazine‐1‐carbonyl)‐N‐((6‐(3‐methylmorpholino)‐2‐(1H‐pyrrolo[2,3‐b]pyridin‐4‐yl)pyrimidin‐4‐yl)methyl) nicotinamide* *(* ***B10*** *)*. White solid, yield 68%. m.p. 239.6–240.9 °C. Purity: 98.37% by analytical HPLC. ^1^H NMR (500 MHz, DMSO‐*d*_*6*_) *δ* 12.59 (d, *J* = 18.7 Hz, 1H), 11.74 (d, *J* = 5.2 Hz, 1H), 9.41 (t, *J* = 5.9 Hz, 1H), 9.11 (dd, *J* = 18.9, 2.2 Hz, 1H), 8.43 (dt, *J* = 7.9, 2.6 Hz, 1H), 8.37 – 8.19 (m, 2H), 8.06 – 7.69 (m, 5H), 7.56 – 7.32 (m, 3H), 7.32 – 7.17 (m, 2H), 6.75 (d, *J* = 6.4 Hz, 1H), 4.59 (d, *J* = 6.0 Hz, 3H), 4.33 (d, *J* = 20.4 Hz, 2H), 4.12 (d, *J* = 17.6 Hz, 1H), 4.01 – 3.94 (m, 1H), 3.87 – 3.57 (m, 6H), 3.57 – 3.44 (m, 2H), 3.32 – 3.14 (m, 3H), 1.25 (t, *J* = 7.4 Hz, 3H). ^13^C NMR (126 MHz, DMSO‐*d*_*6*_) *δ* 166.90, 166.82, 166.41, 165.15 (d, *J* = 4.4 Hz), 164.59 (d, *J* = 7.0 Hz), 163.31, 162.52, 159.89, 156.08, 155.92, 150.65, 148.01 (d, *J* = 8.5 Hz), 145.31 (d, *J* = 6.0 Hz), 142.68, 137.24, 136.99, 136.92, 135.27, 133.97, 133.86, 132.04, 131.90, 129.53, 128.38, 127.46, 126.55, 125.92, 123.52, 118.31, 116.53, 116.35, 115.12, 102.54, 98.84, 70.72, 66.53, 46.92, 46.66, 44.98, 36.94, 13.70. HR‐MS (ESI) Calcd. for C_44_H_40_FN_11_O_5_ [M + H]^+^: *m/z* 822.3271, Found 822.3267.

*(R)‐5‐(4‐(2‐fluoro‐5‐((4‐oxo‐3,4‐dihydrophthalazin‐1‐yl)methyl)benzoyl)piperazine‐1‐carbonyl)‐N‐((6‐(3‐methylmorpholino)‐2‐(1H‐pyrrolo[2,3‐b]pyridin‐4‐yl)pyrimidin‐4‐yl)methyl) picolinamide* *(* ***B11*** *)*. White solid, yield 69%. m.p. 229.6–232.4 °C. Purity: 95.84% by analytical HPLC. ^1^H NMR (500 MHz, DMSO‐*d*_*6*_) *δ* 12.60 (s, 1H), 11.74 (s, 1H), 9.49 (t, *J* = 6.0 Hz, 1H), 8.77 (d, *J* = 11.1 Hz, 1H), 8.39 – 7.69 (m, 8H), 7.57 – 7.17 (m, 5H), 6.73 (s, 1H), 4.48 (dd, *J* = 139.9, 9.6 Hz, 5H), 4.10 (d, *J* = 8.7 Hz, 1H), 3.98 (d, *J* = 11.3 Hz, 1H), 3.86 – 3.39 (m, 9H), 3.29 –3.09 (m, 3H), 1.24 (d, *J* = 6.7 Hz, 3H). HR‐MS (ESI) Calcd. for C_44_H_40_FN_11_O_5_ [M + Na]^+^: *m/z* 844.3090, Found 844.3089.

*(R)‐4‐((4‐(2‐fluoro‐5‐((4‐oxo‐3,4‐dihydrophthalazin‐1‐yl)methyl)benzoyl)piperazin‐1‐yl)methyl)‐N‐((6‐(3‐methylmorpholino)‐2‐(1H‐pyrrolo[2,3‐b]pyridin‐4‐yl)pyrimidin‐4‐yl)methy l)benzamide* *(* ***B12*** *)*. White solid, yield 70%. m.p. 234.2–235.9 °C. Purity: 97.07% by analytical HPLC. ^1^H NMR (500 MHz, DMSO‐*d*_*6*_) *δ* 12.60 (s, 1H), 11.73 (s, 1H), 9.07 (t, *J* = 6.0 Hz, 1H), 8.39 – 8.20 (m, 2H), 8.04 – 7.74 (m, 6H), 7.54 – 7.39 (m, 4H), 7.35 – 7.18 (m, 3H), 6.70 (s, 1H), 4.54 (d, *J* = 6.2 Hz, 3H), 4.33 (s, 2H), 4.10 (d, *J* = 12.8 Hz, 1H), 3.99 (dd, *J* = 11.4, 3.7 Hz, 1H), 3.78 (d, *J* = 11.4 Hz, 1H), 3.71 – 3.46 (m, 6H), 3.29 – 3.11 (m, 3H), 2.42 (t, *J* = 5.1 Hz, 2H), 2.27 (s, 2H), 1.24 (d, *J* = 6.7 Hz, 3H). ^13^C NMR (126 MHz, DMSO‐*d*_*6*_) *δ* 166.97, 164.23, 163.25, 162.50, 159.87, 157.79, 155.85, 150.66, 145.36, 142.66, 137.27, 135.29 (d, *J* = 1.8 Hz), 133.95, 132.02, 129.54, 129.24, 128.38, 127.86, 127.44, 126.56, 125.95, 118.31, 116.48, 116.30, 115.11, 102.58, 98.68, 70.72, 66.52, 61.78, 53.16, 52.66, 46.92, 44.97, 36.88, 13.67. HR‐MS (ESI) Calcd. for C_45_H_43_FN_10_O_4_ [M + H] ^+^: *m/z* 829.3345, Found 829.3332.

*(R)‐4‐(4‐(2‐fluoro‐5‐((4‐oxo‐3,4‐dihydrophthalazin‐1‐yl)methyl)benzoyl)piperazin‐1‐yl)‐N‐((6‐(3‐methylmorpholino)‐2‐(1H‐pyrrolo[2,3‐b]pyridin‐4‐yl)pyrimidin‐4‐yl)methyl)benzamide* *(* ***B13*** *)*. White solid, yield 74%. m.p. 264.5‐264.9 °C. Purity: 95.17% by analytical HPLC. ^1^H NMR (500 MHz, DMSO‐*d*_*6*_) *δ* 12.60 (s, 1H), 11.73 (s, 1H), 8.86 (t, *J* = 5.9 Hz, 1H), 8.40 – 8.22 (m, 2H), 8.02 – 7.80 (m, 6H), 7.54 – 7.50 (m, 1H), 7.46 (ddd, *J* = 8.0, 5.0, 2.3 Hz, 1H), 7.39 (dd, *J* = 6.5, 2.3 Hz, 1H), 7.32 – 7.22 (m, 2H), 7.01 (d, *J* = 8.9 Hz, 2H), 6.66 (s, 1H), 4.52 (d, *J* = 6.2 Hz, 3H), 4.35 (s, 2H), 4.10 (q, *J* = 5.2 Hz, 1H), 3.99 (dd, *J* = 11.5, 3.7 Hz, 1H), 3.78 (d, *J* = 11.6 Hz, 3H), 3.65 (dd, *J* = 11.6, 3.2 Hz, 1H), 3.50 (td, *J* = 11.7, 3.0 Hz, 1H), 3.41 – 3.36 (m, 2H), 3.32 (d, *J* = 4.5 Hz, 2H), 3.28 – 3.18 (m, 3H), 1.24 (d, *J* = 6.7 Hz, 3H). ^13^C NMR (126 MHz, DMSO‐*d*_*6*_) *δ* 167.35, 166.70, 164.37, 163.22, 162.49, 159.89, 157.87, 155.92, 152.93, 150.66, 145.35, 142.66, 137.29, 135.34, 134.00, 132.23 (d, *J* = 7.0 Hz), 132.06, 129.57, 129.44, 129.18, 128.39, 127.47, 126.56, 125.94, 124.51, 124.19, 124.05, 118.32, 116.51, 116.34, 115.12, 114.60, 102.58, 98.56, 70.72, 66.51, 60.23, 47.92, 47.59, 46.93, 46.55, 44.89, 41.54, 36.91, 13.65. HR‐MS (ESI) Calcd. for C_44_H_41_FN_10_O_4_ [M + H]^+^: *m/z* 793.3369, Found 793.3369.

*(R)‐2‐(4‐(2‐fluoro‐5‐((4‐oxo‐3,4‐dihydrophthalazin‐1‐yl)methyl)benzoyl)piperazin‐1‐yl)‐N‐((6‐(3‐methylmorpholino)‐2‐(1H‐pyrrolo[2,3‐b]pyridin‐4‐yl)pyrimidin‐4‐yl)methyl)pyramidine‐5‐carboxamide* *(* ***B14*** *)*. White solid, yield 71%. m.p. 259.1‐261.2 °C. Purity: 99.30% by analytical HPLC. ^1^H NMR (500 MHz, DMSO‐*d*_*6*_) *δ* 12.61 (s, 1H), 11.74 (s, 1H), 9.03 (t, *J* = 5.9 Hz, 1H), 8.91 (s, 2H), 8.38 – 8.23 (m, 2H), 8.03 – 7.96 (m, 2H), 7.91 (td, *J* = 8.1, 7.7, 1.5 Hz, 1H), 7.84 (td, *J* = 7.5, 1.2 Hz, 1H), 7.52 (t, *J* = 3.0 Hz, 1H), 7.46 (ddd, *J* = 8.2, 5.2, 2.3 Hz, 1H), 7.41 (dd, *J* = 6.4, 2.3 Hz, 1H), 7.32 – 7.22 (m, 2H), 6.69 (s, 1H), 4.54 (d, *J* = 6.3 Hz, 3H), 4.35 (s, 2H), 4.12 (d, *J* = 13.2 Hz, 1H), 4.02 – 3.89 (m, 3H), 3.84 – 3.69 (m, 5H), 3.65 (dd, *J* = 11.5, 3.2 Hz, 1H), 3.50 (td, *J* = 11.8, 3.1 Hz, 1H), 3.31 (t, *J* = 5.2 Hz, 2H), 3.24 (td, *J* = 12.9, 3.9 Hz, 1H), 1.25 (d, *J* = 6.7 Hz, 3H). ^13^C NMR (126 MHz, DMSO‐*d*_*6*_) *δ* 166.85, 164.60, 164.40, 163.26, 162.53, 161.90, 159.88, 158.30, 157.88, 155.93, 150.66, 145.34, 142.68, 137.25, 135.32, 133.98, 132.30, 132.23, 132.05, 129.58, 129.52 (d, *J* = 3.5 Hz), 128.38, 127.49, 126.57, 125.93, 124.20, 124.05, 118.30, 116.94, 116.53, 116.36, 115.12, 102.50, 98.63, 70.73, 66.54, 60.23, 46.90, 46.64, 44.60, 44.09, 43.59, 41.69, 36.91, 13.70. HR‐MS (ESI) Calcd. for C_42_H_39_FN_12_O_4_ [M + H] ^+^: *m/z* 795.3274, Found 795.3274.

### Pharmacology—Bioinformatics Analysis

ATR‐related and PARP1‐related PPI network was predicted by “The cancer genome atlas (TCGA)” database. Then, function annotation of ATR‐related and PARP1‐related proteins was performed using DAVID (https://david.ncifcrf.gov/), and the proteins with the functions of cell cycle and apoptosis were filtered out. Finally, CBioPortal (https://www.cbioportal.org/) and an online cancer genomic database (http://gepia2.cancer‐pku.cn/#index) were used for correlation analysis of ATR and PARP1 in BRAC (Breast invasive carcinoma). PPI networks were produced by using “Strings (https://cn.string‐db.org/)” and “Cytoscape (https://cytoscape.org/)”.

### Molecular Docking

The protein structures of mimetic ATR and PARP1 were retrieved from the RCSB Protein Data Bank (https://www.rcsb.org/) using PDB IDs 5UL1 and 5DS3, respectively. These structures were imported into Discovery Studio, where crystallographic water molecules and co‐crystallized ligands were removed. The proteins were then prepared by adding hydrogen atoms, assigning partial charges, and performing energy minimization to ensure structural stability. The inhibitor (ligand) was optimized separately through energy minimization and saved in a format compatible with the docking software. The binding site on the protein was defined, and docking parameters, such as grid box size and resolution, were configured. Docking simulations were performed, generating multiple binding poses for the ligand. The top‐scoring pose was selected, and the results were validated by re‐docking with a reference ligand.

### ATR Inhibition Assays

The in vitro ATR kinase inhibitory activities were determined by ChemPartner Co., Ltd. (Shanghai, China) using the Mobility Shift Assay. The tested compound was dissolved with DMSO and diluted to 1000/3 × of the final desired highest inhibitor concentration in the reaction. A fixed volume of each compound (0.06 µL) was transferred to a 384‐well assay plate by Echo liquid handler (Beckman Coulter). The 2 × enzyme solution with 10 nM ATR kinase was prepared by ATR/ATRIP protein complex (Eurofins, #14‐953) and 1 × kinase base buffer (50 mM HEPES pH 7.5, 0.0015% Brij‐35, 1 M MnCl_2_), and the 2 × substrate solution with 6 µM 5‐FAM‐AK‐17 and 4 µM ATP was prepared by 5‐FAM‐AK‐17 (GL, #524 315), ATP and 1 × kinase base buffer. The 2 × enzyme solution (10 µL) was added to the above 384‐well plate and incubated for 10 min at room temperature, and then 10 µL of 2 X substrate solution was added to each well of the 384‐well plate. The assay wells were incubated at 28 °C for 4 h. Finally, 30 µL of stop buffer (100 mM HEPES pH 7.5, 0.015% Brij‐35, 0.2% Coating Reagent #3, 50 mM EDTA) was added to stop the reaction. The 384‐well plate was put into the Caliper EZ Reader (Caliper Life Sciences) to read the conversion data.

### PARP Inhibition Assays

The inhibition of the tested compounds on PARP enzymatic activity was determined by enzyme‐linked immunosorbent assay (ELISA) in 384‐well plates. The PARP activity assay was performed by ChemPartner (Shanghai, China). Each well was precoated with histone (final conc.: 100 ng mL^−1^) diluted in PBS buffer (contains 10 mmol L^−1^ NaH_2_PO_4_ and 150 mmol L^−1^ NaCl, pH 7.4) by incubation at 4 °C overnight. NAD^+^ (8.75 µM) and sDNA (2 nM) in reaction buffer (contains 0.005% Tween‐20, 0.01% BSA, and 50 mM Tris, pH 7.5) were added 10 µL into each well, and then 5 µL of solvent control or compound was added at varying concentrations. The reaction was initiated by the addition of 10 µL of PARP (final conc.: 0.007 nM/well) at room temperature for 60 min. Add 20 µL of anti‐Poly/Mono‐ADP Ribose Rabbit mAb. Incubate for 1.5 h at room temperature and wash the plate three times using PBST buffer. Add 20 µL of diluted (1:2000) anti‐rabbit IgG, HRP‐linked Antibody. Incubate for 1 h at room temperature and wash the plate three times using PBST buffer. Add 25 µL Femto‐ECL Substrate A and Femto‐ECL Substrate B (1:1) mix. Immediately read chemiluminescence on Envision (Revvity, USA). The concentration required for 50% inhibition of PARP enzymatic activity (IC_50_) was calculated using nonlinear regression with normalized dose response fit using Prism GraphPad 9.0 software.

### Cell Lines and Culture Methods

MDA‐MB‐231, MDA‐MB‐468, and MDA‐MB‐436 were obtained from the American Type Culture Collection (ATCC) or the Cell Bank of the Chinese Academy of Sciences (CCAS, China). MDA‐MB‐231, MDA‐MB‐468 and MDA‐MB‐436 were cultured in DMEM supplemented with 10% (*v/v*) FBS and 1% (*v/v*) penicillin−streptomycin. All cells were grown in a humidified incubator at 37 °C and 5% CO_2_.

### Cell Growth Inhibition Assays

Cells were grown at 37 °C, under 95% air and 5% CO₂ until they reached about 70% confluence and were subcultured at least twice before the experiment. Cells were seeded at a density of 1000 cells/well in two 384‐well white plates, and the plates were placed in a 5% CO₂ incubator overnight. Growth viability was determined by the CellTiter‐Glo luminescent viability assay (Promega) 5 days after drug treatment. Cell viability was measured immediately after dosing (Day 0) and after 5 days of incubation using CellTiter‐Glo (CTG, Promega, G7573). Relative viability was calculated by normalizing the raw luminescence counts of the treated groups to those of the DMSO control groups. IC₅₀ values were calculated using sigmoidal dose‐response curve fitting with GraphPad Prism 9.0 software.

### Cell Cycle Assays

The cell cycle analysis was performed with cells seeded in 6‐well plates in growth media. The cells were allowed to attach overnight (8 h). The cultures were treated with different tested drugs for 48 h. After treatment, cells were collected and fixed with 70% pre‐cold ethanol in PBS and stored at −20 °C overnight. Then washed the cells with PBS twice, and incubated with 100 µg mL^−1^ RNase A at 37 °C for 1 h, stained with propidium iodide for 30 min avoid light at room temperature. The stained cells were analyzed by flow cytometry. The percentages of cells in the G1, S, and G2/M phases were determined. The data were analyzed by FlowJo software (Tree Star, Inc., Ashland, OR).

### Cell Apoptosis Assays

The cell apoptosis analysis was performed with cells seeded in 6‐well plates in growth media. The cells were allowed to attach overnight (8 h). The cultures were treated with different tested drugs for 72 h and evaluated using an Annexin V Flow Kit (BioLegend#B367750) according to the manufacturer's instructions. The data were analyzed by FlowJo software (Tree Star, Inc., Ashland, OR).

### Colony Formation Assays

The colony formation assay was performed with cells seeded in 6‐well plates (10 000 cells/well). After 10 days, cells were fixed with methanol and stained with crystal violet. The number of colonies was counted. Data represent the means ± SD from 3 independent experiments performed in triplicate wells.

### Cell Migration assay and Transwell Assays

For the wound‐healing assay, MDA‐MB‐231 cells were seeded into 6‐well plates and grown to 80% confluence. A scratch was made in the cell monolayer using a 20 µL pipette tip, and the cells were washed with PBS to remove floating cells and debris. The cells were then incubated in serum‐free medium. Cells are divided into control and drug treatment Cells in the scratched area were imaged at 0 and 48 h using microscopy, and the distance traveled by cells at the leading edge of the wound at each time point was measured. To assess cell migration, a transwell assay was performed. Briefly, MDA‐MB‐231 or MDA‐MB‐468 cells were suspended in DMEM at a concentration of 1 × 10^5^ cells/mL and seeded into the upper chamber of a 24‐well transwell plate. Following 24 h of incubation, the filters were fixed in 4% paraformaldehyde, stained with 0.1% crystal violet, and then imaged and counted under a microscope to determine the number of invading cells. To assess cell invasion, the transwell plate was coated with a layer of Matrigel, and the rest procedure is similar to what has been described above.

### Immunofluorescence

Cells were fixed for 30 min in 4% paraformaldehyde and permeabilized for 30 min in 1% Triton X‐100 at 4 °C. After three rinses with PBS, cells were blocked for 30 min with 5% BSA and incubated with *γ*H2AX antibody (1/1000 dilution, CST#9718) in 5% BSA overnight at 4 °C, after which they were rinsed three times with PBS and incubated with the secondary antibodies at room temperature for 1 h. Then, cells were washed with PBS three times, and the nucleus was stained with DAPI and fixed. Finally, images were visualized with the microscope and analyzed.

### Western Blotting Analysis

Cells were washed with cold PBS and lysed in RIPA buffer containing protease inhibitors (MCE, HY‐K0010). The lysate was centrifuged (13 000 rpm, 4 °C, 15 min); the protein concentrations were determined by the BCA Assay Kit (Beyotime, p0012s). Proteins were loaded onto 10% SDS‐PAGE gel and then transferred onto polyvinylidene fluoride (Millipore) membranes. The membranes were blocked for 1 h, incubated with primary antibodies overnight at 4 °C, and then washed three times with Tris‐buffered saline with Tween 20 (TBST) for 10 min. After incubating with the secondary HRP antibody for 1 h at room temperature, the membranes were washed three times with TBST for 10 min and then exposed on autoradiograph films using enhanced chemiluminescence. The primary antibodies used were ATR (1/1000 dilution, CST#13 934), PARP1 (1/1000 dilution, CST#9532), BCL‐2 (1/1000 dilution, CST#4223), Bax (1/1000 dilution, CST#5023), Caspase 3 (1/1000 dilution, CT#9662), Cleaved caspase 3 (1/1000 dilution, CST#9661), E‐cadherin (1/1000 dilution, CST#3195), Vimetin (1/1000 dilution, CST#5741), p‐CHK1 (1/1000 dilution, CST#2348), CHK1 (1/1000 dilution, CST#2360), CDK1 (Proteintech#19532‐1‐AP), p‐CDK1 (CST#9114), *γ*H2AX (1/1000 dilution, CST#9718), and β‐Actin (1/1000 dilution, Proteintech# 66009‐1‐Ig).

### MDA‐MB‐468 Xenografted Mice Model

Anticancer efficacy experiment in MDA‐MB‐468 Xenografted mice model was performed by Shanghai Medicilon Inc. All animal experiments in this study were conducted in strict accordance with national animal welfare regulations and were approved by the institutional animal ethics committee (Approval number: IACUC‐HZN2401P; Approval Date: 2024‐10‐18). MDA‐MB‐468 cells (1 × 10^7^) were implanted in the right flanks subcutaneously in female nude mice. When the implanted tumor grew to a volume of 110 mm^3^, the animals were randomly divided into four groups, each group containing four animals. Compound **B8** was intraperitoneally (IP) administered at two different doses, bis in die (BID) for 28 days: low dose: 25 mg k^−1^g and high dose 50 mg k^−1^g. In the positive control group, the combination (AZD6738 + Olaparib at a dose of 25 mg k^−1^g each) was administrated bis in die for the first 7 days and then switched to once daily (qd) for the rest of 21 days due to observation of significant weight loss (>15%) and animal loss (possibly due to the toxicity issue). Tumor size and body weights were measured twice a week. On day 28, the mice were euthanized, tumors were harvested and weighed, and vital organs (heart, liver, spleen, lung, and kidney) were collected. Processed samples were saved for further safety evaluation.

### Hematoxylin and Eosin (H&E) Staining

The tumor sample and vital organs were immersed in 4% paraformaldehyde for 4 h and transferred to 70% ethanol. Individual lobes of the sample were placed in processing cassettes, dehydrated through a serial alcohol gradient, and embedded in paraffin wax blocks. Thick tissue sections (5 µm) were dewaxed in xylene, rehydrated through decreasing concentrations of ethanol, washed in PBS, and stained with H&E. After staining, the sections were dehydrated through increasing concentrations of ethanol and xylene. Images were acquired on a fluorescent inverted microscope (Nikon DS‐Ri1).

### Immunohistochemistry Staining

The isolated tumor tissue was fixed in 4% paraformaldehyde for 24 h, dehydrated, embedded, and sliced. After deparaffinization, Ki67 (GB121141‐50, Servicebio), *γ*H2AX (GB111841‐50, Servicebio), CD8 (GB15068‐50, Servicebio), or Cleaved‐caspase‐3 antibody (CST#9661) were incubated at 4 °C overnight, rinsed three times with PBS, and incubated with the secondary antibody. A DAB substrate solution was applied to the slide sections to reveal the antibody staining color. The tissue slide was dehydrated four times with alcohol. The tissue slides were then cleared three times in xylene and mounted with a mounting solution. The mounted slides could be stored at room temperature indefinitely. The immunohistochemical images were obtained under the microscope (Nikon DS‐Ri1).

### Statistical Analyses

The presented data and results were confirmed in at least three independent experiments. The data were expressed as means ± SD and analyzed with GraphPad Prism 9.0 software. P values were determined using an unpaired t‐test, one‐way ANOVA, or two‐way ANOVA test. Ns: No significant, *p < 0.05, **p < 0.01, ***p < 0.001, ****p < 0.0001.

## Conflict of Interest

All authors declare no competing interests.

## Author Contributions

Y.G. designed and executed experiments, analyzed and interpreted data, and wrote the original manuscript. J.Z., C.C.W., Z.H.W., N.D.M., M.L.H., and P.P.Z. performed the study experiment and design. P.H., G.W.Y., Y.Q.Z., and F.H.T. provided acquisition, analysis, and interpretation of data, and statistical analysis. H.Z., M.H.G., and T.X. reviewed and revised the paper. X.Y.Y. provided research funding, conceived and supervised the project, and edited the manuscript. All authors read and approved the final paper.

## Supporting information



Supporting Information

Supporting Information

## Data Availability

The data that support the findings of this study are available from the corresponding author upon reasonable request.
