# Supplementary material for: Novel ATR/PARP1 Dual Inhibitors Demonstrate Synergistic Antitumor Efficacy in Triple‐Negative Breast Cancer Models
Source: Adv Sci (Weinh). 2025 Jun 16;12(29):e01916. doi: 10.1002/advs.202501916 (PMC12362783; doi:10.1002/advs.202501916)
Supplement: Supplementary file 1 — Supporting Information [file ADVS-12-e01916-s001.docx]

**Supporting Information**

**Novel ATR/PARP1 Dual Inhibitors Demonstrate Synergistic Antitumor Efficacy in Triple-Negative Breast Cancer Models**

Yuan Gao*^a,b^*, Jiawei Zhou*^b,c^*, Chen-Chen Wang*^d^*, Zong-Hao Wang*^b,c^*, Nian-Dong Mao*^b,d^*, Meng-Lan He*^b,c^*, Peng-Peng Zhang*^b,c^*, Ping Huang*^e^*, Guo-Wei Ye*^b,c^*, Yu-Qing Zhang*^b,c^*, Feng-Hui Tang*^b,c^*, Hang Zhang*^f^*^,*^, Minghua Ge*^g^*^,*^, Tian Xie*^a,b,c,*^*, and Xiang-Yang Ye*^b,c,*^*

*^a^* Clinical Research Institute, Zhejiang Provincial People’ s Hospital (Affiliated People’ s Hospital), Hangzhou Medical College, Hangzhou, Zhejiang Province 310000, China

*^b^* School of Pharmacy, Hangzhou Normal University, Hangzhou, Zhejiang Province 310000, China

*^c^* Key Laboratory of Elemene Class Anti-Cancer Chinese Medicines; Engineering Laboratory of Development and Application of Traditional Chinese Medicines; Collaborative Innovation Center of Traditional Chinese Medicines of Zhejiang Province, Hangzhou Normal University, Hangzhou, Zhejiang Province 310000, China.

*^d^* College of Life and Environmental Sciences, Hangzhou Normal University, Hangzhou, Zhejiang Province 310000, China

*^e^* Department of Pharmacy, Zhejiang Provincial People’s Hospital (Affiliated People’ s Hospital), Hangzhou Medical College, Hangzhou, Zhejiang Province 310000, China

*^f^* School of Basic Medical Science, Hangzhou Normal University, Hangzhou, Zhejiang Province 310000, China

*^g^* Department of Head and Neck Surgery, Zhejiang Provincial People’s Hospital (Affiliated People’ s Hospital), Hangzhou Medical College, Hangzhou, Zhejiang Province 310000, China

***Corresponding Authors**

**Xiang-Yang Ye** – School of Pharmacy, Hangzhou Normal University, Hangzhou, Zhejiang 311121, PR China; Key Laboratory of Elemene Class Anti-Cancer Chinese Medicines; Engineering Laboratory of Development and Application of Traditional Chinese Medicines; Collaborative Innovation Center of Traditional Chinese Medicines of Zhejiang Province, Hangzhou Normal University, Hangzhou, Zhejiang 311121, P.R. China; E-mails: xyye@hznu.edu.cn; orcid.org/0000-0003-3739-0930

**Tian Xie** – Clinical Research Institute, Zhejiang Provincial People’ s Hospital (Affiliated People’ s Hospital), Hangzhou Medical College, Hangzhou, Zhejiang Province 310000, PR China; School of Pharmacy, Hangzhou Normal University, Hangzhou, Zhejiang 311121, PR China; Key Laboratory of Elemene Class Anti-Cancer Chinese Medicines; Engineering Laboratory of Development and Application of Traditional Chinese Medicines; Collaborative Innovation Center of Traditional Chinese Medicines of Zhejiang Province, Hangzhou Normal University, Hangzhou, Zhejiang 311121, P.R. China; E-mails: tianxie@hznu.edu.cn; orcid.org/0000-0001-7066-1443

**Minghua Ge** – Department of Head and Neck Surgery, Zhejiang Provincial People’s Hospital (Affiliated People’ s Hospital), Hangzhou Medical College, Hangzhou, Zhejiang Province 310000, PR China; E-mails: geminghua@hmc.edu.cn

**Hang Zhang** – School of Basic Medical Science, Hangzhou Normal University, Hangzhou, Zhejiang 311121, PR China; email: 20090096@hznu.edu.cn; ORCID: 0009-0007-4558-5582

## Table of Content

## Part 1: The correlation between the mRNA expression of ATR and PARP1 in other cancer S4

## Part 2: Synthesis of intermediates S5

## Part 3: ^1^H NMR and ^13^C NMR spectra for compounds A1-A12 and B1-B14 S13

**Part 4: HPLC spectra for compounds A1-A12 and B1-B14 S39**

**Part 5: Anti-proliferative effects of the drugs at different time points S64**

**Part 6: Representative images of transwell invasion assay in MDA-MB-468 cells S64**

**Part 7: Relative densitometric values S65**

**Part 8: Molecular docking of compound B8 S65**

## Part 1: The correlation between the mRNA expression of ATR and PARP1 in other cancer

**
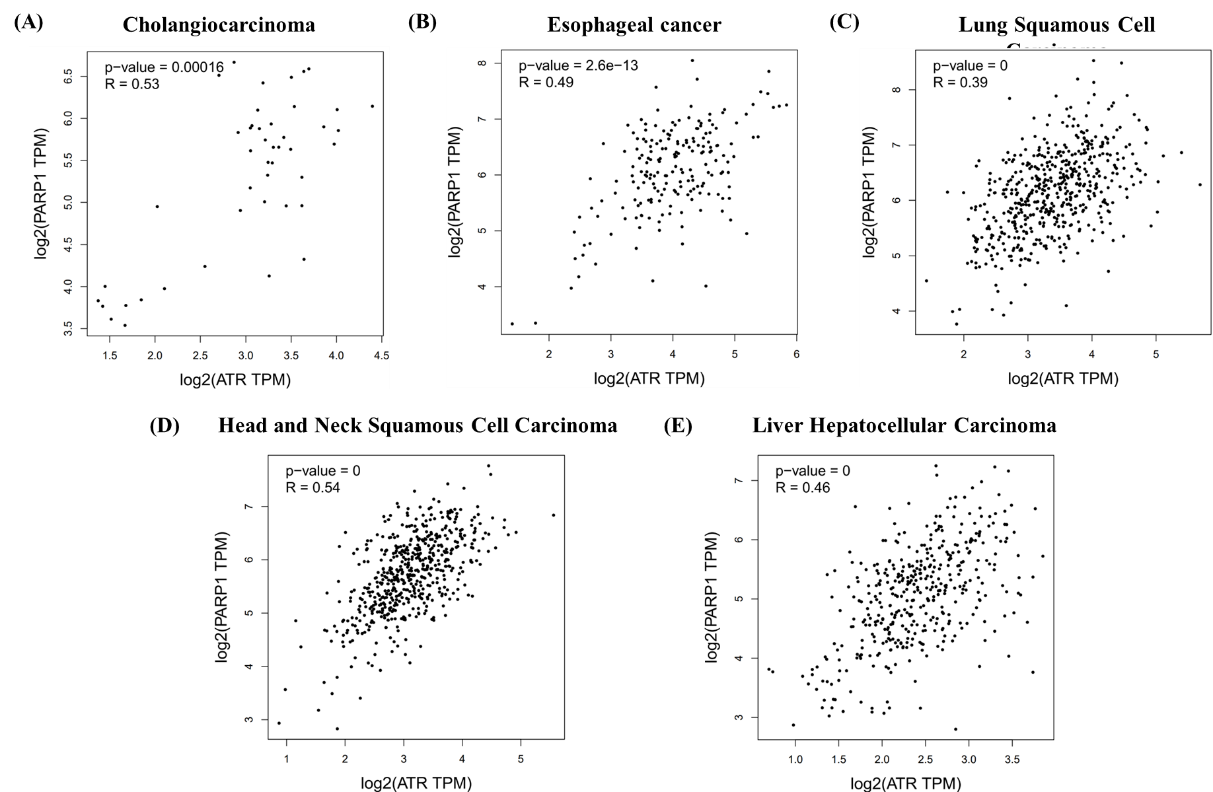
**

**Figure S1.** A positive correlation between the mRNA expression of ATR and PARP1 in (A) 36 cancer samples + 9 normal samples; (B) 182 cancer samples + 13 normal samples; (C) 486 cancer samples + 50 normal samples; (D) 519 cancer samples + 44 normal samples and (E) 369 cancer samples + 50 normal sample.

**Part 2 Synthesis of intermediates**

Synthesis of intermediate **15**. *(R)-4-(2,6-dichloropyrimidin-4-yl)-3-methylmorpholine* (**15**). To a solution of **14** (0.68 g, 6.7 mmol) and Et_3_N (1.42 mL, 10.1 mmol) in EtOH (10 mL) at room temperature was added **13** (1.23 g, 6.73 mmol). The mixture was stirred room temperature for 16 h. The reaction was monitored by TLC. Upon completion, the mixture was quenched with cool water (50 mL) at room temperature and extracted with EtOAc (50 mL × 3). The combined organic layers were washed with water and brine, and dried over Na_2_SO_4_. The filtrate was concentrated under reduced pressure, and the residue was purified via flash column chromatography (PE/EtOAc = 10:1, v/v) to afford intermediate **15** (1.1 g, yield 66%) as a white solid. ^1^H NMR (500 MHz, CDCl_3_-*d*) δ 6.37 (s, 1H), 4.69 – 3.83 (m, 3H), 3.82 – 3.74 (m, 1H), 3.67 (dd, *J* = 11.7, 3.3 Hz, 1H), 3.58 – 3.48 (m, 1H), 3.36 – 3.20 (m, 1H), 1.32 (d, *J* = 6.9 Hz, 3H).

Synthesis of intermediate **17**. *(R)-1-(2-chloro-6-(3-methylmorpholino)pyrimidin-4-yl)cyclopr opane-1-carbonitrile* (**17**). To a solution of **15** (3.8 g, 15.3 mmol) in toluene (50 mL) at 0 ^o^C was added NaHMDS (11 mL, 21.4 mmol). The mixture was stirred for 0.5 h at 0 ^o^C. Subsequently, toluene solution containing raw material **16** (1.03 g, 15.3 mmol) was slowly added to the above reaction system. After addition, the mixture was stirred at room temperature for 2 h. The reaction was monitored by TLC. Upon completion, the mixture was quenched with cool water (150 mL) at room temperature and extracted with EtOAc (150 mL × 2). The combined organic layers were washed with water and brine, and dried over Na_2_SO_4_. The filtrate was concentrated under reduced pressure, and the residue was purified via flash column chromatography (PE/EtOAc = 5:1, v/v) to afford intermediate **17** (2.4 g, yield 56%) as a white solid. [M + H]^+^: 279.00.

Synthesis of intermediate **18**. *(R)-1-(2-chloro-6-(3-methylmorpholino)pyrimidin-4-yl)cyclopr opane-1-carboxamide* (**18**). To a solution of intermediate **17** (250.0 mg, 0.9 mmol) in MeOH (1.5 mL) and CH_2_Cl_2_ (1.5 mL) was added to KOH (200 mg, 5.0 mmol) and H_2_O_2_ (30%, 2.5 mL) at 0 ^o^C. After addition, the mixture was stirred overnight at 40 ^o^C. The reaction was monitored by TLC. Upon completion, the mixture was quenched with cool water (50 mL) at room temperature and extracted with EtOAc (50 mL × 3). The combined organic layers were washed with water and brine, and dried over Na_2_SO_4_. The filtrate was concentrated under reduced pressure, and the residue was purified via flash column chromatography (PE/EtOAc = 2:1 ~ 1:1, v/v) to afford intermediate **18** (150 mg, yield 56%) as a white solid. ^1^H NMR (500 MHz, CDCl_3_-*d*) δ 9.71 (s, 1H), 6.29 (s, 1H), 5.82 (s, 1H), 4.20 (s, 1H), 4.01 (dd, *J* = 11.5, 3.9 Hz, 1H), 3.92 – 3.74 (m, 2H), 3.68 (dd, *J* = 11.6, 3.3 Hz, 1H), 3.58 – 3.49 (m, 1H), 3.30 – 3.18 (m, 1H), 1.86 – 1.77 (m, 2H), 1.67 – 1.59 (m, 2H), 1.28 (d, *J* = 6.8 Hz, 3H).

Synthesis of intermediate **19**. *(R)-1-(2-chloro-6-(3-methylmorpholino)pyrimidin-4-yl)cyclop ropan-1-amine* (**19**). To a solution of intermediate **18** (150.0 mg, 0.5 mmol) in H_2_O (5 mL) was added to NaOH (80 mg, 2.0 mmol) and NaClO.5H_2_O (490 mg, 3.0 mmol) at room temperature. After addition, the mixture was stirred for 16 h at room temperature. The reaction was monitored by TLC. Upon completion, the mixture was quenched with cool water (50 mL) at room temperature and extracted with EtOAc (50 mL × 3). The combined organic layers were washed with water and brine, and dried over Na_2_SO_4_. The filtrate was concentrated under reduced pressure, and the residue was purified via flash column chromatography (CH_2_Cl_2_/CH_3_OH = 20:1, *v/v*) to afford intermediate **19** (100 mg, yield 74%) as a pale yellow solid. LCMS [M + H]^+^: 269.00.

Synthesis of intermediate **20**. *Tert-butyl (R)-(1-(2-chloro-6-(3-methylmorpholino)pyrimidin-4-yl)cyclopropyl)carbamate* (**20**). To a solution of intermediate **19** (1.7 g, 6.3 mmol) in CH_2_Cl_2_ (30 mL) was added to (Boc)_2_O (2.75 g, 12.6 mmol), Et_3_N (1.3 g, 12.6 mmol), and DMAP (85.0 mg, 0.63 mmol) at room temperature. After addition, the mixture was stirred 16 h at room temperature. The reaction was monitored by TLC. Upon completion, the mixture was quenched with cool water (100 mL) at room temperature and extracted with EtOAc (150 mL × 2). The combined organic layers were washed with water and brine, and dried over Na_2_SO_4_. The filtrate was concentrated under reduced pressure, and the residue was purified via flash column chromatography ((PE/EtOAc = 5:1, v/v) to afford intermediate **20** (811.8 mg, yield 35%) as a white solid.

Synthesis of intermediate **22**. *Tert-butyl (R)-(1-(6-(3-methylmorpholino)-2-(1-tosyl-1H-pyrrol o[2,3-b]pyridin-4-yl)pyrimidin-4-yl)cyclopropyl)carbamate* (**22**). To a solution of intermediate **20** (320 mg, 0.87 mmol) in 1,4-Dioxane/H_2_O (5 mL/0.5 mL) was added to **21** (415 mg, 1.04 mmol), Pd(dppf)Cl_2_ (64 mg, 0.087 mmol), and K_2_CO_3_ (240 mg, 1.7 mmol) at room temperature. The atmosphere of the reaction system was replaced by nitrogen three times. After addition, the mixture was stirred 4 h at 100 ^o^C. The reaction was monitored by TLC. Upon completion, the mixture was quenched with cool water (60 mL) at room temperature and extracted with EtOAc (60 mL × 3). The combined organic layers were washed with water and brine, and dried over Na_2_SO_4_. The filtrate was concentrated under reduced pressure, and the residue was purified via flash column chromatography ((PE/EtOAc = 1:1, v/v) to afford intermediate **22** (300 mg, yield 57%) as a yellow solid. LCMS [M + H] ^+^: 605.15.

Synthesis of intermediate **23**. *(R)-1-(6-(3-methylmorpholino)-2-(1H-pyrrolo[2,3-b]pyridin-4-yl)pyrimidin-4-yl)cyclopropan-1-amine* (**23**). To a solution of **22** (100 mg, 0.17 mmol) in 1,4-dioxane (3 mL) and THF (3 mL) was added HCl solution (4 M, 3 mL). After addition, the mixture was stirred for 1 h at room temperature. Subsequently, NaOH solution (5.9 M, 5 mL) was slowly added to the reaction system. After addition, the mixture was stirred overnight at 75 ^o^C. The reaction was monitored by TLC. Upon completion, the mixture was cooled to room temperature, quenched with cool water (30 mL), and extracted with THF (30 mL × 3). The combined organic layers were washed with water and brine, and dried over Na_2_SO_4_. The filtrate was concentrated under reduced pressure, and the residue was purified *via* C18 column chromatography (H_2_O/CH_3_CN = 1:1, *v/v*) to afford key intermediate **23** (30 mg, yield 51.9%) as a yellow solid. ^1^H NMR (500 MHz, DMSO-*d*_6_) δ 11.83 (s, 1H), 8.31 (d, *J* = 5.0 Hz, 1H), 7.66 (d, *J* = 5.0 Hz, 1H), 7.58 (t, *J* = 2.9 Hz, 1H), 7.08 (s, 1H), 6.99 (dd, *J* = 3.5, 1.6 Hz, 1H), 4.55 (s, 1H), 4.21 (d, *J* = 13.2 Hz, 1H), 3.96 (dd, *J* = 11.5, 3.7 Hz, 1H), 3.75 (d, *J* = 11.4 Hz, 1H), 3.62 (dd, *J* = 11.6, 3.3 Hz, 1H), 3.52 – 3.43 (m, 1H), 3.22 – 3.12 (m, 1H), 2.59 (s, 2H), 1.34 – 1.29 (m, 2H), 1.21 (d, *J* = 6.7 Hz, 3H), 1.04 – 0.96 (m, 2H).

Synthesis of intermediate **25**. *(R)-2-chloro-6-(3-methylmorpholino)pyrimidine-4-carbonitrile (****25****)*. To a solution of **14** (1.4 g, 14 mmol) in CH_2_Cl_2_ (25 mL) was added to **24** (2.5 g, 14.37 mmol) and Et_3_N (2.8 g, 28 mmol) at 0 ^o^C. After addition, the mixture was stirred for 2 h at room temperature. The reaction was monitored by TLC. Upon completion, the mixture was quenched with cool water (80 mL) at room temperature and extracted with CH_2_Cl_2_ (80 mL × 3). The combined organic layers were washed with water and brine, and dried over Na_2_SO_4_. The filtrate was concentrated under reduced pressure, and the residue was purified *via* flash column chromatography ((PE/EtOAc = 4:1, v/v) to afford intermediate **25** (1.5 g, yield 79.7%) as a yellow solid. ^1^H NMR (500 MHz, CDCl_3_) δ 6.74 (s, 1H), 5.58 – 2.87 (m, 7H), 1.37 (d, *J* = 6.8 Hz, 3H). LCMS [M + H]^+^: 239.1.

Synthesis of intermediate **26**. Compound **26** were synthesized according to the synthesis method of compound **22**. White solid, yield 87%. ^1^H NMR (500 MHz, CDCl_3_) δ 8.46 (d, *J* = 5.1 Hz, 1H), 8.00 (dt, *J* = 7.1, 1.8 Hz, 3H), 7.77 (d, *J* = 4.0 Hz, 1H), 7.41 (d, *J* = 4.0 Hz, 1H), 7.19 (d, *J* = 8.1 Hz, 3H), 6.72 (s, 1H), 4.35 (s, 1H), 4.03 (dd, *J* = 11.7, 4.0 Hz, 2H), 3.80 (d, *J* = 11.6 Hz, 1H), 3.68 (dd, *J* = 11.7, 3.3 Hz, 1H), 3.54 (td, *J* = 12.0, 3.1 Hz, 1H), 3.33 (td, *J* = 12.8, 4.0 Hz, 1H), 2.29 (s, 3H), 1.33 (d, *J* = 6.8 Hz, 3H). LCMS [M + H]^+^: 475.0.

Synthesis of intermediate **27**. *(R)-(6-(3-methylmorpholino)-2-(1-tosyl-1H-pyrrolo[2,3-b]pyrid in-4-yl)pyrimidin-4-yl)methanamine* (**27**). To a solution of **26** (5 g, 10.5 mmol) in NH_3_·MeOH (100 mL) was added to Raney Nickel (500 mg) at room temperature. After addition, the atmosphere of the reaction system was replaced by hydrogen three times. The mixture was stirred for 16 h at rt. The reaction was monitored by TLC. Upon completion, static reaction system, filtration. The filtrate was concentrated under reduced pressure, and the residue was purified via C18 column chromatography (H_2_O/CH_3_CN = 2:3, v/v) to afford intermediate **27** (2.0 g, yield 39.6%) as a white solid. LCMS [M + H]^+^: 479.32.

Synthesis of intermediate **28**. *(R)-(6-(3-methylmorpholino)-2-(1H-pyrrolo[2,3-b]pyridin-4-yl) pyrimidin-4-yl)methanamine (****28****)*. To a solution of **27** (300 mg, 0.63 mmol) in 1,4-dioxane/THF (4 mL / 4 mL) was added NaOH solution (20%, 1.6 mL) at room temperature. After addition, the mixture was stirred overnight at 75 ^o^C. The reaction was monitored by TLC. Upon completion, the reaction solvent was concentrated under reduced pressure, and the residue was purified via C18 column chromatography (H_2_O/CH_3_CN = 3:2, v/v) to afford key intermediate **28** (150 mg, yield 73.4%) as a yellow solid. ^1^H NMR (500 MHz, DMSO-*d*_6_) δ 11.78 (s, 1H), 8.31 (d, *J* = 5.0 Hz, 1H), 7.98 (d, *J* = 5.0 Hz, 1H), 7.56(d, *J* = 3.4 Hz, 1H), 7.25 (d, *J* = 3.4 Hz, 1H), 6.86 (s, 1H), 4.56 (s, 1H), 4.16 (d, *J* = 12.8 Hz, 1H), 4.00(dd, *J* = 11.4, 3.7 Hz, 1H), 3.83 – 3.74 (m, 3H), 3.66 (dd, *J* = 11.5, 3.2 Hz, 1H), 3.54 – 3.48 (m, 1H), 3.29– 3.21 (m, 1H), 1.26 (d, *J* = 6.7 Hz, 3H).

General procedure for the synthesis of intermediates **31a-31d**. To a solution of corresponding amines **30** (0.59 mmol) in DMF (7.0 mL) were added **29** (0.54 mmol), DIPEA (1.62 mmol), HOBT (0.72 mmol), and EDCI (1.43 mmol). The resulting solution was stirred at room temperature for 6 h. The reaction was monitored by TLC. Upon completion, the mixture was quenched with cool water (100 mL) at room temperature and extracted with EtOAc (100 mL × 3). The combined organic layers were washed with water and brine, and dried over Na_2_SO_4_. The drying agent was filtered off. The filtrate was concentrated under reduced pressure, and the residue was purified via flash column chromatography (CH_2_Cl_2_/MeOH 49:1, v/v) to give the corresponding intermediates **31a-31d** as a white solid. Yield 73% ~ 89%.

*Methyl (2-fluoro-4-((4-oxo-3,4-dihydrophthalazin-1-yl)methyl)benzoyl)glycinate (****31a****)* ^1^H NMR (500 MHz, DMSO-*d*_6_) δ 12.60 (s, 1H), 8.63 (td, *J* = 5.7, 3.2 Hz, 1H), 8.27 (dd, *J* = 7.9, 1.4 Hz, 1H), 8.02 – 7.94 (m, 1H), 7.90 (ddd, *J* = 8.1, 7.2, 1.4 Hz, 1H), 7.83 (td, *J* = 7.5, 1.2 Hz, 1H), 7.63 (dd, *J* = 7.0, 2.4 Hz, 1H), 7.52 (ddd, *J* = 8.5, 4.8, 2.4 Hz, 1H), 7.25 (dd, *J* = 10.6, 8.5 Hz, 1H), 4.35 (s, 2H), 4.00 (d, *J* = 5.8 Hz, 2H), 3.65 (s, 3H).

*Methyl 3-(2-fluoro-4-((4-oxo-3,4-dihydrophthalazin-1-yl)methyl)benzamido)propanoate (****31b****)* ^1^H NMR (500 MHz, DMSO-*d*_6_) δ 12.60 (s, 1H), 8.33 (td, *J* = 5.6, 2.7 Hz, 1H), 8.26 (dd, *J* = 7.8, 1.4 Hz, 1H), 7.97 (dt, *J* = 8.0, 0.9 Hz, 1H), 7.89 (ddd, *J* = 8.1, 7.2, 1.5 Hz, 1H), 7.83 (ddd, *J* = 8.4, 7.3, 1.2 Hz, 1H), 7.56 (dd, *J* = 6.9, 2.4 Hz, 1H), 7.47 (ddd, *J* = 8.5, 4.9, 2.4 Hz, 1H), 7.21 (dd, *J* = 10.4, 8.5 Hz, 1H), 4.33 (s, 2H), 3.59 (s, 3H), 3.46 (td, *J* = 6.8, 5.5 Hz, 2H), 2.56 (t, *J* = 6.9 Hz, 2H).

*Methyl 4-(2-fluoro-4-((4-oxo-3,4-dihydrophthalazin-1-yl)methyl)benzamido)butanoate (****31c****)* ^1^H NMR (500 MHz, DMSO-*d*_6_) δ 12.60 (s, 1H), 8.31 (dt, *J* = 5.8, 3.7 Hz, 1H), 8.27 (dd, *J* = 7.8, 1.4 Hz, 1H), 7.98 (d, *J* = 7.9 Hz, 1H), 7.94 – 7.79 (m, 2H), 7.56 (dd, *J* = 6.9, 2.4 Hz, 1H), 7.45 (ddd, *J* = 8.5, 4.9, 2.4 Hz, 1H), 7.21 (dd, *J* = 10.3, 8.4 Hz, 1H), 4.33 (s, 2H), 3.58 (s, 3H), 3.25 (q, *J* = 6.6 Hz, 2H), 2.36 (t, *J* = 7.4 Hz, 2H), 1.75 (p, *J* = 7.1 Hz, 2H).

*Methyl 1-(2-fluoro-4-((4-oxo-3,4-dihydrophthalazin-1-yl)methyl)benzoyl)piperidine-4-carbox ylate (****31d****)* ^1^H NMR (500 MHz, DMSO-*d*_6_) δ 12.59 (s, 1H), 8.27 (dd, *J* = 7.7, 1.6 Hz, 1H), 7.98 (dt, *J* = 8.3, 0.9 Hz, 1H), 7.86 (dtd, *J* = 27.6, 7.3, 1.3 Hz, 2H), 7.41 (ddd, *J* = 8.1, 5.0, 2.2 Hz, 1H), 7.35 (s, 1H), 7.22 (t, *J* = 9.0 Hz, 1H), 4.35 (d, *J* = 13.4 Hz, 3H), 3.62 (s, 3H), 3.31 (d, *J* = 13.4 Hz, 1H), 3.06 (t, *J* = 12.4 Hz, 1H), 3.00 – 2.87 (m, 1H), 2.65 (tt, *J* = 11.0, 3.8 Hz, 1H), 1.92 (dd, *J* = 13.2, 3.9 Hz, 1H), 1.74 (s, 1H), 1.59 – 1.29 (m, 2H).

Synthesis of intermediate **33**. *Tert-butyl (R)-4-(N-((6-(3-methylmorpholino)-2-(1H-pyrrolo[2,3-b]pyridin-4-yl)pyrimidin-4-yl)methyl)sulfamoyl)piperidine-1-carboxylate (****33****)*. To a solution of **28** (150 mg, 0.46 mmol) in DMF (3.0 mL) were added **32** (156.1 mg, 0.55 mmol) and Et_3_N (178.0 mg, 1.38 mmol). The resulting solution was stirred at room temperature for 3 h. The reaction was monitored by TLC. Upon completion, the mixture was quenched with cool water (50 mL) at room temperature and extracted with EtOAc (50 mL × 3). The combined organic layers were washed with water and brine, and dried over Na_2_SO_4_. The drying agent was filtered off. The filtrate was concentrated under reduced pressure, and the residue was purified *via* flash column chromatography (CH_2_Cl_2_/MeOH 97:3, *v/v*) to give intermediate **33** (194 mg, yield 74%) as a white solid. ^1^H NMR (500 MHz, DMSO-*d*_6_) δ 11.76 (s, 1H), 8.33 (d, *J* = 5.0 Hz, 1H), 7.98 (d, *J* = 5.0 Hz, 1H), 7.82 (s, 1H), 7.58 (dd, *J* = 3.4, 1.8 Hz, 1H), 7.27 (dd, *J* = 3.4, 1.3 Hz, 1H), 6.81 (s, 1H), 5.75 (s, 2H), 4.52 (s, 1H), 4.25 (s, 2H), 4.16 (d, *J* = 12.9 Hz, 1H), 4.07 – 4.00 (m, 1H), 3.95 (d, *J* = 12.5 Hz, 2H), 3.82 (d, *J* = 11.4 Hz, 1H), 3.68 (dd, *J* = 11.6, 3.2 Hz, 1H), 3.53 (td, *J* = 11.8, 2.9 Hz, 2H), 3.35 – 3.23 (m, 2H), 1.99 (d, *J* = 12.6 Hz, 2H), 1.47 – 1.39 (m, 2H), 1.37 (s, 9H), 1.28 (d, *J* = 6.7 Hz, 3H).

General procedure for the synthesis of intermediates **36a-36c**. To a solution of corresponding acids **35** (1.2 mmol) in DMF (7.0 mL) were added **34** (1.0 mmol), DIPEA (3.0 mmol), HOBT (1.3 mmol), and EDCI (2.60 mmol). The resulting solution was stirred at room temperature for 6 h. The reaction was monitored by TLC. Upon completion, the mixture was quenched with cool water (100 mL) at room temperature and extracted with EtOAc (100 mL × 2). The combined organic layers were washed with water and brine, and dried over Na_2_SO_4_. The drying agent was filtered off. The filtrate was concentrated under reduced pressure, and the residue was purified via flash column chromatography (CH_2_Cl_2_/MeOH 97:3, *v/v*) to give the corresponding intermediates **36a-36c** as a white solid. Yield 71% ~ 84%.

*Methyl 4-(4-(2-fluoro-5-((4-oxo-3,4-dihydrophthalazin-1-yl)methyl)benzoyl)piperazine-1-car bonyl)benzoate (****36a****)* ^1^H NMR (500 MHz, DMSO-*d*_6_) δ 12.60 (s, 1H), 8.26 (s, 1H), 8.07 – 7.76 (m, 5H), 7.56 (s, 2H), 7.50 – 7.30 (m, 2H), 7.24 (s, 1H), 4.43 – 4.23 (m, 2H), 3.88 (d, *J* = 4.0 Hz, 3H), 3.67 (d, *J* = 32.8 Hz, 4H), 3.25 (d, *J* = 56.9 Hz, 4H).

*Methyl 5-(4-(2-fluoro-5-((4-oxo-3,4-dihydrophthalazin-1-yl)methyl)benzoyl)piperazine-1-car bonyl)picolinate (****36b****)* ^1^H NMR (500 MHz, DMSO-*d*_6_) δ 12.59 (d, *J* = 19.2 Hz, 1H), 9.08 (dd, *J* = 20.3, 2.1 Hz, 1H), 8.42 (ddd, *J* = 9.9, 8.1, 2.2 Hz, 1H), 8.26 (dd, *J* = 16.8, 7.8 Hz, 1H), 8.05 – 7.71 (m, 4H), 7.53 – 7.33 (m, 2H), 7.24 (dt, *J* = 23.9, 9.0 Hz, 1H), 4.33 (d, *J* = 19.4 Hz, 2H), 3.92 (d, *J* = 3.7 Hz, 3H), 3.75 (s, 2H), 3.69 – 3.56 (m, 2H), 3.45 (t, *J* = 5.3 Hz, 1H), 3.31 (dd, *J* = 10.6, 5.8 Hz, 2H), 3.19 (t, *J* = 5.1 Hz, 1H).

*Methyl 6-(4-(2-fluoro-5-((4-oxo-3,4-dihydrophthalazin-1-yl)methyl)benzoyl)piperazine-1-car bonyl)nicotinate (****36c****)* ^1^H NMR (500 MHz, CDCl_3_) δ 10.92 (s, 1H), 8.71 (s, 1H), 8.40 (d, *J* = 6.1 Hz, 1H), 8.14 (s, 1H), 7.98 – 7.79 (m, 1H), 7.79 – 7.58 (m, 3H), 7.28 (d, *J* = 5.4 Hz, 2H), 6.99 (s, 1H), 4.23 (s, 2H), 3.97 (s, 3H), 3.76 (d, *J* = 60.8 Hz, 4H), 3.56 – 3.07 (m, 4H).

Compounds **38a** were synthesized according to the procedure described for compound **33**. White solid, yield 53%%. *Methyl (R)-4-(N-(1-(6-(3-methylmorpholino)-2-(1H-pyrrolo[2,3-b]pyridin-4-yl)pyrimidin-4-yl)cyclopropyl)sulfamoyl)benzoate (****38a****)* ^1^H NMR (500 MHz, DMSO-*d*_6_) δ 11.80 (s, 1H), 8.80 (s, 1H), 8.29 (d, *J* = 5.0 Hz, 1H), 7.79 – 7.68 (m, 4H), 7.58 (dd, *J* = 3.4, 2.6 Hz, 1H), 7.48 (d, *J* = 5.0 Hz, 1H), 6.86 (dd, *J* = 3.4, 1.9 Hz, 1H), 6.79 (s, 1H), 4.42 – 4.06 (m, 2H), 3.94 (dd, *J* = 11.3, 3.7 Hz, 1H), 3.80 (s, 3H), 3.71 (d, *J* = 11.4 Hz, 1H), 3.56 (dd, *J* = 11.5, 3.2 Hz, 1H), 3.42 (td, *J* = 11.8, 3.0 Hz, 1H), 3.10 – 2.87 (m, 1H), 1.61 (dq, *J* = 8.3, 3.2, 2.5 Hz, 1H), 1.54 – 1.39 (m, 2H), 1.39 – 1.29 (m, 1H), 1.12 (d, *J* = 6.7 Hz, 3H).

Synthesis of intermediate **40**. To a solution of commercially available starting material **34** (366.40 mg, 1.0 mmol) in DMF (7.0 mL) was added to material **39** (221.54 mg, 1.2 mmol) and DIPEA (193.5 mg, 1.5 mmol) at rt. After addition, the mixture was stirred 5 h at 60 ^o^C. The reaction was monitored by TLC. Upon completion, the mixture was quenched with cool water (50 mL) at room temperature and extracted with EtOAc (50 mL × 3). The combined organic layers were washed with water and brine, and dried over Na_2_SO_4_. The filtrate was concentrated under reduced pressure, and the residue was purified via flash column chromatography (PE/EtOAc = 2:1 ~ 1:1, v/v) to afford intermediate **40** (380 mg, yield 74%) as a white solid. *Methyl 4-((4-(2-fluoro-5-((4-oxo-3,4-dihydrophthalazin-1-yl)methyl)benzoyl)piperazin-1-yl)methyl)benzoate* *(****40****)* ^1^H NMR (500 MHz, CDCl_3_) δ 10.82 (s, 1H), 8.45 – 8.35 (m, 1H), 8.00 – 7.89 (m, 2H), 7.77 – 7.61 (m, 3H), 7.32 (d, *J* = 8.1 Hz, 2H), 7.27 – 7.20 (m, 2H), 6.94 (t, *J* = 8.8 Hz, 1H), 4.21 (s, 2H), 3.84 (s, 3H), 3.73 (s, 2H), 3.50 (s, 2H), 3.23 (s, 2H), 2.45 (t, *J* = 5.1 Hz, 2H), 2.28 (s, 2H).

Synthesis of intermediate **42a-42b**. Compounds **42a-42b** were synthesized according to the synthesis method of compounds **36a-36c**. White solid, yield 81% ~ 88%.

*Methyl 2-(4-(2-fluoro-5-((4-oxo-3,4-dihydrophthalazin-1-yl)methyl)benzoyl)piperazin-1-yl)py rimidine-5-carboxylate (****42a****)* ^1^H NMR (500 MHz, DMSO-*d*_6_) δ 12.60 (s, 1H), 8.82 (s, 2H), 8.26 (dd, *J* = 7.8, 1.4 Hz, 1H), 7.98 (d, *J* = 7.9 Hz, 1H), 7.90 (ddd, *J* = 8.1, 7.1, 1.5 Hz, 1H), 7.84 (td, *J* = 7.5, 1.2 Hz, 1H), 7.45 (ddd, *J* = 8.1, 5.1, 2.3 Hz, 1H), 7.40 (dd, *J* = 6.5, 2.4 Hz, 1H), 7.25 (dd, *J* = 9.4, 8.5 Hz, 1H), 4.34 (s, 2H), 3.95 (t, *J* = 5.1 Hz, 2H), 3.81 (s, 3H), 3.80 – 3.77 (m, 2H), 3.73 (d, *J* = 5.8 Hz, 2H), 3.32 – 3.28 (m, 2H).

*Methyl 4-(4-(2-fluoro-5-((4-oxo-3,4-dihydrophthalazin-1-yl)methyl)benzoyl)piperazin-1-yl)be nzoate (****42b****)* ^1^H NMR (500 MHz, DMSO-*d*_6_) δ 12.59 (s, 1H), 8.27 (dd, *J* = 7.9, 1.4 Hz, 1H), 8.01 – 7.94 (m, 1H), 7.90 (ddd, *J* = 8.1, 7.2, 1.5 Hz, 1H), 7.87 – 7.77 (m, 3H), 7.50 – 7.43 (m, 1H), 7.39 (dd, *J* = 6.5, 2.3 Hz, 1H), 7.32 – 7.19 (m, 1H), 7.05 – 6.92 (m, 2H), 4.34 (s, 2H), 3.78 (s, 3H), 3.75 (s, 2H), 3.42 (t, *J* = 5.4 Hz, 2H), 3.32 – 3.28 (m, 2H), 3.25 (dd, *J* = 6.4, 3.6 Hz, 2H).

**Part 3: ^1^H NMR and ^13^C NMR spectra for compounds A1-A12 and B1-B14**

*(R)-2-fluoro-N-(1-(6-(3-methylmorpholino)-2-(1H-pyrrolo[2,3-b]pyridin-4-yl)pyrimidin-4-yl)cyc lopropyl)-5-((4-oxo-3,4-dihydrophthalazin-1-yl)methyl)benzamide (****A1****)*

*(R)-2-fluoro-N-(2-((1-(6-(3-methylmorpholino)-2-(1H-pyrrolo[2,3-b]pyridin-4-yl)pyrimidin-4-yl)c yclopropyl)amino)-2-oxoethyl)-5-((4-oxo-3,4-dihydrophthalazin-1-yl)methyl)benzamide (****A2****)*

*(R)-2-fluoro-N-(3-((1-(6-(3-methylmorpholino)-2-(1H-pyrrolo[2,3-b]pyridin-4-yl)pyrimidin-4-yl)c yclopropyl)amino)-3-oxopropyl)-5-((4-oxo-3,4-dihydrophthalazin-1-yl)methyl)benzamide (****A3****)*

*(R)-2-fluoro-N-(4-((1-(6-(3-methylmorpholino)-2-(1H-pyrrolo[2,3-b]pyridin-4-yl)pyrimidin-4-yl)c yclopropyl)amino)-4-oxobutyl)-5-((4-oxo-3,4-dihydrophthalazin-1-yl)methyl)benzamide (****A4****)*

*(R)-1-(2-fluoro-5-((4-oxo-3,4-dihydrophthalazin-1-yl)methyl)benzoyl)-N-(1-(6-(3-methylmorpholi no)-2-(1H-pyrrolo[2,3-b]pyridin-4-yl)pyrimidin-4-yl)cyclopropyll)piperidine-4-carboxamide (****A5****)*

*(R)-4-(2-fluoro-5-((4-oxo-3,4-dihydrophthalazin-1-yl)methyl)benzoyl)-N-(1-(6-(3-methylmorpholi no)-2-(1H-pyrrolo[2,3-b]pyridin-4-yl)pyrimidin-4-yl)cyclopropyll)piperazine-1-carboxamide (****A6****)*

*(R)-4-(4-(2-fluoro-5-((4-oxo-3,4-dihydrophthalazin-1-yl)methyl)benzoyl)piperazine-1-carbonyl)-N-(1-(6-(3-methylmorpholino)-2-(1H-pyrrolo[2,3-b]pyridin-4-yl)pyrimidin-4-yl)cyclopropyl)ben zamide* *(****A7****)*

*(R)-4-(4-(2-fluoro-5-((4-oxo-3,4-dihydrophthalazin-1-yl)methyl)benzoyl)piperazine-1-carbonyl)-N-(1-(6-(3-methylmorpholino)-2-(1H-pyrrolo[2,3-b]pyridin-4-yl)pyrimidin-4-yl)cyclopropyl)benz enesulfonamide (****A8****)*

*(R)-6-(4-(2-fluoro-5-((4-oxo-3,4-dihydrophthalazin-1-yl)methyl)benzoyl)piperazine-1-carbonyl)-N-(1-(6-(3-methylmorpholino)-2-(1H-pyrrolo[2,3-b]pyridin-4-yl)pyrimidin-4-yl)cyclopropyl)nicot inamide (****A9****)*

*(R)-4-((4-(2-fluoro-5-((4-oxo-3,4-dihydrophthalazin-1-yl)methyl)benzoyl)piperazin-1-yl)methyl)-N-(1-(6-(3-methylmorpholino)-2-(1H-pyrrolo[2,3-b]pyridin-4-yl)pyrimidin-4-yl)cyclopropyl)benz amide (****A10****)*

*(R)-4-(4-(2-fluoro-5-((4-oxo-3,4-dihydrophthalazin-1-yl)methyl)benzoyl)piperazin-1-yl)-N-(1-(6-(3-methylmorpholino)-2-(1H-pyrrolo[2,3-b]pyridin-4-yl)pyrimidin-4-yl)cyclopropyl)benzamide (****A11****)*

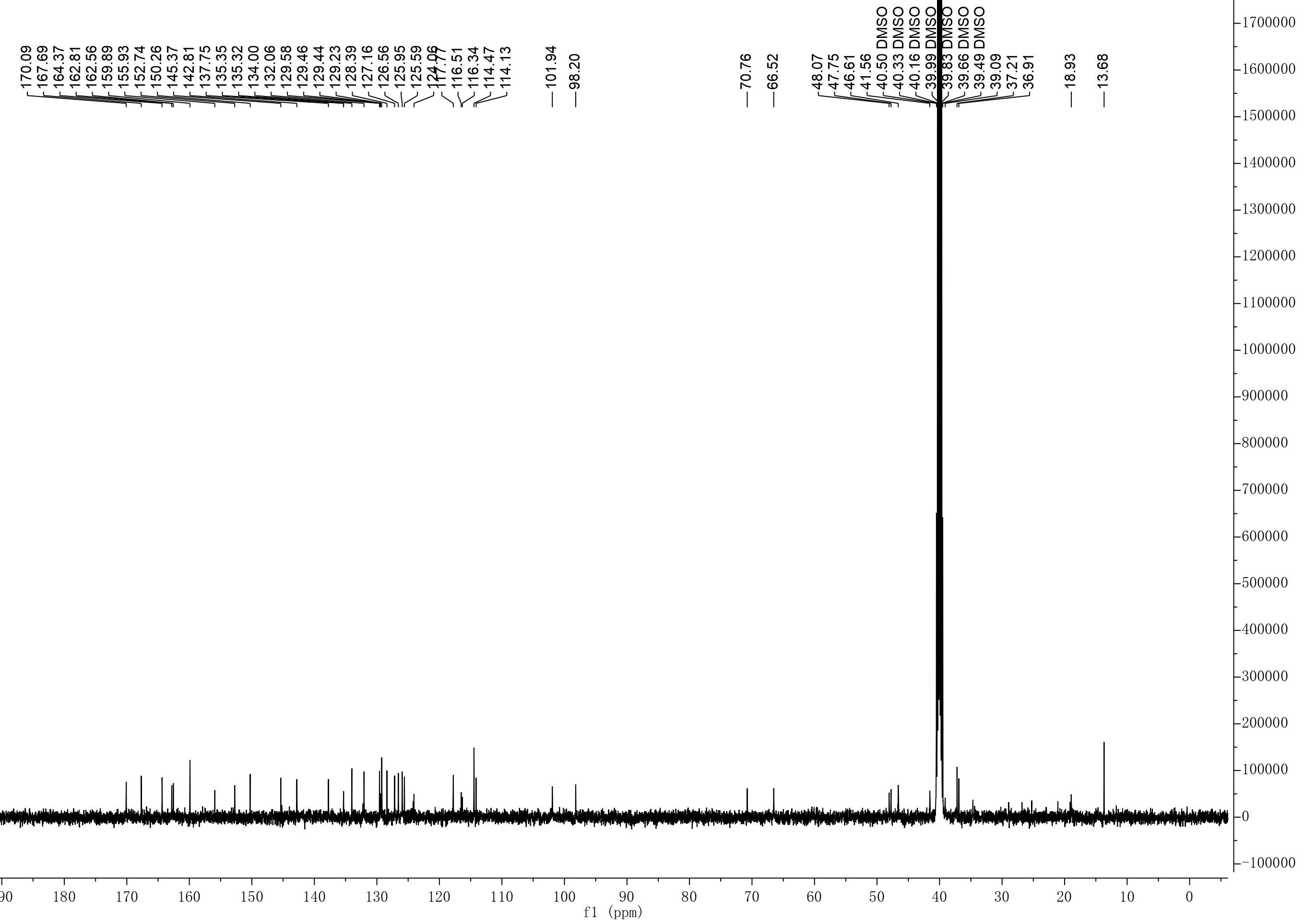


*(R)-2-(4-(2-fluoro-5-((4-oxo-3,4-dihydrophthalazin-1-yl)methyl)benzoyl)piperazin-1-yl)-N-(1-(6-(3-methylmorpholino)-2-(1H-pyrrolo[2,3-b]pyridin-4-yl)pyrimidin-4-yl)cyclopropyl)pyrimidine-5-carboxamide (****A12****)*

*(R)-2-fluoro-N-((6-(3-methylmorpholino)-2-(1H-pyrrolo[2,3-b]pyridin-4-yl)pyrimidin-4-yl)methyl) -5-((4-oxo-3,4-dihydrophthalazin-1-yl)methyl)benzamide (****B1****)*

*(R)-2-fluoro-N-(2-(((6-(3-methylmorpholino)-2-(1H-pyrrolo[2,3-b]pyridin-4-yl)pyrimidin-4-yl)me thyl)amino)-2-oxoethyl)-5-((4-oxo-3,4-dihydrophthalazin-1-yl)methyl)benzamide (****B2****)*

*(R)-2-fluoro-N-(3-(((6-(3-methylmorpholino)-2-(1H-pyrrolo[2,3-b]pyridin-4-yl)pyrimidin-4-yl)me thyl)amino)-3-oxopropyl)-5-((4-oxo-3,4-dihydrophthalazin-1-yl)methyl)benzamide (****B3****)*

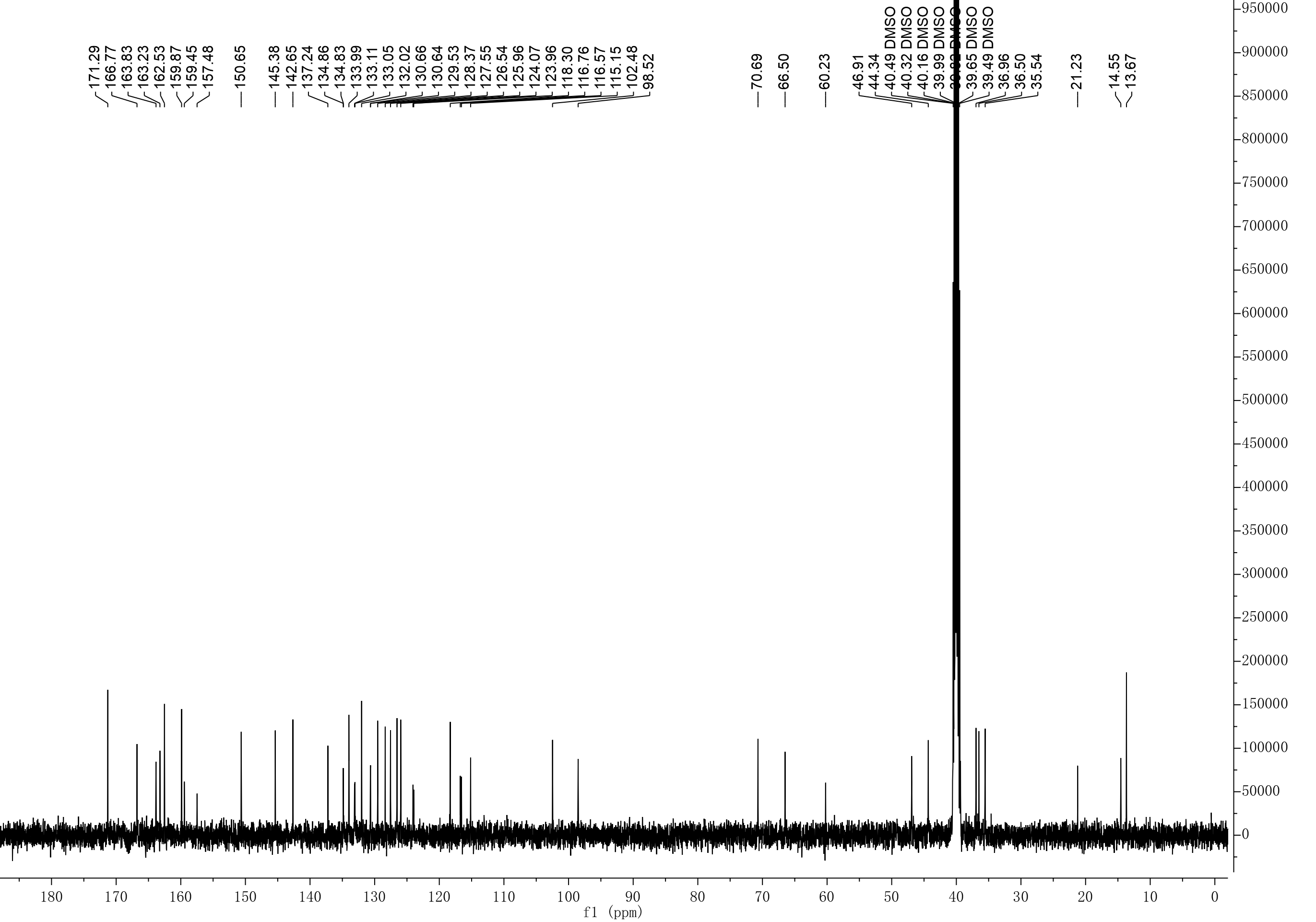


*(R)-2-fluoro-N-(4-(((6-(3-methylmorpholino)-2-(1H-pyrrolo[2,3-b]pyridin-4-yl)pyrimidin-4-yl)me thyl)amino)-4-oxobutyl)-5-((4-oxo-3,4-dihydrophthalazin-1-yl)methyl)benzamide (****B4****)*

*(R)-1-(2-fluoro-5-((4-oxo-3,4-dihydrophthalazin-1-yl)methyl)benzoyl)-N-((6-(3-methylmorpholin o)-2-(1H-pyrrolo[2,3-b]pyridin-4-yl)pyrimidin-4-yl)methyl)piperidine-4-carboxamide (****B5****)*

*(R)-1-(2-fluoro-5-((4-oxo-3,4-dihydrophthalazin-1-yl)methyl)benzoyl)-N-((6-(3-methylmorpholino) -2-(1H-pyrrolo[2,3-b]pyridin-4-yl)pyrimidin-4-yl)methyl)piperidine-4-sulfonamide (****B6****)*

*(R)-4-(2-fluoro-5-((4-oxo-3,4-dihydrophthalazin-1-yl)methyl)benzoyl)-N-((6-(3-methylmorpholin o)-2-(1H-pyrrolo[2,3-b]pyridin-4-yl)pyrimidin-4-yl)methyl)piperazine-1-carboxamide (****B7****)*

*(R)-4-(4-(2-fluoro-5-((4-oxo-3,4-dihydrophthalazin-1-yl)methyl)benzoyl)piperazine-1-carbonyl)-N-((6-(3-methylmorpholino)-2-(1H-pyrrolo[2,3-b]pyridin-4-yl)pyrimidin-4-yl)methyl)benzamide (****B8****)*

*(R)-4-(4-(2-fluoro-5-((4-oxo-3,4-dihydrophthalazin-1-yl)methyl)benzoyl)piperazine-1-carbonyl)-N-((6-(3-methylmorpholino)-2-(1H-pyrrolo[2,3-b]pyridin-4-yl)pyrimidin-4-yl)methyl)benzenesu lfonamide (****B9****)*

*(R)-6-(4-(2-fluoro-5-((4-oxo-3,4-dihydrophthalazin-1-yl)methyl)benzoyl)piperazine-1-carbonyl)-N-((6-(3-methylmorpholino)-2-(1H-pyrrolo[2,3-b]pyridin-4-yl)pyrimidin-4-yl)methyl)nicotinami de (****B10****)*

*(R)-5-(4-(2-fluoro-5-((4-oxo-3,4-dihydrophthalazin-1-yl)methyl)benzoyl)piperazine-1-carbonyl)-N -((6-(3-methylmorpholino)-2-(1H-pyrrolo[2,3-b]pyridin-4-yl)pyrimidin-4-yl)methyl)picolinamide (****B11****)*

*(R)-4-((4-(2-fluoro-5-((4-oxo-3,4-dihydrophthalazin-1-yl)methyl)benzoyl)piperazin-1-yl)methyl)-N -((6-(3-methylmorpholino)-2-(1H-pyrrolo[2,3-b]pyridin-4-yl)pyrimidin-4-yl)methyl)benzamide (****B12****)*

*(R)-4-(4-(2-fluoro-5-((4-oxo-3,4-dihydrophthalazin-1-yl)methyl)benzoyl)piperazin-1-yl)-N-((6-(3-methylmorpholino)-2-(1H-pyrrolo[2,3-b]pyridin-4-yl)pyrimidin-4-yl)methyl)benzamide (****B13****)*

*(R)-2-(4-(2-fluoro-5-((4-oxo-3,4-dihydrophthalazin-1-yl)methyl)benzoyl)piperazin-1-yl)-N-((6-(3-methylmorpholino)-2-(1H-pyrrolo[2,3-b]pyridin-4-yl)pyrimidin-4-yl)methyl)pyrimidine-5-carboxa mide (****B14****)*

**Part 4: HPLC spectra for compounds A1-A12 and B1-B14**

Analysis Report

Sample Name: **A1**

Column: Phenomenex Luna^®^ 5 μm C18(2) 100 Å 250 X 4.6 mm

Mobile phase: A: H_2_O; B: MeOH

Date: 2024-08-27

Injection volume: 15 uL

Flow rate: 0.5 mL/min

Method: 70% B for 0.5 min, 70% B to 100% B in 6 min gradient, 100% B for 9 min. stop at 16.01 min


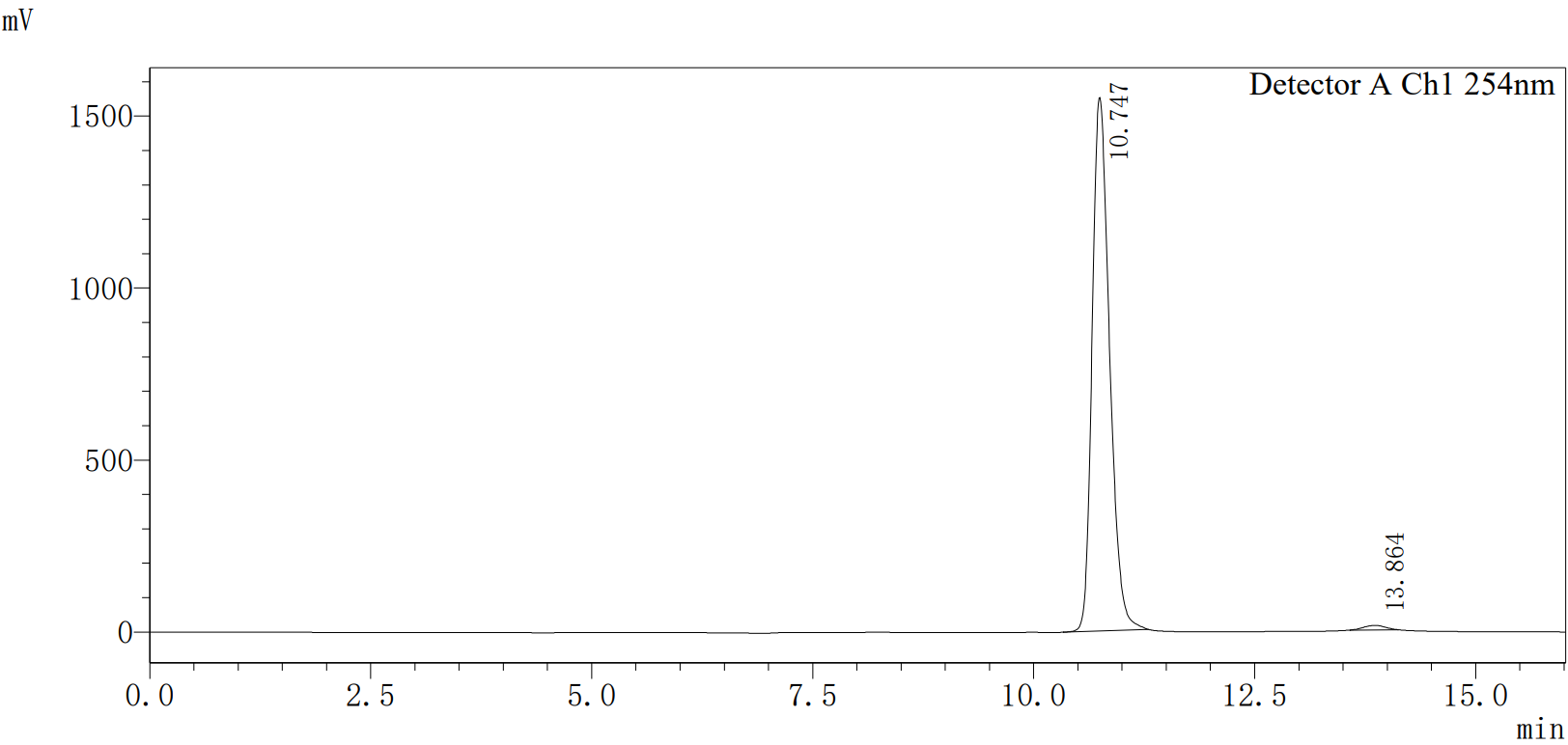


<Peak table>

Detector A Ch1 254nm

| Number | Retention time | Peak area | Peak area % |
| --- | --- | --- | --- |
| 1 | 10.747 | 21260128 | 98.983 |
| 2 | 13.864 | 218470 | 1.017 |
| Total |  | 21478598 | 100.000 |

Sample Name: **A2**

Column: Phenomenex Luna^®^ 5 μm C18(2) 100 Å 250 X 4.6 mm

Mobile phase: A: H_2_O; B: MeOH

Date: 2024-08-27

Injection volume: 15 uL

Flow rate: 0.5 mL/min

Method: 70% B for 0.5 min, 70% B to 100% B in 6 min gradient, 100% B for 9 min. stop at 16.01 min

*
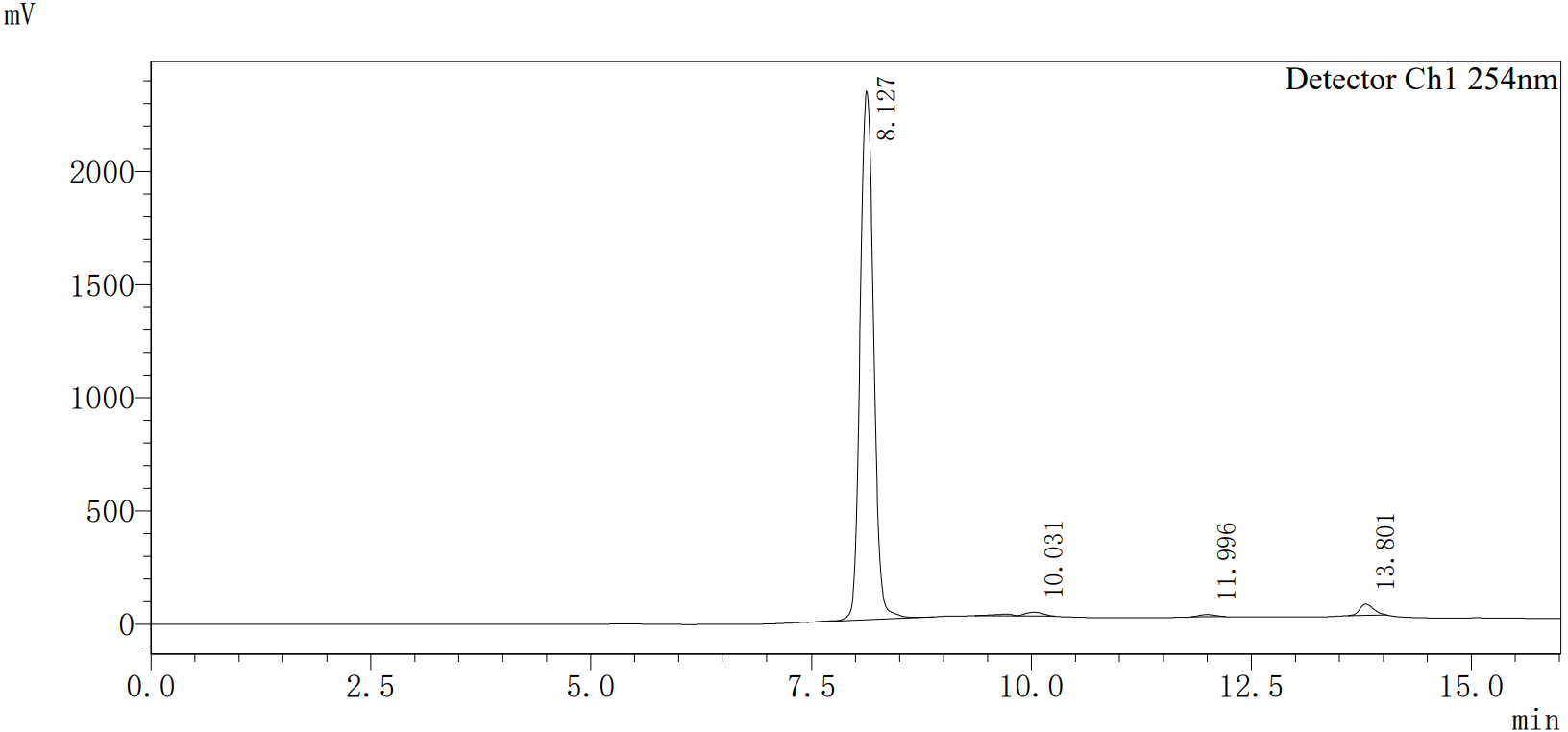
*

<Peak table>

Detector A Ch1 254nm

| Number | Retention time | Peak area | Peak area % |
| --- | --- | --- | --- |
| 1 | 8.127 | 24858502 | 96.093 |
| 2 | 10.031 | 348721 | 1.348 |
| 3 | 11.996 | 122122 | 0.472 |
| 4 | 13.8001 | 539762 | 2.087 |
| Total |  | 25869108 | 100.000 |

Sample Name: **A3**

Column: Phenomenex Luna^®^ 5 μm C18(2) 100 Å 250 X 4.6 mm

Mobile phase: A: H_2_O; B: MeOH

Date: 2024-08-27

Injection volume: 15 uL

Flow rate: 0.5 mL/min

Method: 70% B for 0.5 min, 70% B to 100% B in 6 min gradient, 100% B for 9 min. stop at 16.01 min


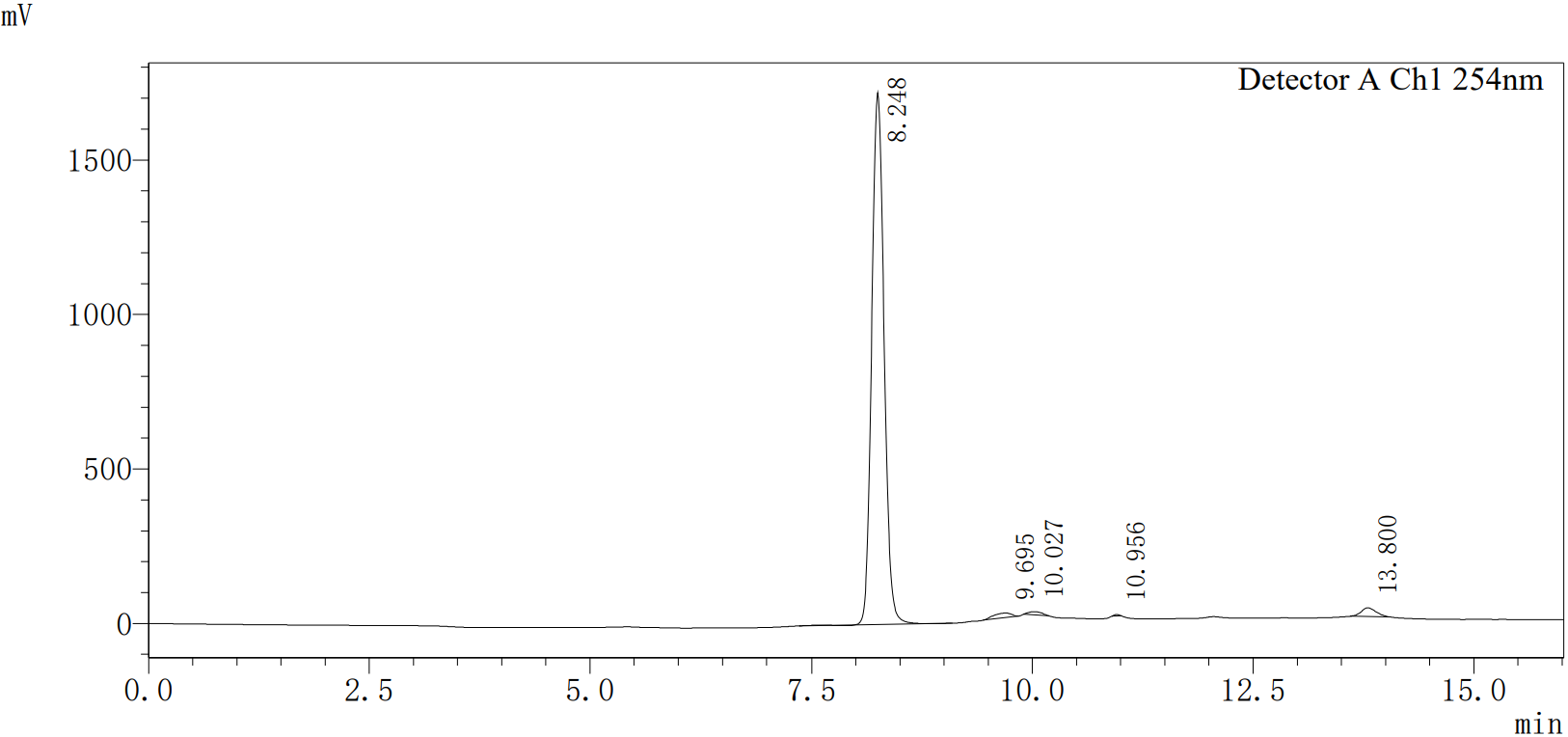


<Peak table>

Detector A Ch1 254nm

| Number | Retention time | Peak area | Peak area % |
| --- | --- | --- | --- |
| 1 | 8.248 | 16813258 | 96.182 |
| 2 | 9.695 | 220934 | 1.264 |
| 3 | 10.027 | 107031 | 0.612 |
| 4 | 10.956 | 16211 | 0.093 |
| 5 | 13.800 | 323267 | 1.849 |
| Total |  | 25869108 | 100.000 |

Sample Name: **A4**

Column: Phenomenex Luna^®^ 5 μm C18(2) 100 Å 250 X 4.6 mm

Mobile phase: A: H_2_O; B: MeOH

Date: 2024-08-27

Injection volume: 15 uL

Flow rate: 0.5 mL/min

Method: 70% B for 0.5 min, 70% B to 100% B in 6 min gradient, 100% B for 9 min. stop at 16.01 min

*
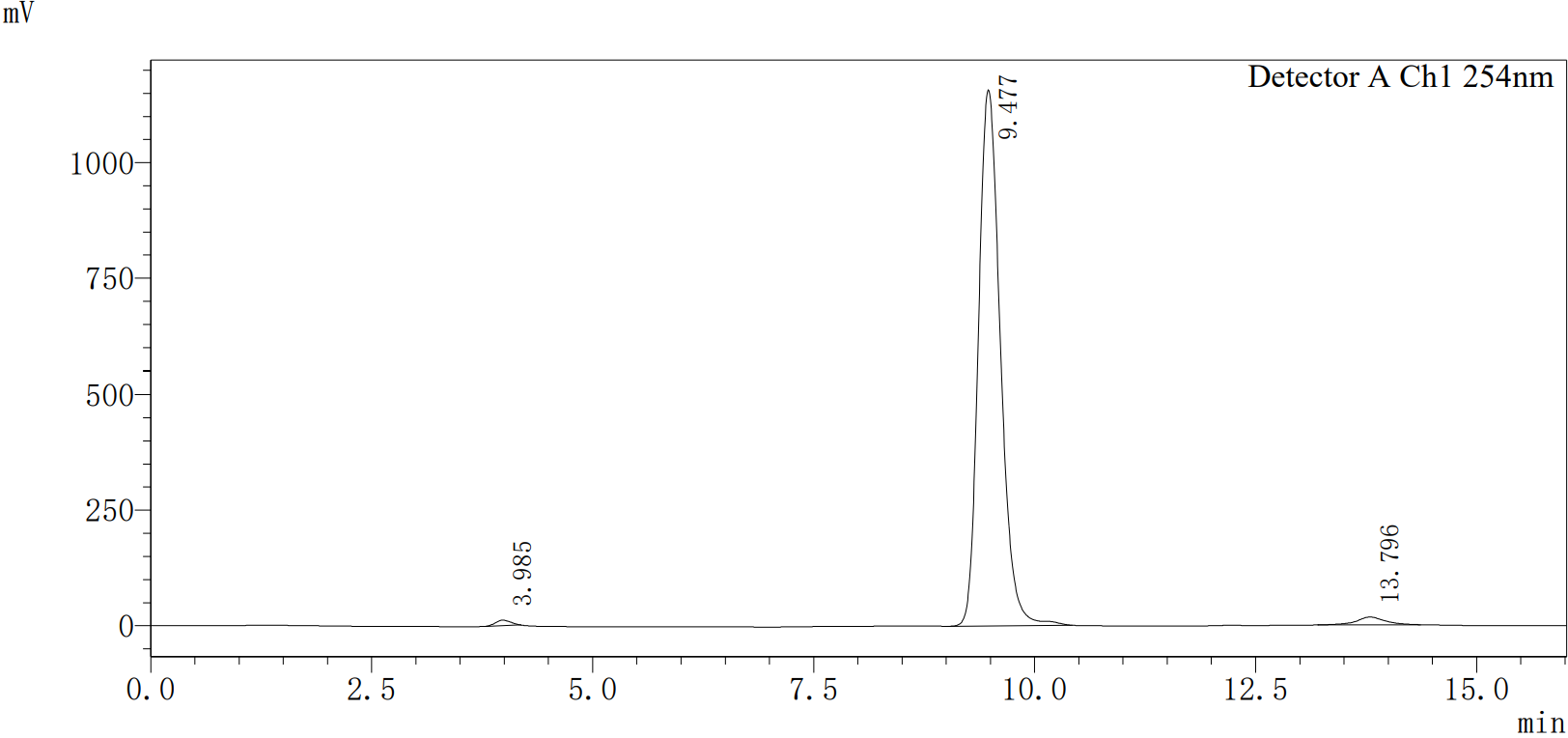
*

<Peak table>

Detector A Ch1 254nm

| Number | Retention time | Peak area | Peak area % |
| --- | --- | --- | --- |
| 1 | 3.985 | 142203 | 0.695 |
| 2 | 9.477 | 19923615 | 97.410 |
| 3 | 13.796 | 387484 | 1.894 |
| Total |  | 20453303 | 100.000 |

Sample Name: **A5**

Column: Phenomenex Luna^®^ 5 μm C18(2) 100 Å 250 X 4.6 mm

Mobile phase: A: H_2_O; B: MeOH

Date: 2024-08-27

Injection volume: 15 uL

Flow rate: 0.5 mL/min

Method: 70% B for 0.5 min, 70% B to 100% B in 6 min gradient, 100% B for 9 min. stop at 16.01 min

*
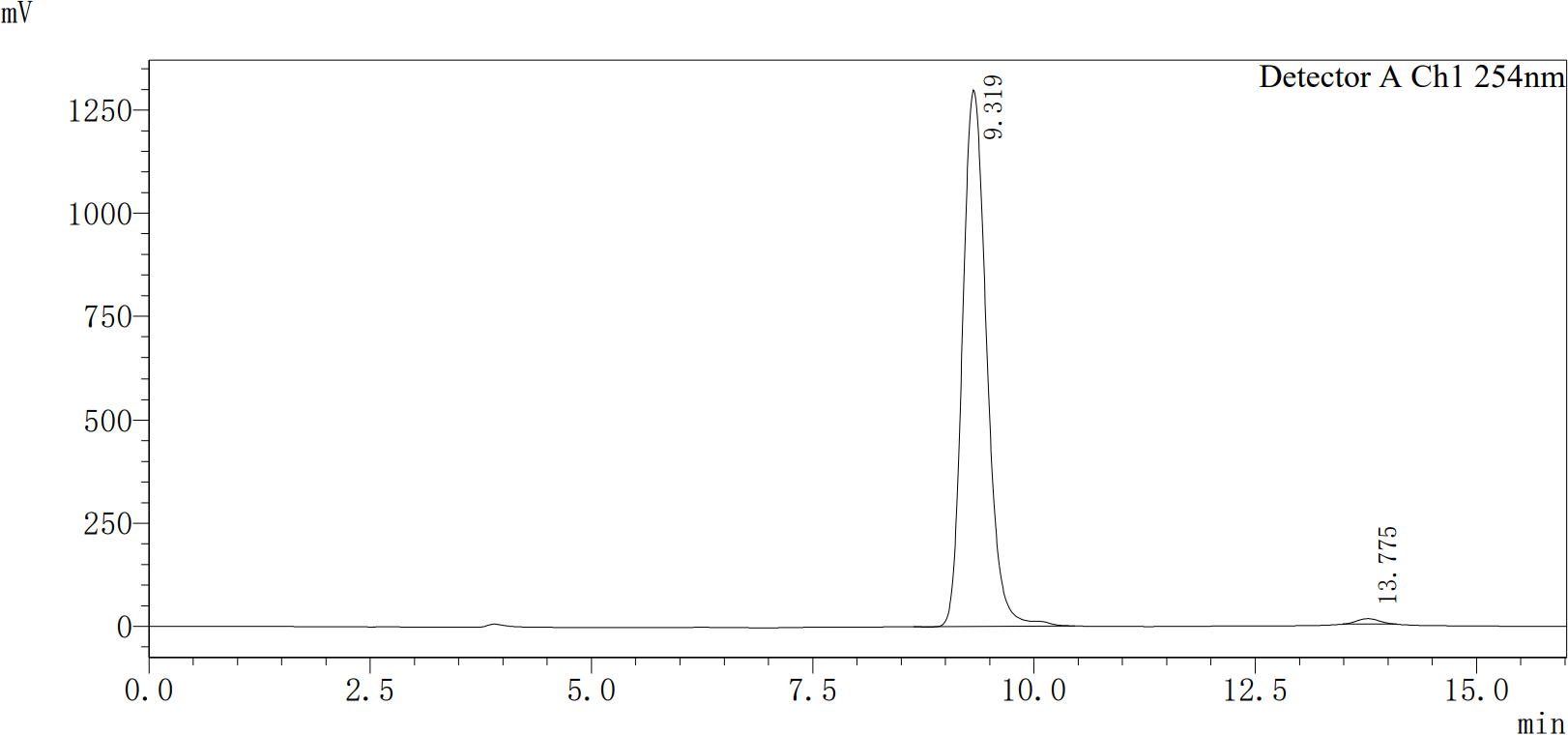
*

<Peak table>

Detector A Ch1 254nm

| Number | Retention time | Peak area | Peak area % |
| --- | --- | --- | --- |
| 1 | 9.319 | 24537322 | 99.045 |
| 2 | 13.775 | 236577 | 0.955 |
| Total |  | 24773900 | 100.000 |

Sample Name: **A6**

Column: Phenomenex Luna^®^ 5 μm C18(2) 100 Å 250 X 4.6 mm

Mobile phase: A: H_2_O; B: MeOH

Date: 2024-08-27

Injection volume: 15 uL

Flow rate: 0.5 mL/min

Method: 70% B for 0.5 min, 70% B to 100% B in 6 min gradient, 100% B for 9 min. stop at 16.01 min

*
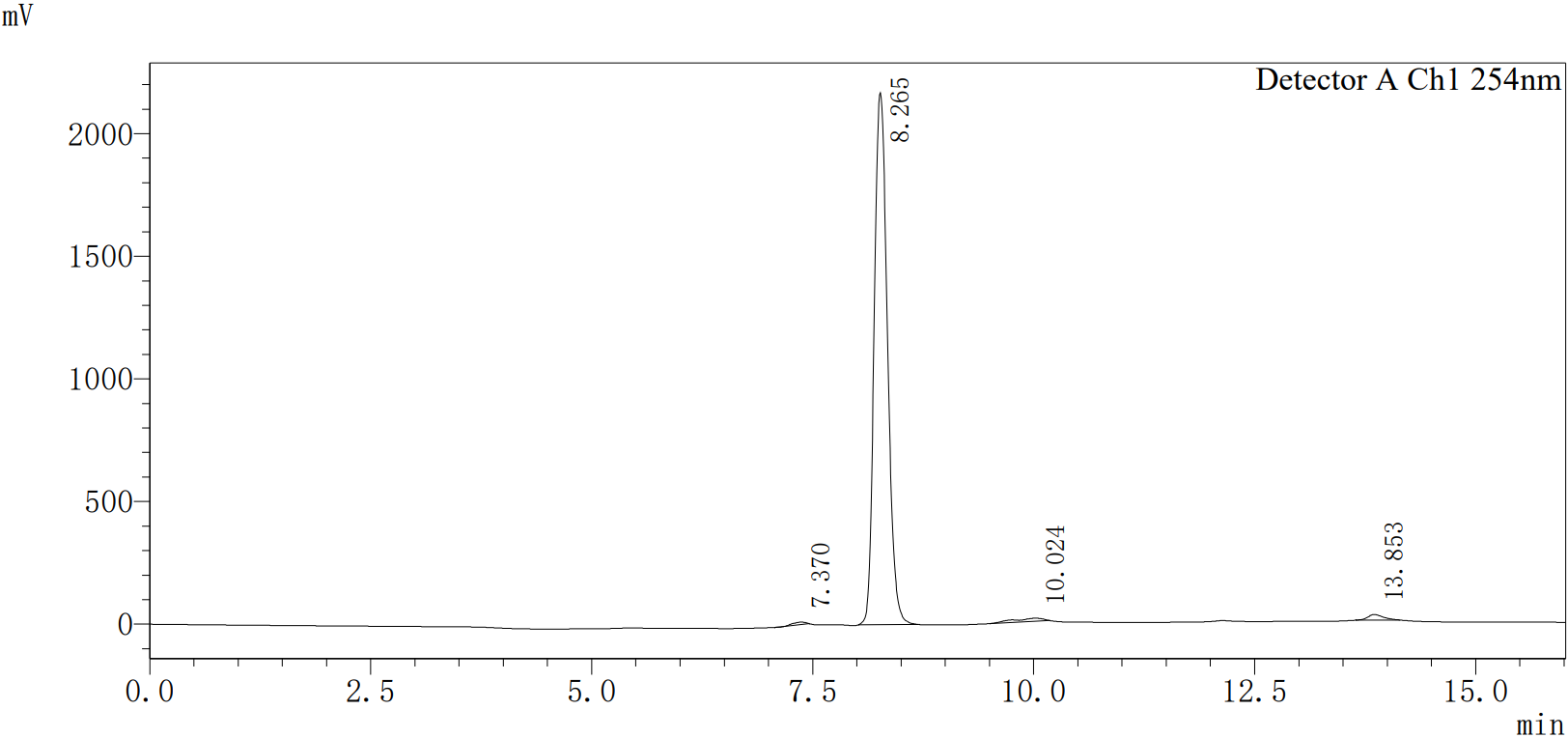
*

<Peak table>

Detector A Ch1 254nm

| Number | Retention time | Peak area | Peak area % |
| --- | --- | --- | --- |
| 1 | 7.370 | 98802 | 0.424 |
| 2 | 8.265 | 22606276 | 97.069 |
| 3 | 10.024 | 303658 | 1.304 |
| 4 | 13.853 | 280197 | 1.203 |
| Total |  | 23288934 | 100.000 |

Sample Name: **A7**

Column: Phenomenex Luna^®^ 5 μm C18(2) 100 Å 250 X 4.6 mm

Mobile phase: A: H_2_O; B: MeOH

Date: 2024-08-27

Injection volume: 15 uL

Flow rate: 0.5 mL/min

Method: 70% B for 0.5 min, 70% B to 100% B in 6 min gradient, 100% B for 9 min. stop at 16.01 min

*
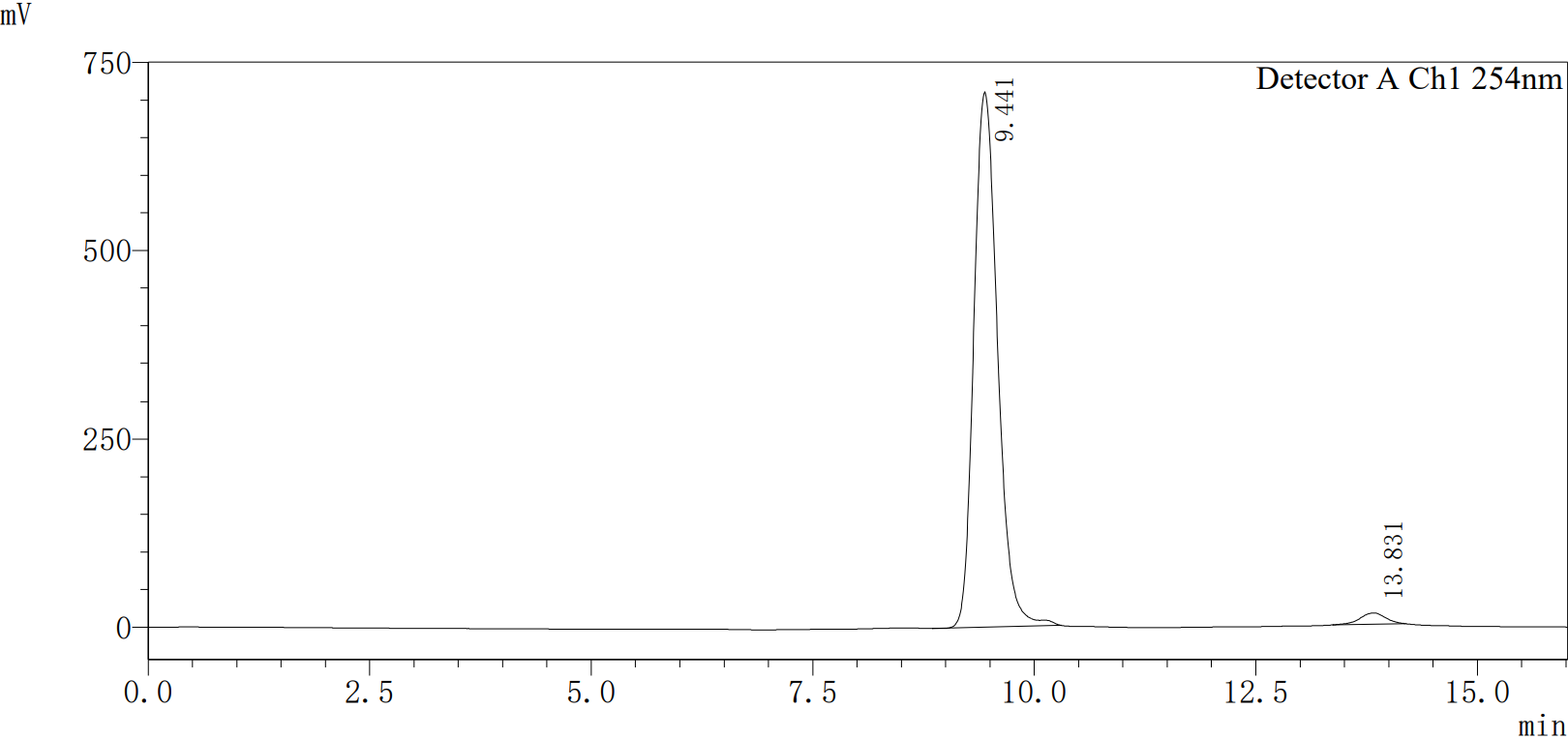
*

<Peak table>

Detector A Ch1 254nm

| Number | Retention time | Peak area | Peak area % |
| --- | --- | --- | --- |
| 1 | 9.441 | 13232131 | 97.757 |
| 2 | 13.831 | 303667 | 2.243 |
| Total |  | 13535798 | 100.000 |

Sample Name: **A8**

Column: Phenomenex Luna^®^ 5 μm C18(2) 100 Å 250 X 4.6 mm

Mobile phase: A: H_2_O; B: MeOH

Date: 2024-08-27

Injection volume: 15 uL

Flow rate: 0.5 mL/min

Method: 70% B for 0.5 min, 70% B to 100% B in 6 min gradient, 100% B for 9 min. stop at 16.01 min

*
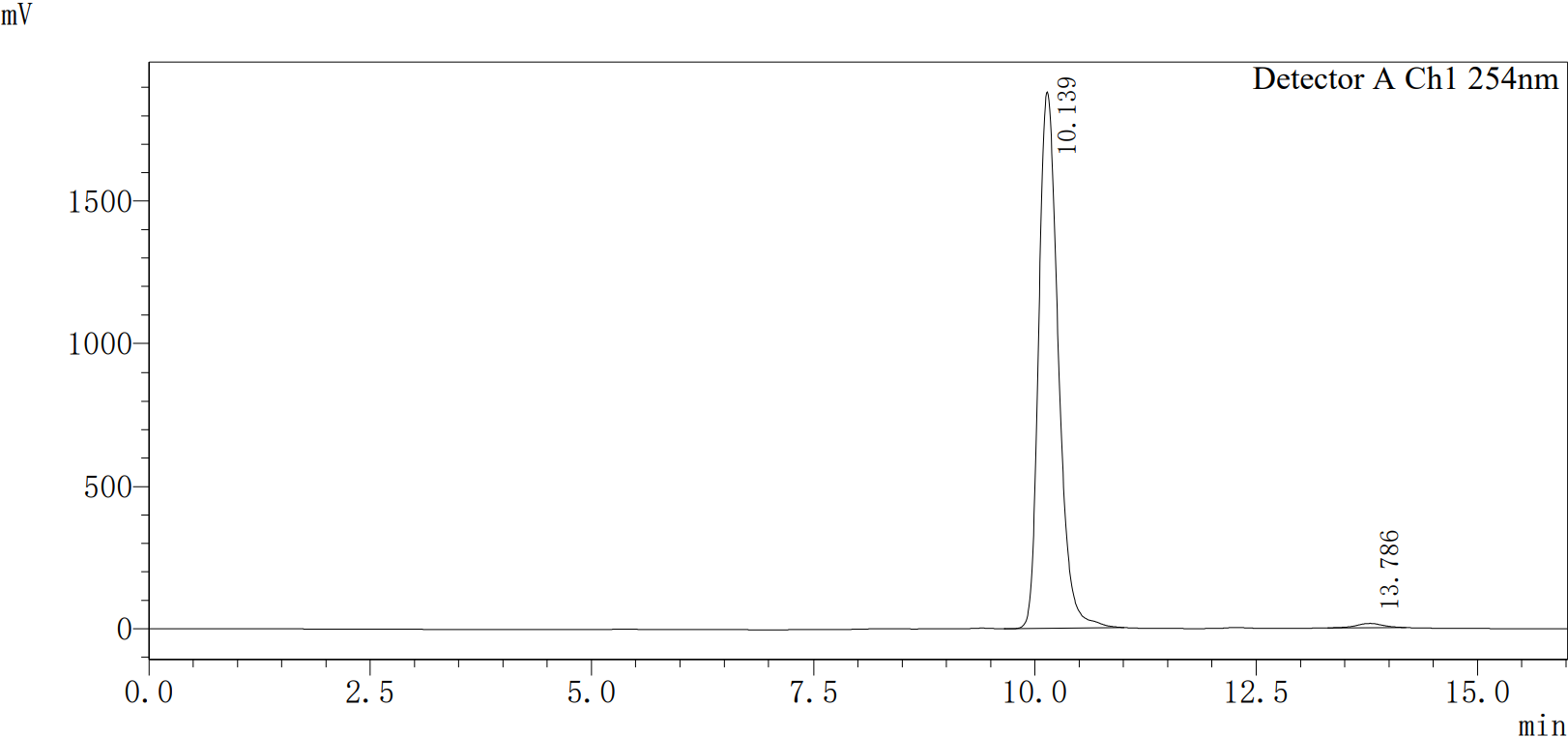
*

<Peak table>

Detector A Ch1 254nm

| Number | Retention time | Peak area | Peak area % |
| --- | --- | --- | --- |
| 1 | 10.139 | 28980544 | 98.918 |
| 2 | 13.786 | 317059 | 1.082 |
| Total |  | 29297603 | 100.000 |

Sample Name: **A9**

Column: Phenomenex Luna^®^ 5 μm C18(2) 100 Å 250 X 4.6 mm

Mobile phase: A: H_2_O; B: MeOH

Date: 2024-08-27

Injection volume: 15 uL

Flow rate: 0.5 mL/min

Method: 70% B for 0.5 min, 70% B to 100% B in 6 min gradient, 100% B for 9 min. stop at 16.01 min

*
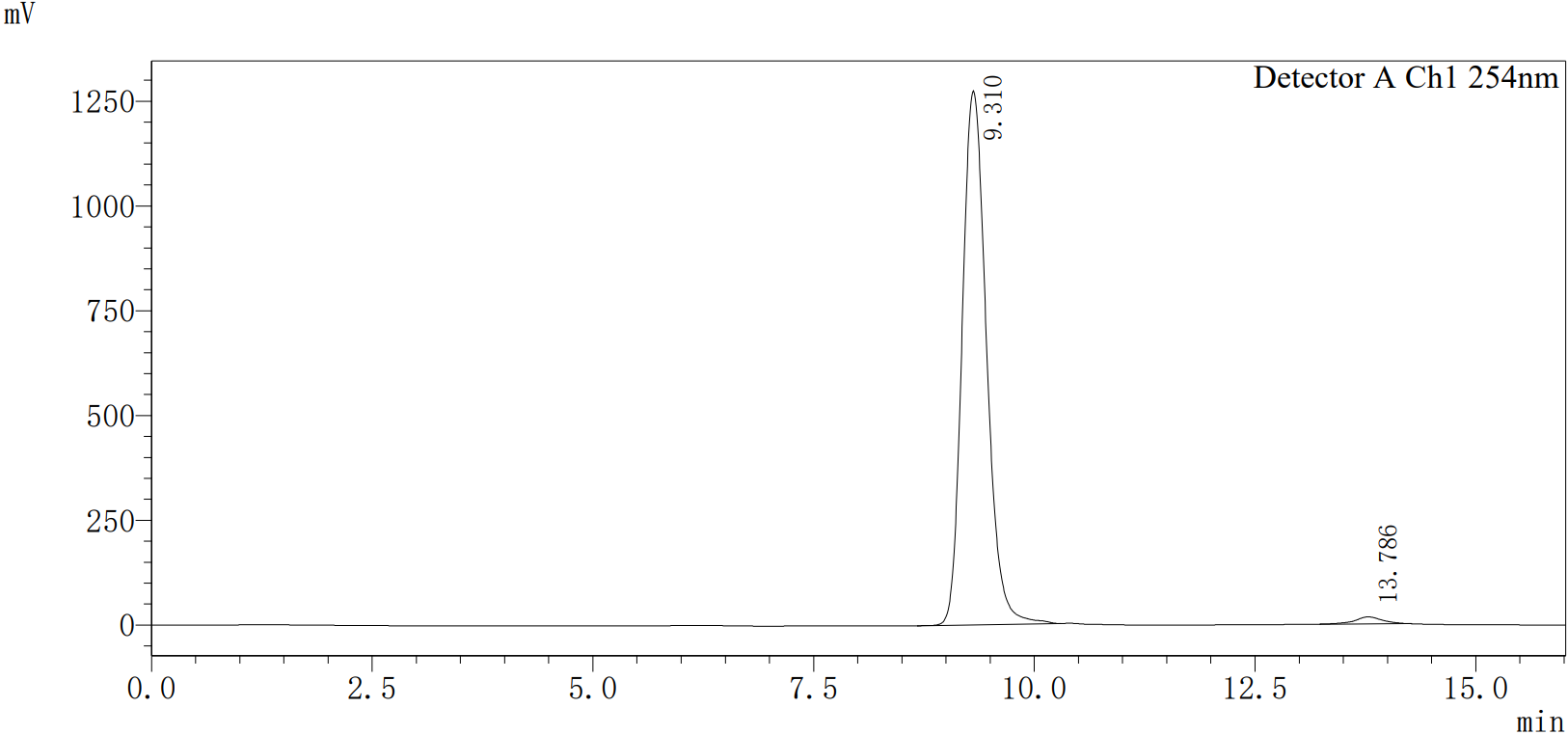
*

<Peak table>

Detector A Ch1 254nm

| Number | Retention time | Peak area | Peak area % |
| --- | --- | --- | --- |
| 1 | 9.310 | 24370716 | 98.499 |
| 2 | 13.786 | 371438 | 1.501 |
| Total |  | 24742153 | 100.000 |

Sample Name: **A10**

Column: Phenomenex Luna^®^ 5 μm C18(2) 100 Å 250 X 4.6 mm

Mobile phase: A: H_2_O; B: MeOH

Date: 2024-08-27

Injection volume: 15 uL

Flow rate: 0.5 mL/min

Method: 70% B for 0.5 min, 70% B to 100% B in 6 min gradient, 100% B for 9 min. stop at 16.01 min

*
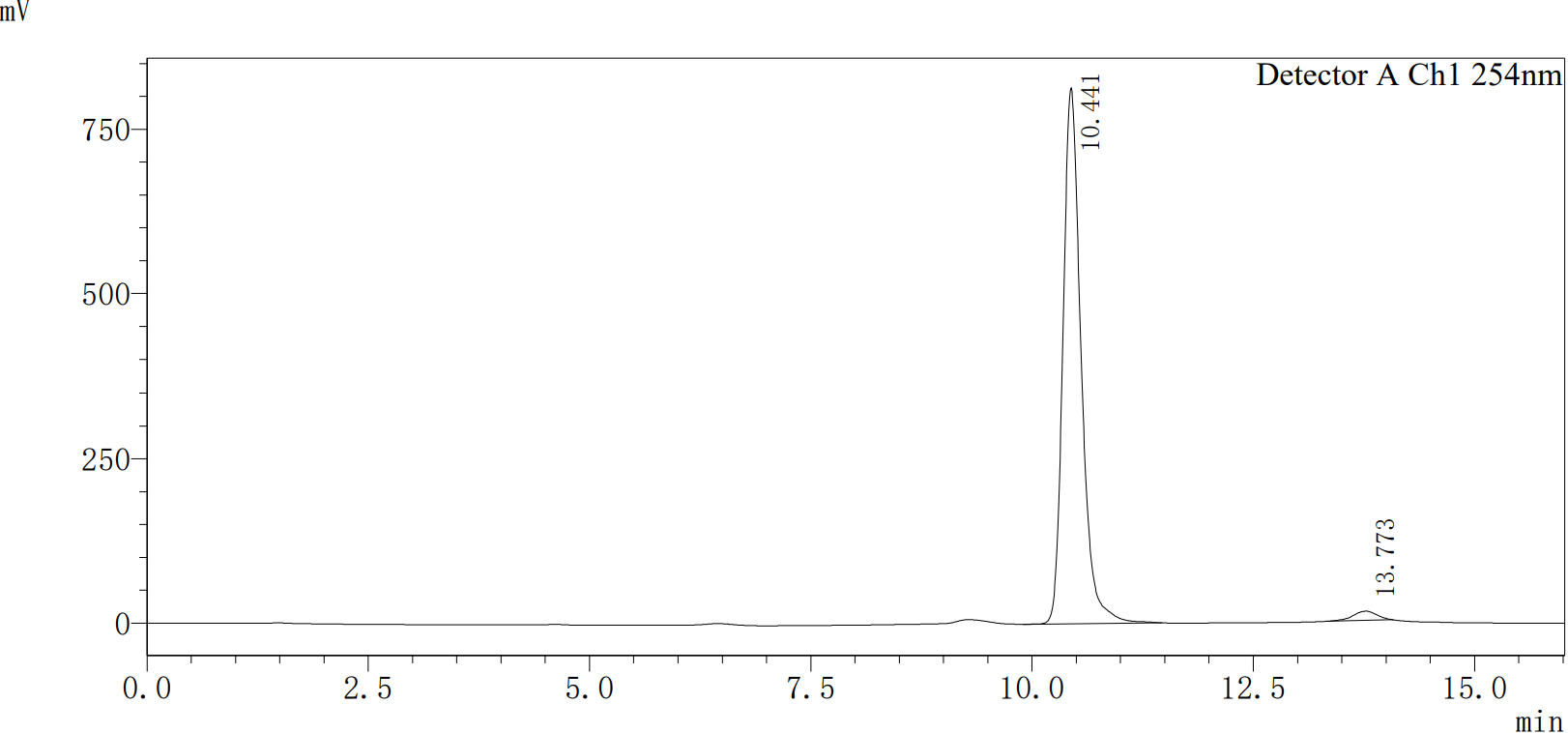
*

<Peak table>

Detector A Ch1 254nm

| Number | Retention time | Peak area | Peak area % |
| --- | --- | --- | --- |
| 1 | 10.441 | 11593869 | 97.751 |
| 2 | 13.773 | 266736 | 2.249 |
| Total |  | 11860605 | 100.000 |

Sample Name: **A11**

Column: Phenomenex Luna^®^ 5 μm C18(2) 100 Å 250 X 4.6 mm

Mobile phase: A: H_2_O; B: MeOH

Date: 2024-08-27

Injection volume: 15 uL

Flow rate: 0.5 mL/min

Method: 70% B for 0.5 min, 70% B to 100% B in 6 min gradient, 100% B for 9 min. stop at 16.01 min


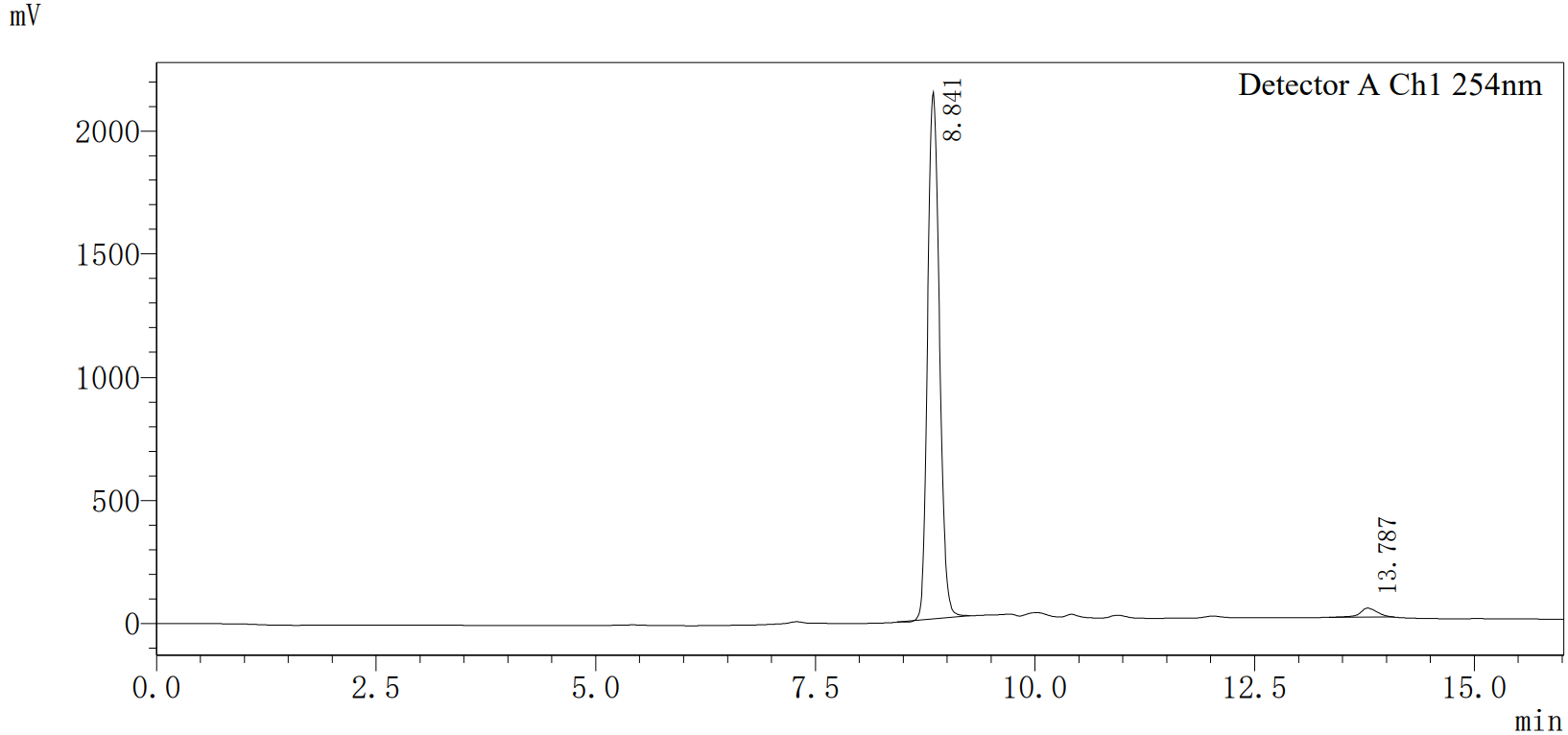


<Peak table>

Detector A Ch1 254nm

| Number | Retention time | Peak area | Peak area % |
| --- | --- | --- | --- |
| 1 | 8.841 | 20062787 | 97.489 |
| 2 | 13.787 | 516827 | 2.511 |
| Total |  | 20579614 | 100.000 |

Sample Name: **A12**

Column: Phenomenex Luna^®^ 5 μm C18(2) 100 Å 250 X 4.6 mm

Mobile phase: A: H_2_O; B: MeOH

Date: 2024-08-27

Injection volume: 15 uL

Flow rate: 0.5 mL/min

Method: 70% B for 0.5 min, 70% B to 100% B in 6 min gradient, 100% B for 9 min. stop at 16.01 min

*
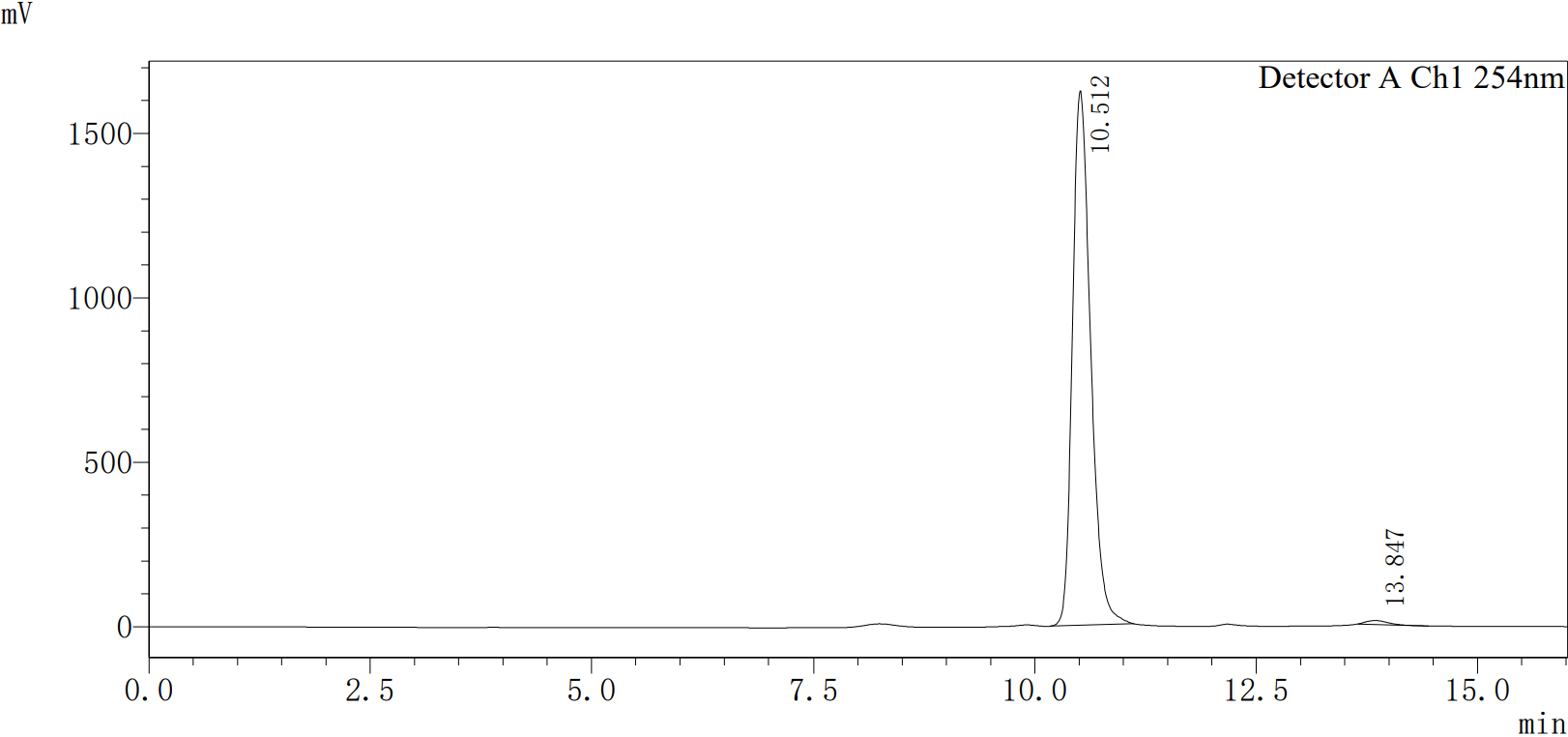
*

<Peak table>

Detector A Ch1 254nm

| Number | Retention time | Peak area | Peak area % |
| --- | --- | --- | --- |
| 1 | 10.512 | 23230625 | 99.149 |
| 2 | 13.847 | 199488 | 0.851 |
| Total |  | 23430113 | 100.000 |

Sample Name: **B1**

Column: Phenomenex Luna^®^ 5 μm C18(2) 100 Å 250 X 4.6 mm

Mobile phase: A: H_2_O; B: MeOH

Date: 2024-08-27

Injection volume: 15 uL

Flow rate: 0.5 mL/min

Method: 70% B for 0.5 min, 70% B to 100% B in 6 min gradient, 100% B for 9 min. stop at 16.01 min

*
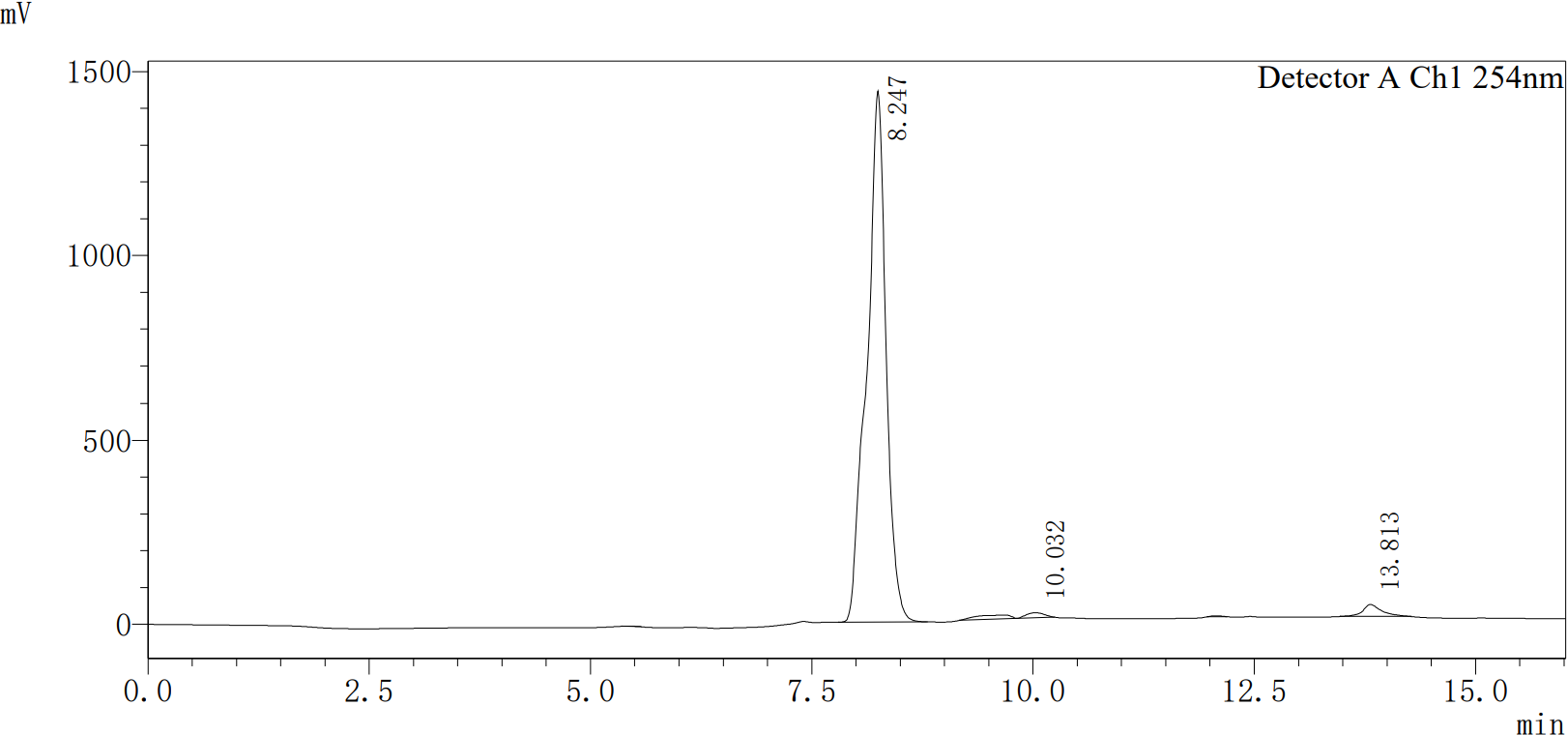
*

<Peak table>

Detector A Ch1 254nm

| Number | Retention time | Peak area | Peak area % |
| --- | --- | --- | --- |
| 1 | 8.247 | 21695692 | 95.509 |
| 2 | 10.032 | 16927 | 2.311 |
| 3 | 13.813 | 32687 | 2.180 |
| Total |  | 23430113 | 100.000 |

Sample Name: **B2**

Column: Phenomenex Luna^®^ 5 μm C18(2) 100 Å 250 X 4.6 mm

Mobile phase: A: H_2_O; B: MeOH

Date: 2024-08-27

Injection volume: 15 uL

Flow rate: 0.5 mL/min

Method: 70% B for 0.5 min, 70% B to 100% B in 6 min gradient, 100% B for 9 min. stop at 16.01 min

*
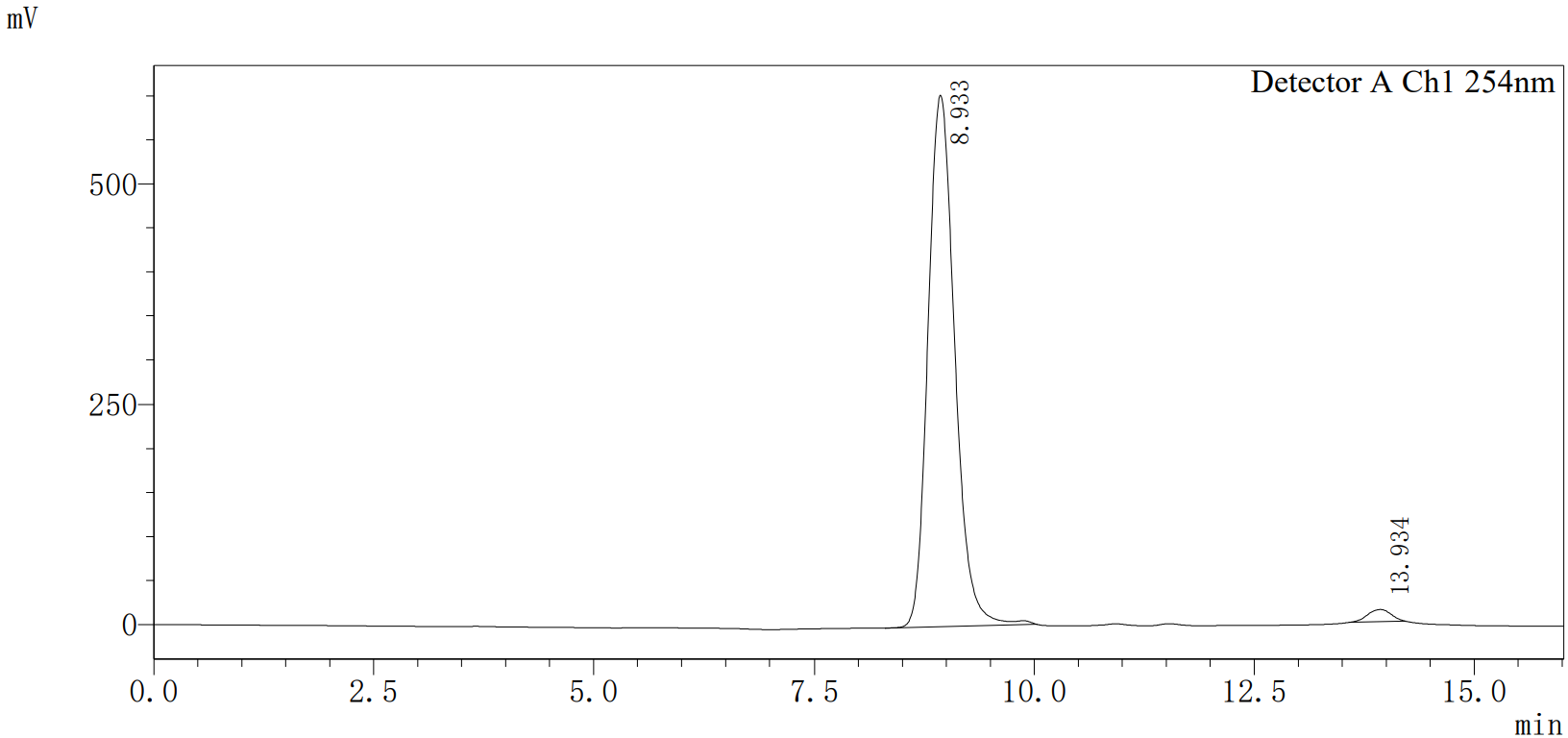
*

<Peak table>

Detector A Ch1 254nm

| Number | Retention time | Peak area | Peak area % |
| --- | --- | --- | --- |
| 1 | 8.933 | 12763505 | 98.001 |
| 2 | 13.934 | 260372 | 1.999 |
| Total |  | 13023877 | 100.000 |

Sample Name: **B3**

Column: Phenomenex Luna^®^ 5 μm C18(2) 100 Å 250 X 4.6 mm

Mobile phase: A: H_2_O; B: MeOH

Date: 2024-08-27

Injection volume: 15 uL

Flow rate: 0.5 mL/min

Method: 70% B for 0.5 min, 70% B to 100% B in 6 min gradient, 100% B for 9 min. stop at 16.01 min


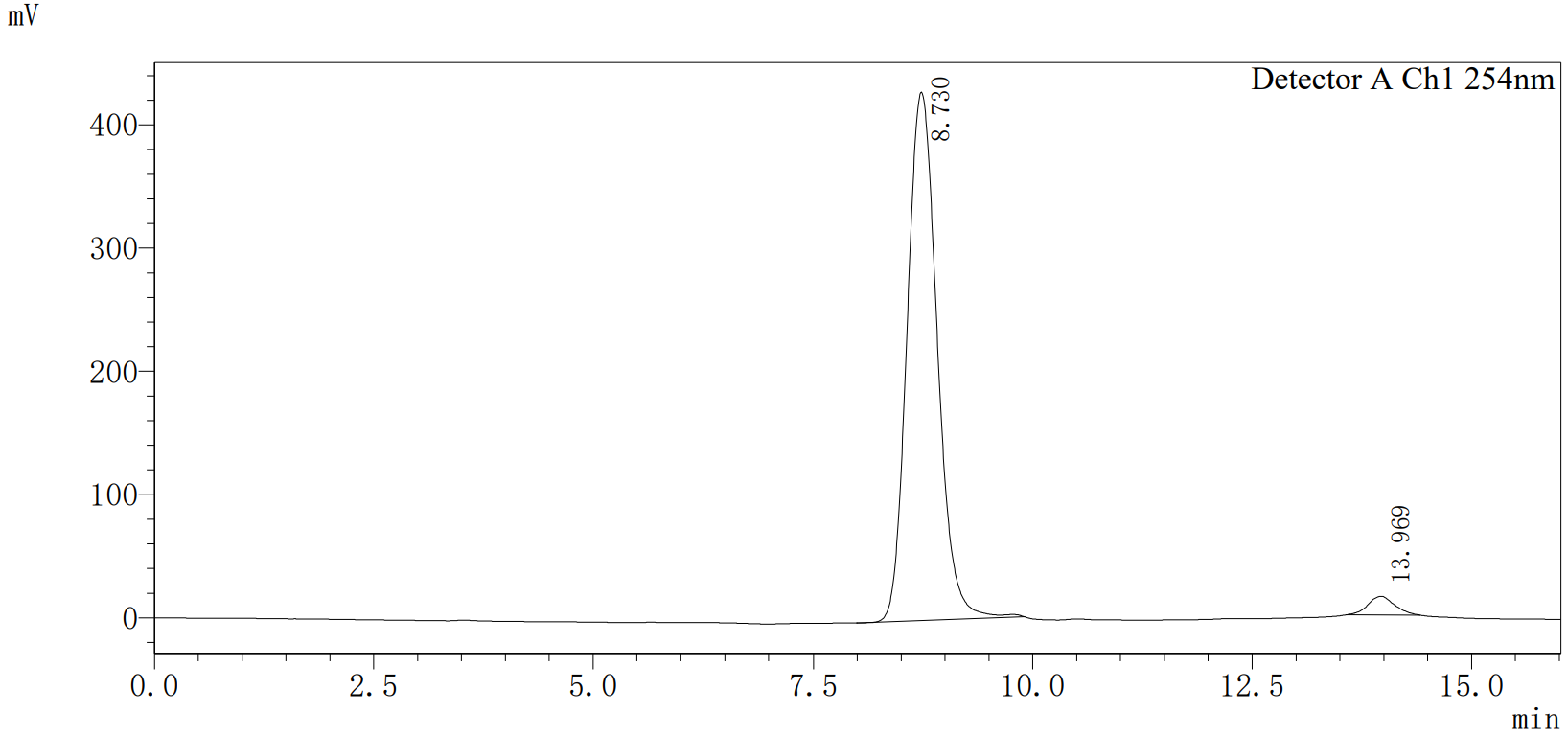


<Peak table>

Detector A Ch1 254nm

| Number | Retention time | Peak area | Peak area % |
| --- | --- | --- | --- |
| 1 | 8.730 | 10254654 | 96.965 |
| 2 | 13.969 | 321006 | 3.035 |
| Total |  | 13665967 | 100.000 |

Sample Name: **B4**

Column: Phenomenex Luna^®^ 5 μm C18(2) 100 Å 250 X 4.6 mm

Mobile phase: A: H_2_O; B: MeOH

Date: 2024-08-27

Injection volume: 15 uL

Flow rate: 0.5 mL/min

Method: 70% B for 0.5 min, 70% B to 100% B in 6 min gradient, 100% B for 9 min. stop at 16.01 min

*
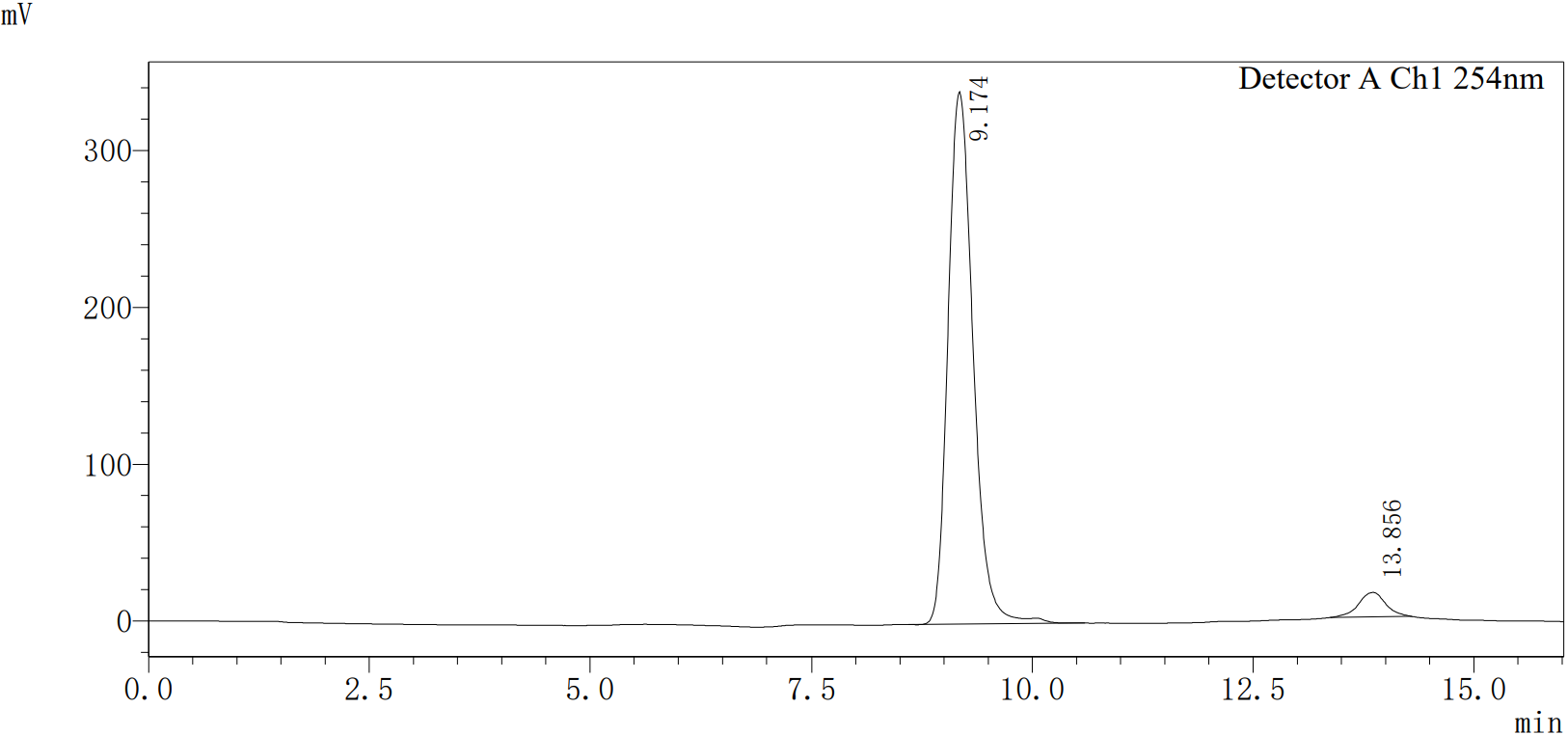
*

<Peak table>

Detector A Ch1 254nm

| Number | Retention time | Peak area | Peak area % |
| --- | --- | --- | --- |
| 1 | 9.174 | 6716623 | 95.228 |
| 2 | 13.856 | 336600 | 4.772 |
| Total |  | 7053223 | 100.000 |

Sample Name: **B5**

Column: Phenomenex Luna^®^ 5 μm C18(2) 100 Å 250 X 4.6 mm

Mobile phase: A: H_2_O; B: MeOH

Date: 2024-08-27

Injection volume: 15 uL

Flow rate: 0.5 mL/min

Method: 70% B for 0.5 min, 70% B to 100% B in 6 min gradient, 100% B for 9 min. stop at 16.01 min

*
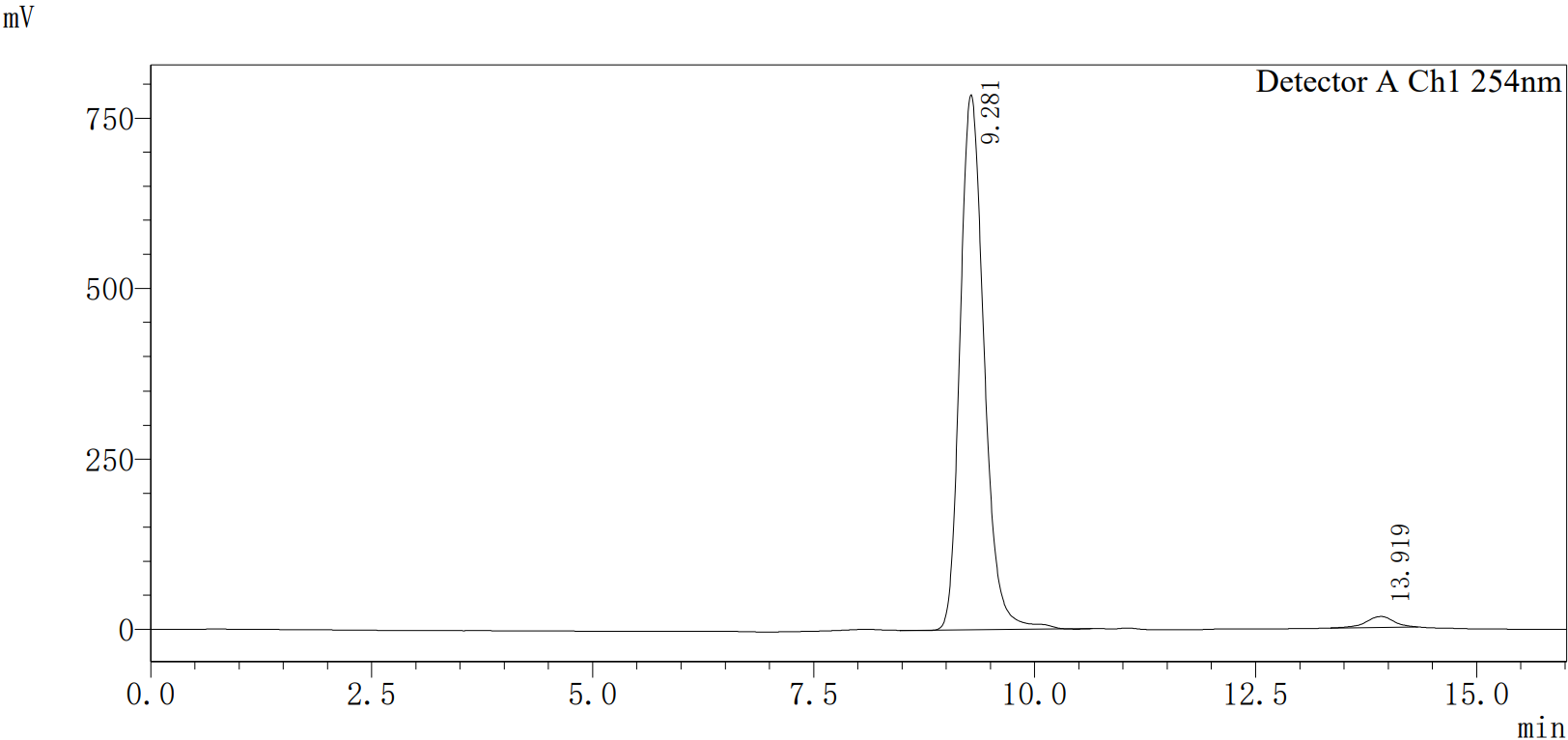
*

<Peak table>

Detector A Ch1 254nm

| Number | Retention time | Peak area | Peak area % |
| --- | --- | --- | --- |
| 1 | 9.281 | 14862222 | 97.713 |
| 2 | 13.919 | 347885 | 2.287 |
| Total |  | 15210108 | 100.000 |

Sample Name: **B6**

Column: Phenomenex Luna^®^ 5 μm C18(2) 100 Å 250 X 4.6 mm

Mobile phase: A: H_2_O; B: MeOH

Date: 2024-08-27

Injection volume: 15 uL

Flow rate: 0.5 mL/min

Method: 70% B for 0.5 min, 70% B to 100% B in 6 min gradient, 100% B for 9 min. stop at 16.01 min

*
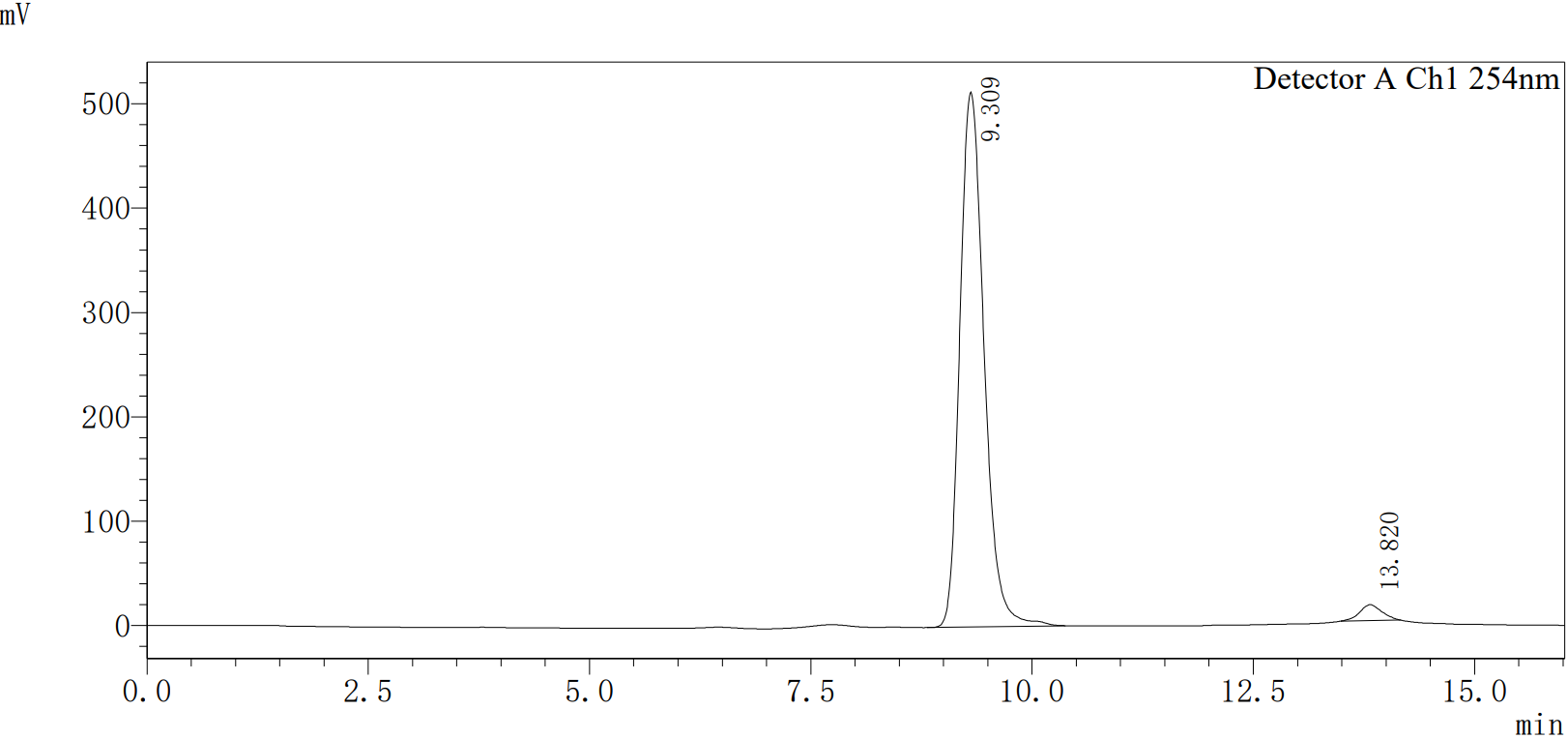
*

<Peak table>

Detector A Ch1 254nm

| Number | Retention time | Peak area | Peak area % |
| --- | --- | --- | --- |
| 1 | 9.309 | 9765209 | 97.366 |
| 2 | 13.820 | 264203 | 2.634 |
| Total |  | 10029411 | 100.000 |

Sample Name: **B7**

Column: Phenomenex Luna^®^ 5 μm C18(2) 100 Å 250 X 4.6 mm

Mobile phase: A: H_2_O; B: MeOH

Date: 2024-08-27

Injection volume: 15 uL

Flow rate: 0.5 mL/min

Method: 70% B for 0.5 min, 70% B to 100% B in 6 min gradient, 100% B for 9 min. stop at 16.01 min

*
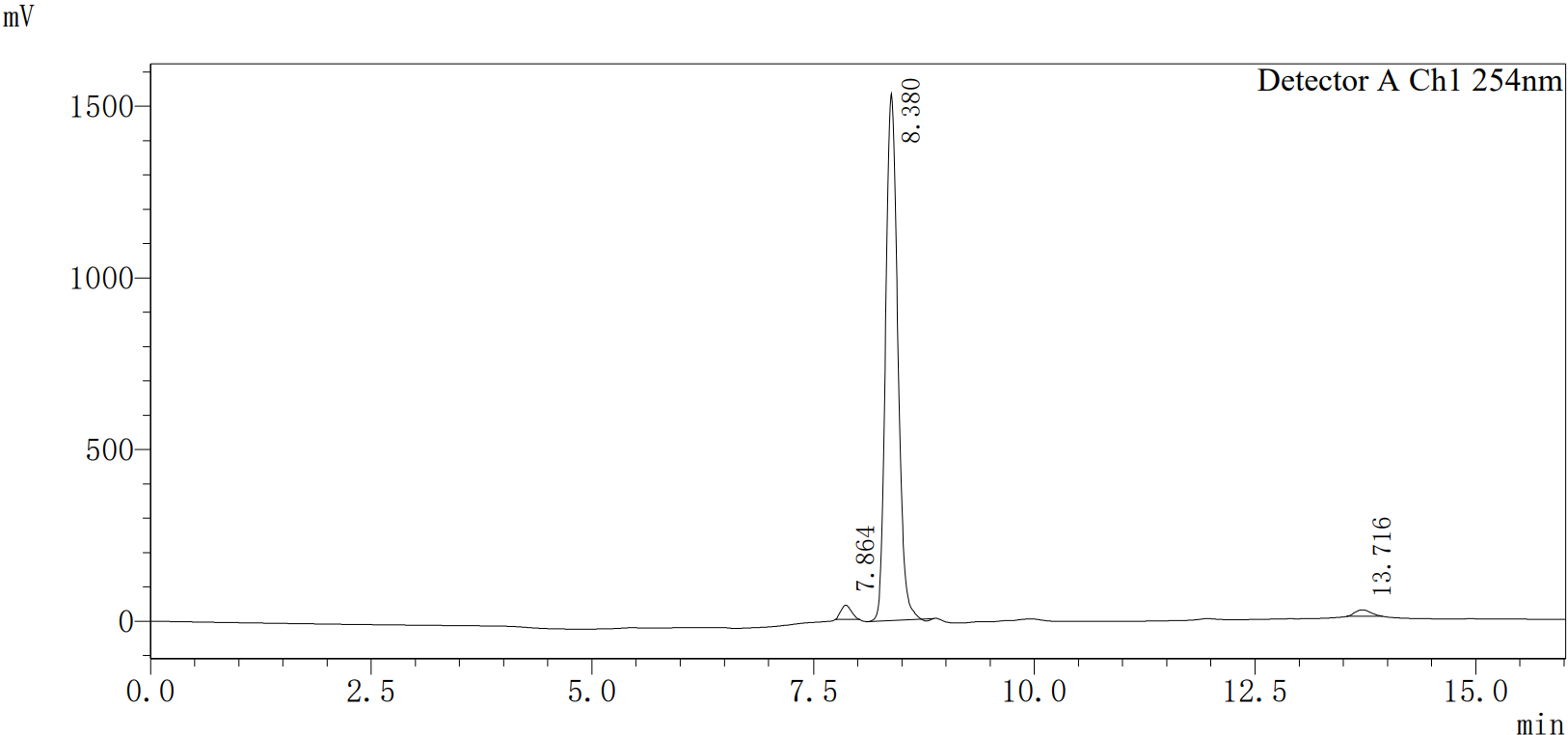
*

<Peak table>

Detector A Ch1 254nm

| Number | Retention time | Peak area | Peak area % |
| --- | --- | --- | --- |
| 1 | 7.864 | 340540 | 2.232 |
| 2 | 8.380 | 14685814 | 96.270 |
| 3 | 13.716 | 228514 | 1.498 |
| Total |  | 15254867 | 100.000 |

Sample Name: **B8**

Column: Phenomenex Luna^®^ 5 μm C18(2) 100 Å 250 X 4.6 mm

Mobile phase: A: H_2_O; B: MeOH

Date: 2024-08-27

Injection volume: 15 uL

Flow rate: 0.5 mL/min

Method: 70% B for 0.5 min, 70% B to 100% B in 6 min gradient, 100% B for 9 min. stop at 16.01 min

*
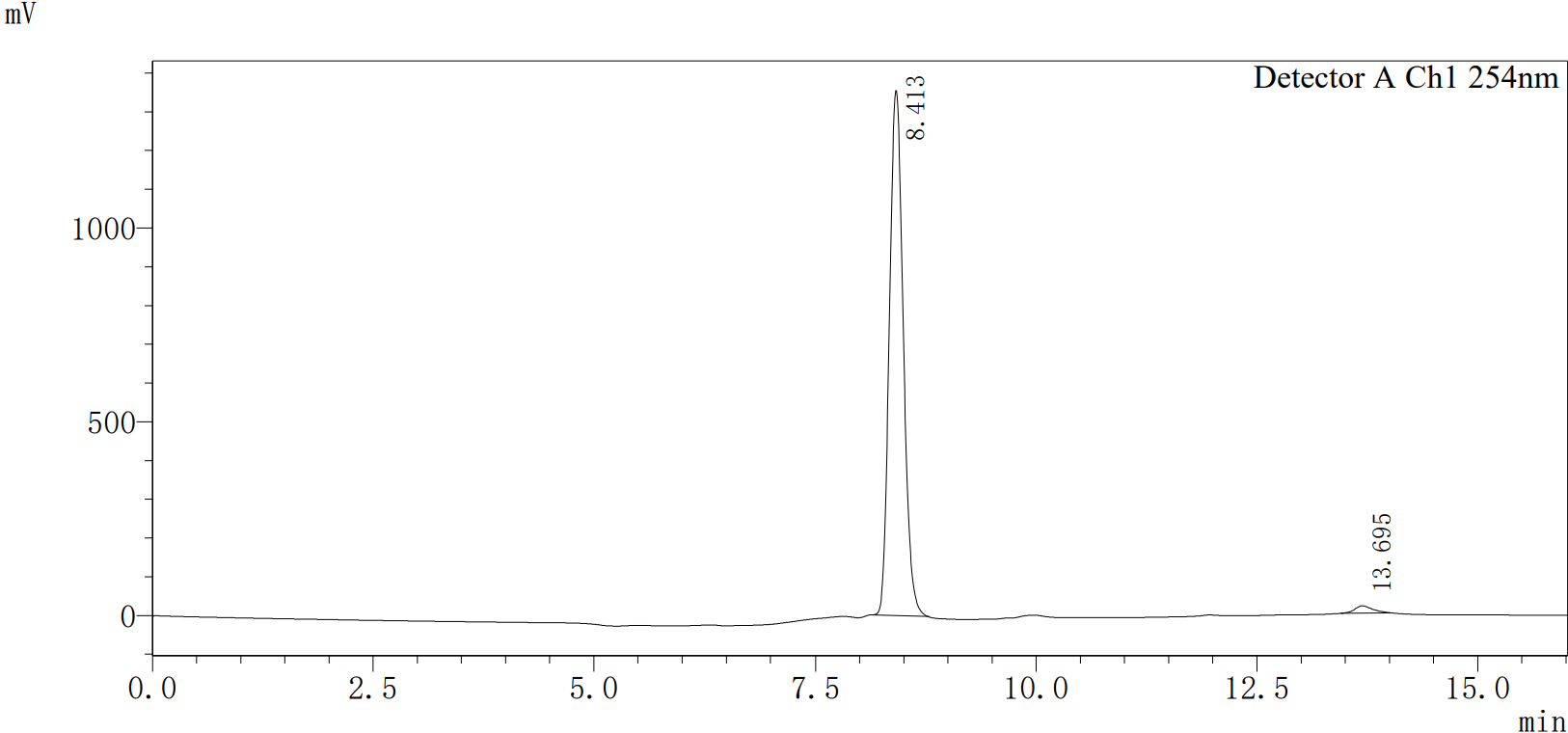
*

<Peak table>

Detector A Ch1 254nm

| Number | Retention time | Peak area | Peak area % |
| --- | --- | --- | --- |
| 1 | 8.413 | 14861996 | 98.274 |
| 2 | 13.695 | 260975 | 1.726 |
| Total |  | 15122970 | 100.000 |

Sample Name: **B9**

Column: Phenomenex Luna^®^ 5 μm C18(2) 100 Å 250 X 4.6 mm

Mobile phase: A: H_2_O; B: MeOH

Date: 2024-08-27

Injection volume: 15 uL

Flow rate: 0.5 mL/min

Method: 70% B for 0.5 min, 70% B to 100% B in 6 min gradient, 100% B for 9 min. stop at 16.01 min

*
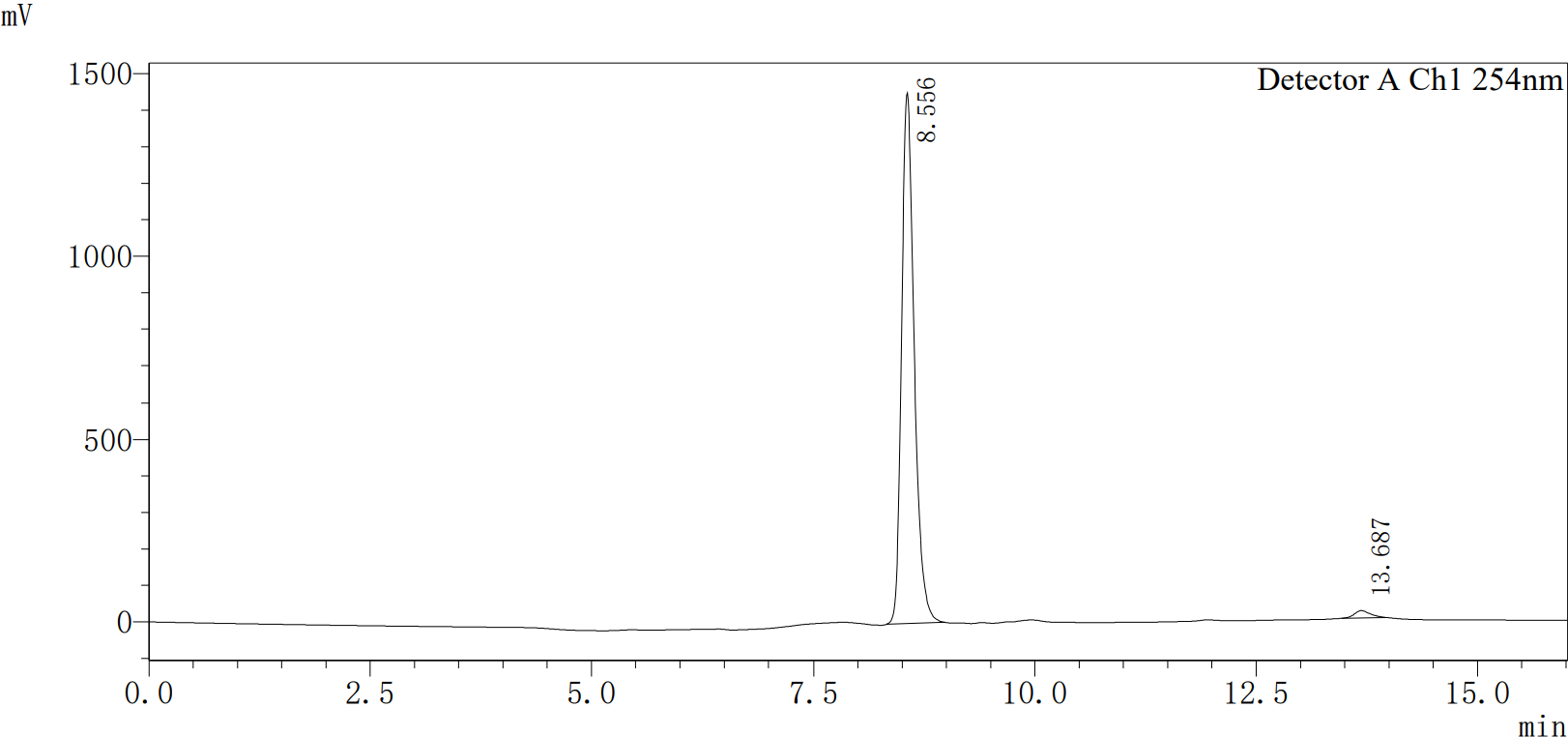
*

<Peak table>

Detector A Ch1 254nm

| Number | Retention time | Peak area | Peak area % |
| --- | --- | --- | --- |
| 1 | 8.556 | 14463941 | 98.257 |
| 2 | 13.687 | 256536 | 1.743 |
| Total |  | 14720477 | 100.000 |

Sample Name: **B10**

Column: Phenomenex Luna^®^ 5 μm C18(2) 100 Å 250 X 4.6 mm

Mobile phase: A: H_2_O; B: MeOH

Date: 2024-08-27

Injection volume: 15 uL

Flow rate: 0.5 mL/min

Method: 70% B for 0.5 min, 70% B to 100% B in 6 min gradient, 100% B for 9 min. stop at 16.01 min

*
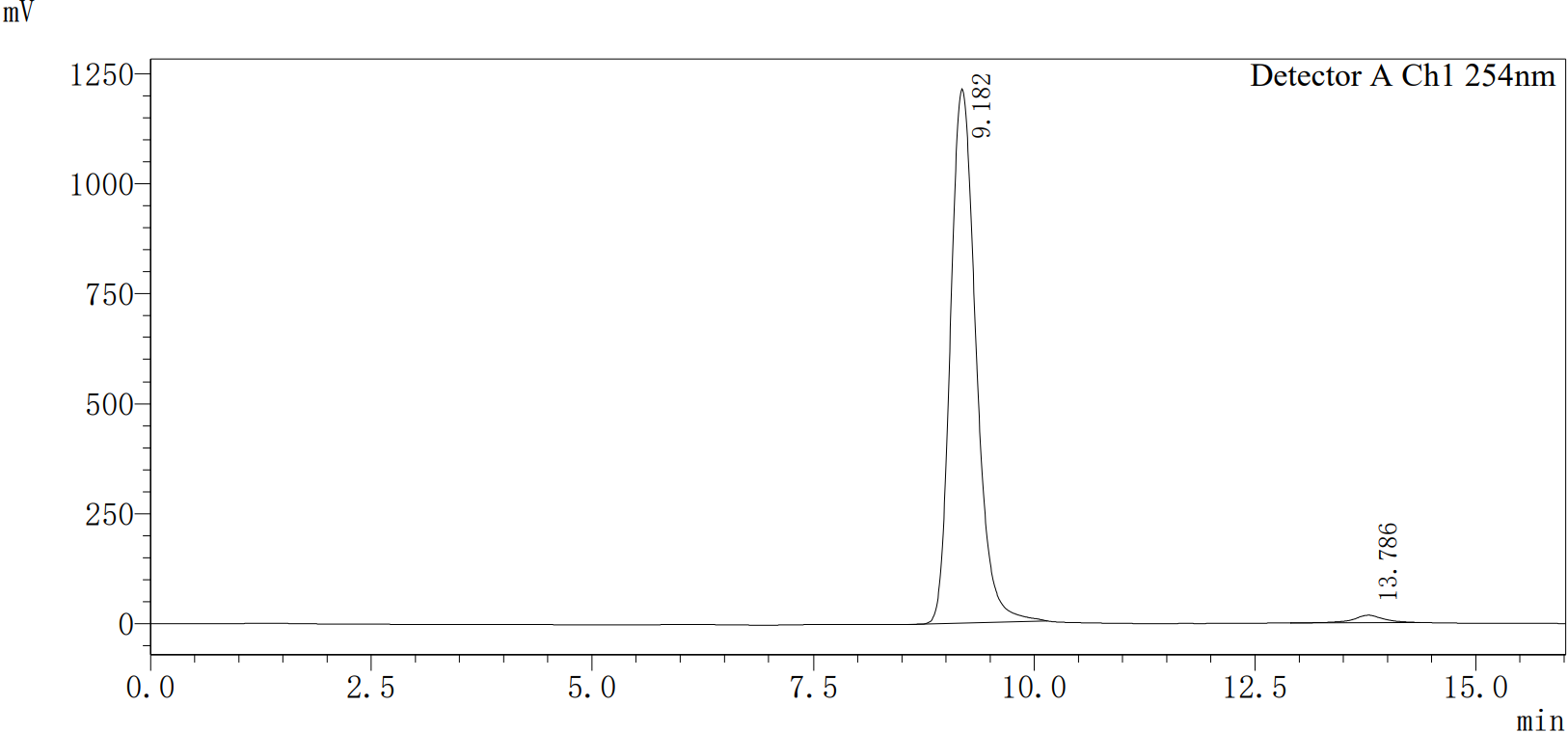
*

<Peak table>

Detector A Ch1 254nm

| Number | Retention time | Peak area | Peak area % |
| --- | --- | --- | --- |
| 1 | 9.182 | 24335686 | 98.366 |
| 2 | 13.786 | 404222 | 1.634 |
| Total |  | 24739908 | 100.000 |

Sample Name: **B11**

Column: Phenomenex Luna^®^ 5 μm C18(2) 100 Å 250 X 4.6 mm

Mobile phase: A: H_2_O; B: MeOH

Date: 2024-08-27

Injection volume: 15 uL

Flow rate: 0.5 mL/min

Method: 70% B for 0.5 min, 70% B to 100% B in 6 min gradient, 100% B for 9 min. stop at 16.01 min

*
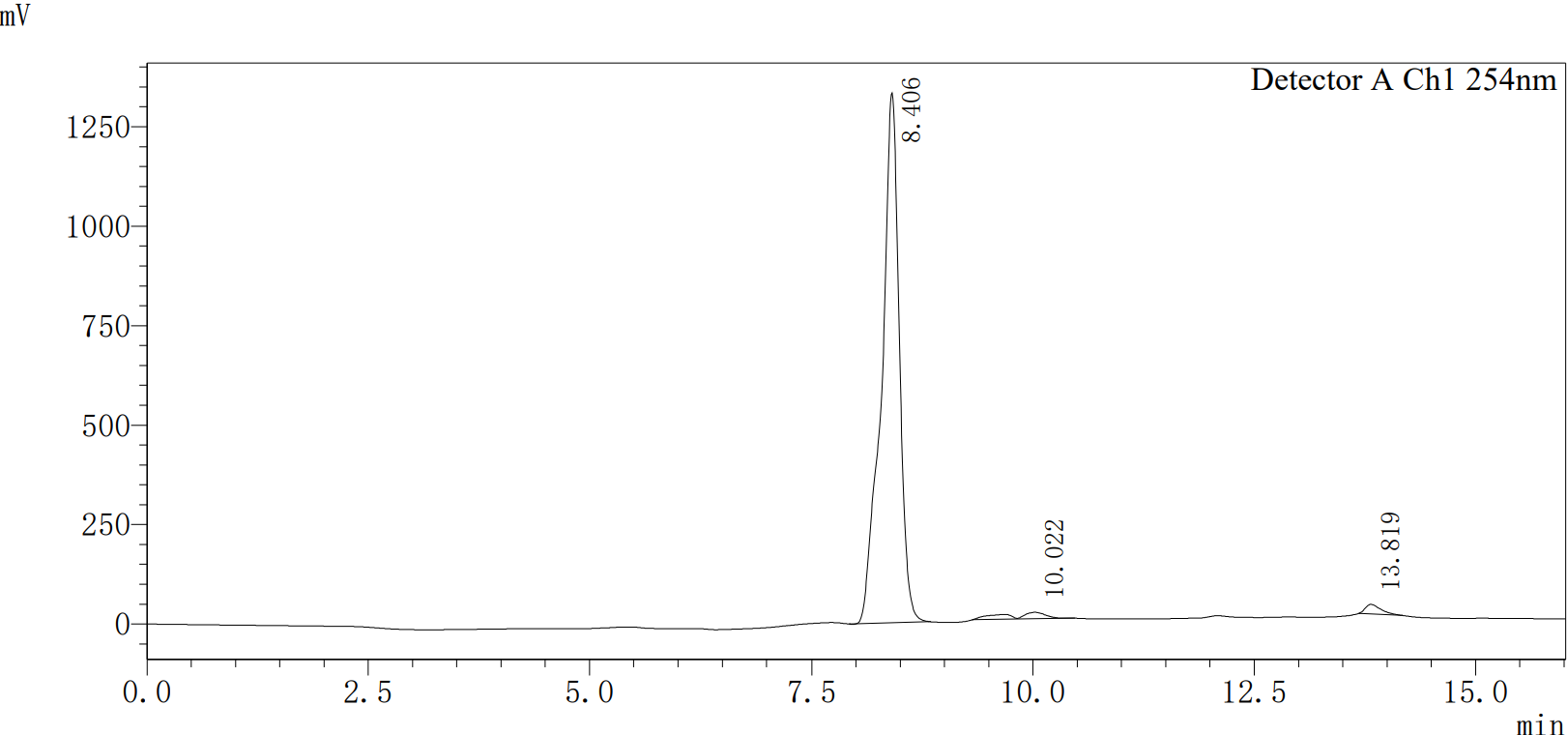
*

<Peak table>

Detector A Ch1 254nm

| Number | Retention time | Peak area | Peak area % |
| --- | --- | --- | --- |
| 1 | 8.406 | 18520539 | 95.836 |
| 2 | 10.022 | 511478 | 2.647 |
| 3 | 13.819 | 293159 | 1.517 |
| Total |  | 19325176 | 100.000 |

Sample Name: **B12**

Column: Phenomenex Luna^®^ 5 μm C18(2) 100 Å 250 X 4.6 mm

Mobile phase: A: H_2_O; B: MeOH

Date: 2024-08-27

Injection volume: 15 uL

Flow rate: 0.5 mL/min

Method: 70% B for 0.5 min, 70% B to 100% B in 6 min gradient, 100% B for 9 min. stop at 16.01 min

*
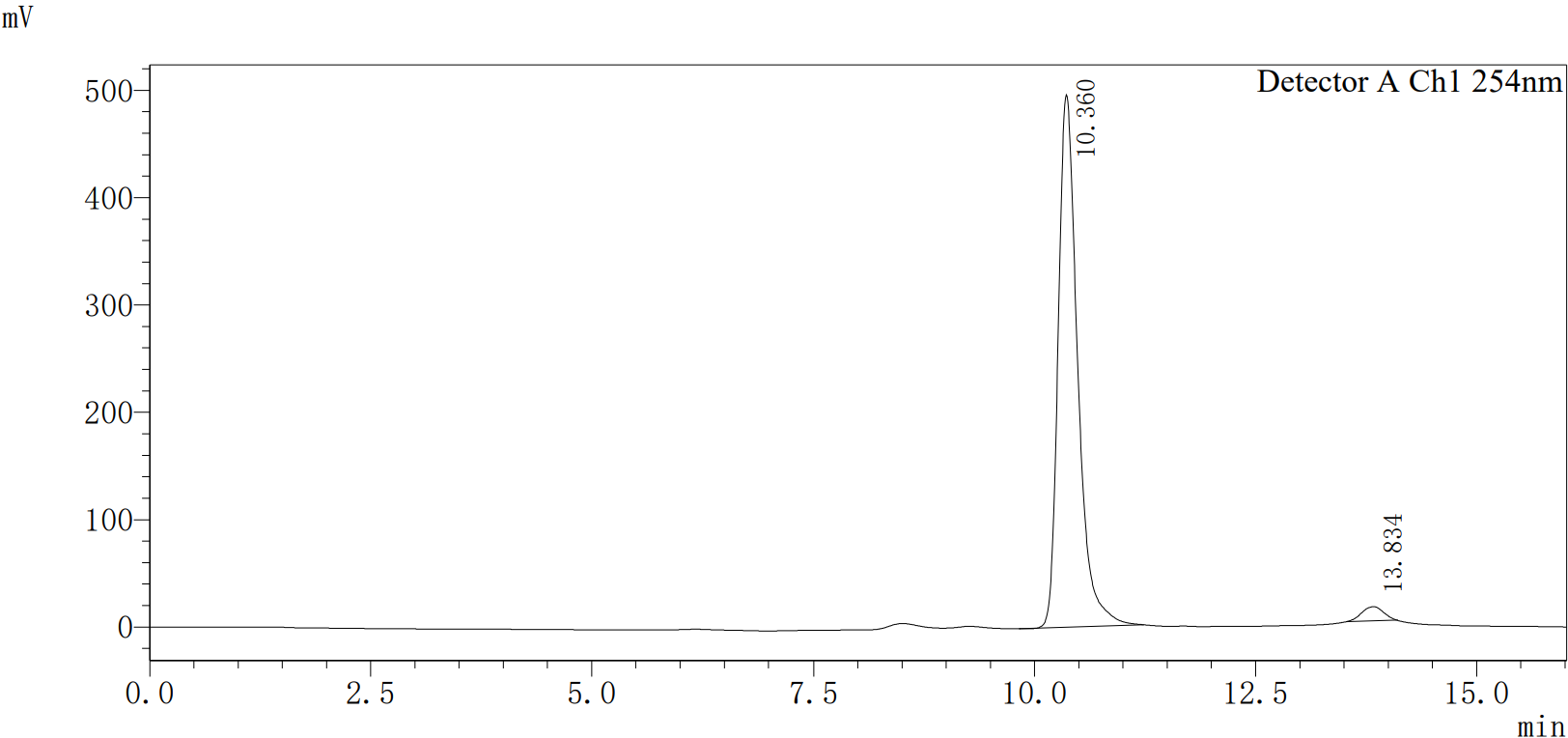
*

<Peak table>

Detector A Ch1 254nm

| Number | Retention time | Peak area | Peak area % |
| --- | --- | --- | --- |
| 1 | 10.360 | 7579972 | 97.065 |
| 2 | 13.834 | 229215 | 2.935 |
| Total |  | 7809187 | 100.000 |

Sample Name: **B13**

Column: Phenomenex Luna^®^ 5 μm C18(2) 100 Å 250 X 4.6 mm

Mobile phase: A: H_2_O; B: MeOH

Date: 2024-08-27

Injection volume: 15 uL

Flow rate: 0.5 mL/min

Method: 70% B for 0.5 min, 70% B to 100% B in 6 min gradient, 100% B for 9 min. stop at 16.01 min

*
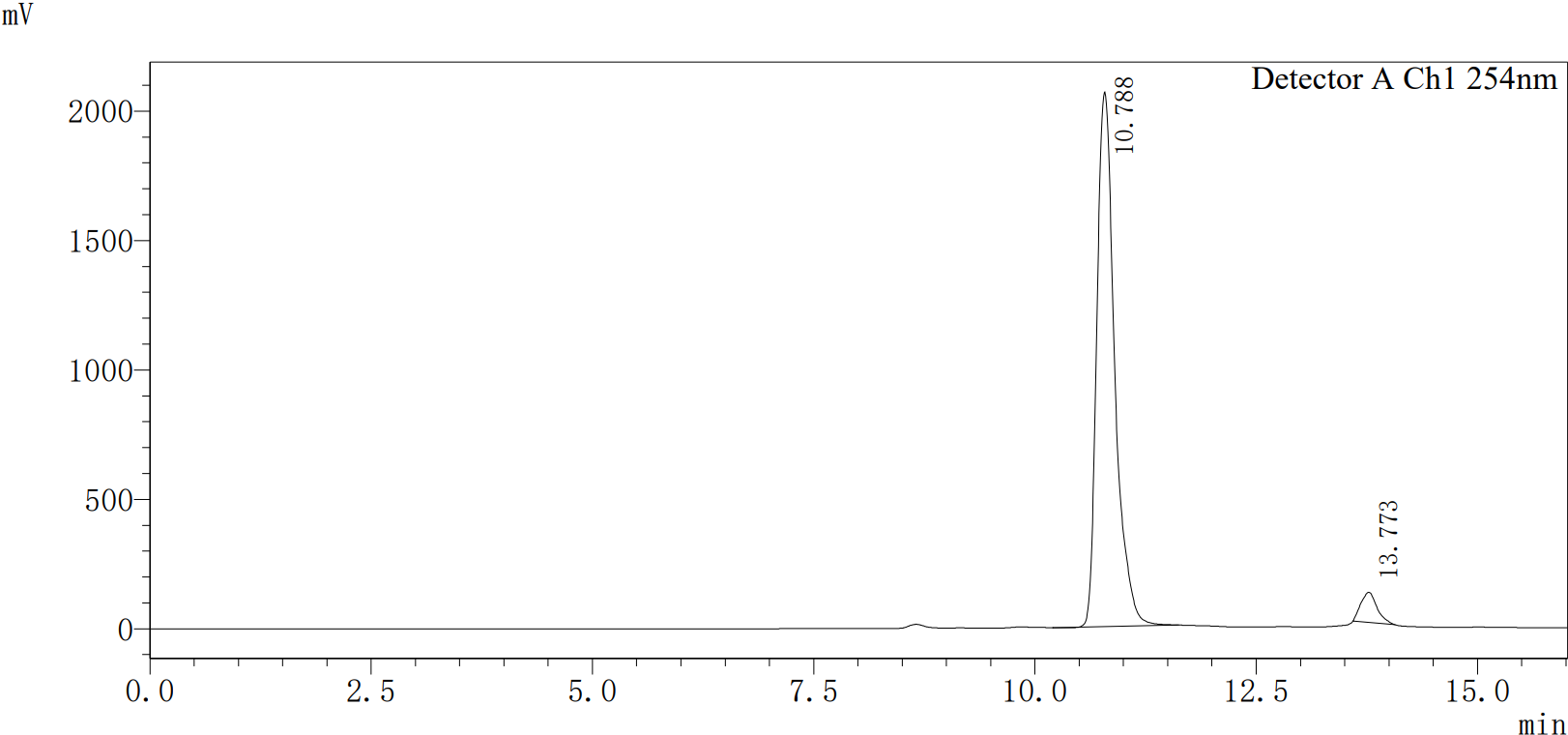
*

<Peak table>

Detector A Ch1 254nm

| Number | Retention time | Peak area | Peak area % |
| --- | --- | --- | --- |
| 1 | 10.788 | 29097935 | 95.173 |
| 2 | 13.773 | 1475853 | 4.827 |
| Total |  | 30573788 | 100.000 |

Sample Name: **B14**

Column: Phenomenex Luna^®^ 5 μm C18(2) 100 Å 250 X 4.6 mm

Mobile phase: A: H_2_O; B: MeOH

Date: 2024-08-27

Injection volume: 15 uL

Flow rate: 0.5 mL/min

Method: 70% B for 0.5 min, 70% B to 100% B in 6 min gradient, 100% B for 9 min. stop at 16.01 min

*
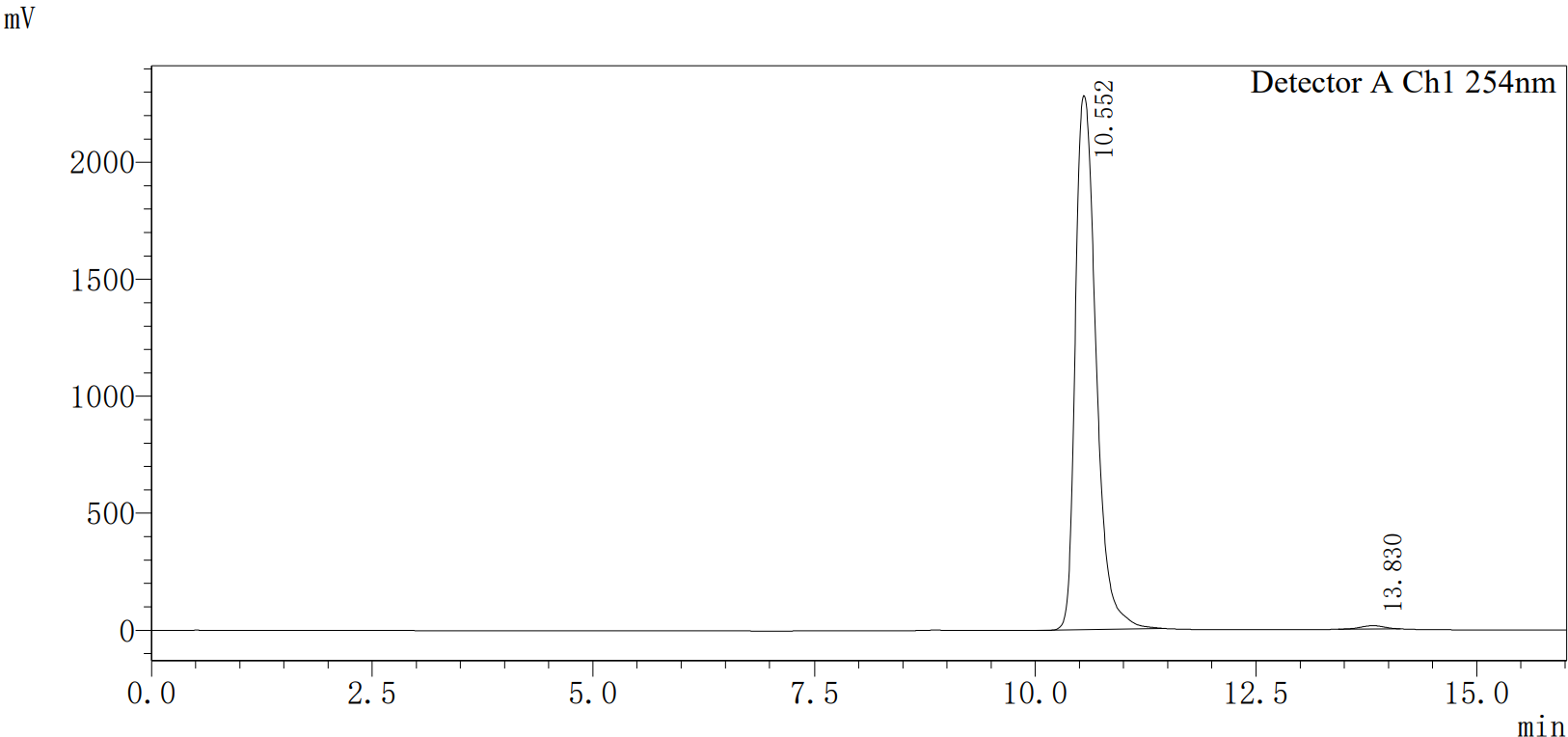
*

<Peak table>

Detector A Ch1 254nm

| Number | Retention time | Peak area | Peak area % |
| --- | --- | --- | --- |
| 1 | 10.552 | 37331130 | 99.298 |
| 2 | 13.830 | 264046 | 0.702 |
| Total |  | 37595176 | 100.000 |

**Part 5: Anti-proliferative effects of the drugs at different time points**

**Figure S2.** Anti-proliferative effects of **1** (Olaparib), **8** (AZD6738), **1** + **8** (1:1) and **B8** at 24 h, 48 h and 72 h, respectively in MDA-MB-231 and MDA-MB-468 cells.

**Part 6: Representative images of transwell invasion assay in MDA-MB-468 cells**

**
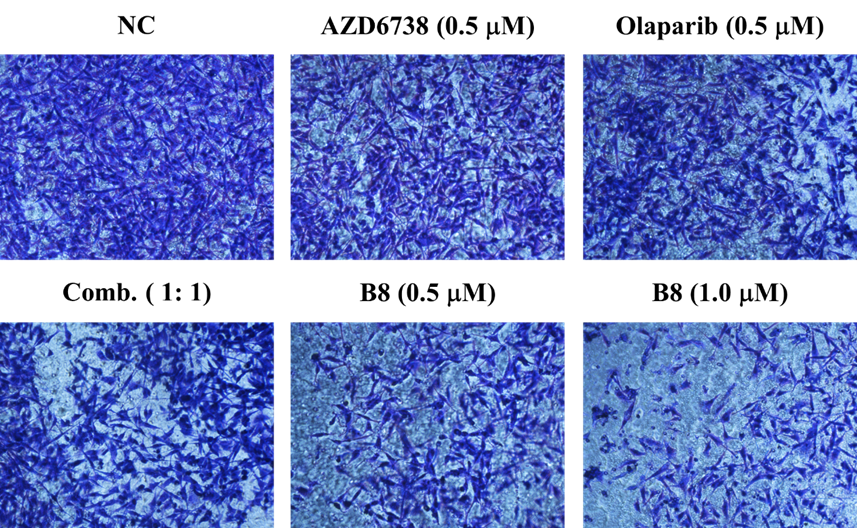
**

**Figure S3**. Representative images of transwell invasion assay in MDA-MB-468 cells for 24 h.

**Part 7: Relative densitometric values**

**Figure S4.** Relative densitometric values of γH2AX, CHK1, p-CHK1, and p-CDK1 protein for **Figure 11D**.

**Part 8: Molecular docking of compound B8**


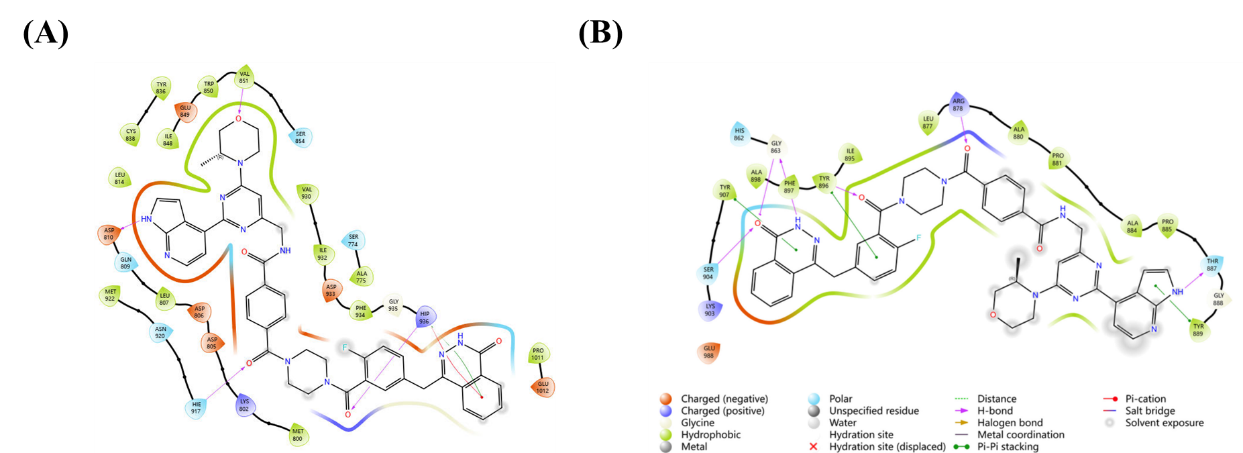


**Figure S5.** Molecular docking and 2D binding modes of compound **B8** with mimetic ATR protein and PARP1 protein respectively. (A) compound **B8** docking using PI3Kα mutant (a mimetic ATR protein, PDB ID: 5UL1). The close view of the key residues from protein with **B8** was presented. (B) compound **B8** docking with PARP1 co-crystal structures (PDB ID: 5DS3). Only key residues from both proteins in close proximity to **B8** were shown.
